# Pangenome analysis reveals the genetic mechanism underlying high‐altitude adaptation in Qinghai–Xizang (Tibet) Plateau *Rhododendron*

**DOI:** 10.1111/jipb.70252

**Published:** 2026-04-12

**Authors:** Haoyang Zhou, Zhongping Xu, Fanhuang Zeng, Haiyu Sang, Zhenhua Liu, Miao Sun, Qiang Fu, Kaige Zhao, Daming Tan, Manzhu Bao, Shuangxia Jin, Xiuqun Liu

**Affiliations:** ^1^ National Key Laboratory for Germplasm Innovation & Utilization of Horticultural Crops Huazhong Agricultural University Wuhan 430070 China; ^2^ Hubei Hongshan Laboratory, National Key Laboratory of Crop Genetic Improvement Huazhong Agricultural University Wuhan 430070 China; ^3^ BioSmartSeek Company Wuhan 430070 China; ^4^ Institute of Vegetables Tibet Academy of Agricultural and Animal Husbandry Sciences Lhasa 850032 China; ^5^ Tibet Academy of Agricultural and Animal Husbandry Sciences Lhasa 850002 China

**Keywords:** high‐altitude adaptation, pangenome, phylogeny, population genetics, *Rhododendron*, structural variation

## Abstract

*Rhododendron*, a globally important group of alpine flowering plants, provides an exceptional system for investigating ecological adaptation and stress resistance owing to its high‐altitude specialization. Using 18 *Rhododendron* species, a graph‐based pangenome was constructed that captures 72,089 nonredundant structural variants. The findings support the integration of subgenus *Azaleastrum* into subgenus *Tsutsusi*, with their comparatively smaller genome sizes likely resulting from the contraction of multiple gene families during lineage differentiation. Gene families specific to high‐altitude *Rhododendron* species were significantly enriched in pathways associated with stress resistance. High‐altitude‐specific long terminal repeat retrotransposons operate through similar regulatory mechanisms, predominantly influencing stress‐responsive genes and promoting adaptive evolution. Through an integrated analysis of population genetics (389 re‐sequenced samples with a mean coverage of 50.2×), transcriptomics, and real‐time quantitative polymerase chain reaction, conserved genes and gene families linked to alpine adaptation in *Rhododendron* were identified. These include genes implicated in cold‐stress responses and ultraviolet (UV) tolerance, such as *CML18*, *CPK1*, *DREB1E*, *LPAT2*, *GPC1*, and *UVR8*. Structural variant profiles within several of these genes offer insights into divergent adaptive mechanisms between high‐ and low‐altitude *Rhododendron* species. The rapid induction of cold‐sensing genes and *CBF*/*DREB1*‐centered cold‐stress signaling pathways indicates an evolutionary adaptation of alpine *Rhododendron* species to low‐temperature habitats. Furthermore, transgenic analyses indicate that cold‐resistance genes, such as *GPC1* derived from high‐altitude *Rhododendron*, markedly improve cold tolerance in *Arabidopsis thaliana* and tobacco. Collectively, this study advances insights into high‐altitude adaptation in ornamental plants and underscores the value of super‐pangenome resources for evolutionary and functional genomics research.

## Graphical abstract

Pan‐genome analysis reveals that high‐altitude *Rhododendron* species resist alpine cold stress by rapidly sensing and engaging the chilling response pathway and genes that directly and indirectly protect the plant from UV radiation. Heritable genomic features such as long terminal repeats contribute to the adaptive diversification of *Rhododendron* species.

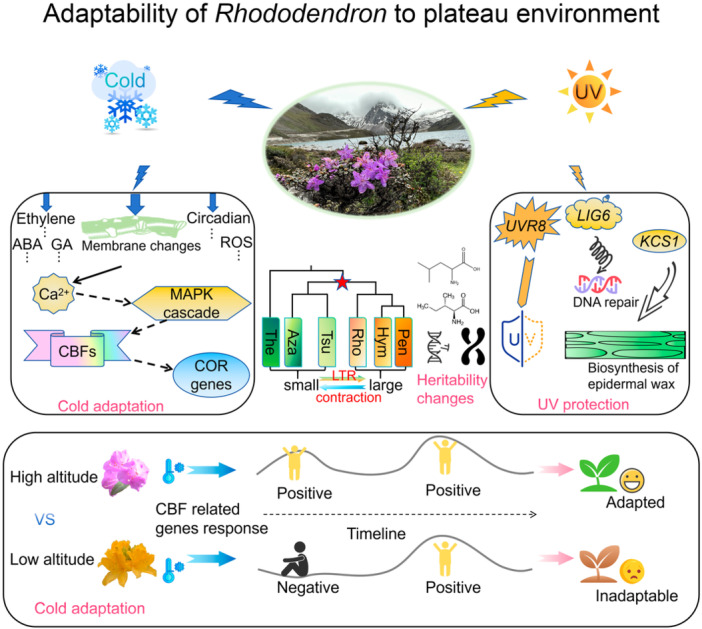

## INTRODUCTION

The tectonic uplift of the Qinghai–Xizang (Tibet) Plateau, combined with its distinctive Asian monsoon climate, has created a biodiversity hotspot that supports numerous animal and plant species (Huang et al., 2015; He et al., 2022). However, the region's extreme environmental conditions, including cold stress, temperature variability, hypoxia, and intense ultraviolet (UV) radiation (Qiao et al., 2016), pose significant challenges to lowland species. Indigenous species likely evolved genetic adaptations in response to these environmental challenges. For instance, humans show a highly differentiated haplotype architecture of *EPAS1*, which enables Tibetans to tolerate hypoxic conditions (Huerta‐Sánchez et al., 2014). Population genetic analyses and resequencing studies have identified key genes and metabolic pathways underlying high‐altitude adaptation in species such as Tibetan wild boars, Tibetan mastiffs, Tibetan chickens, and yaks (Li et al., 2013, 2014; Qiu et al., 2015; Wang et al., 2015). Moreover, comparative genomics and transcriptomic approaches have revealed candidate genes associated with DNA repair and hypoxia responses in Tibetan antelope, snub‐nosed monkeys, and ectothermic snakes (Ge et al., 2013; Yu et al., 2016; Li et al., 2018). Similar investigations have been conducted in diverse alpine herbs and a limited number of tall perennial trees (Zhang et al., 2016; Zeng et al., 2018; Guo et al., 2020), including *Crucihimalaya himalaica* (Zhang et al., 2019a) and Tibetan *Prunus* species (Wang et al., 2021a). The adaptive mechanisms of various plant ecotypes to severe conditions may vary; however, the adaptive evolution of shrubs under plateau conditions has received relatively little attention.

*Rhododendron*, a hallmark of the Sino–Himalayan flora, is the most speciose shrub genus in the Northern Hemisphere, encompassing roughly 1,200 species globally (Chen et al., 2018b; Khan et al., 2021). The genus is broadly distributed across Asia, Europe, and North America, including the Himalayas, Alps, Rocky Mountains, and Appalachian Mountains, making it an excellent system for investigating high‐altitude adaptation. The Qinghai–Xizang (Tibet) Plateau (QXP) in the Himalayas is one of the three major *Rhododendron* distribution centers globally. The genus encompasses both evergreen (~90%) and deciduous (~10%) species (Xia et al., 2022), displaying diverse morphologies and substantial ornamental and practical value. For instance, *R. molle* and *R. delavayi* serve medicinal purposes, whereas species such as *R. nivale*, *R. principis*, and *R. laudandum* form expansive natural assemblages that enhance soil stability and water conservation in plateau ecosystems. In particular, within Xizang, these species are essential for mountain ecosystem stability. To date, genomic research on *Rhododendron* has primarily focused on phylogenetics (Zhang et al., 2017; Soza et al., 2019; Shirasawa et al., 2021; Ma et al., 2022; Zhou et al., 2022a; 2022b) and the characterization of specific germplasm (Ma et al., 2021; Chang et al., 2023; Wen et al., 2024) and populations, including flower color (Yang et al., 2020; Wu et al., 2023; Xia et al., 2024) and ploidy (Lyu et al., 2024). In addition, some studies have examined the adaptability of *Rhododendron* species to low‐altitude environments (Wang et al., 2021b). Structural variations (SVs) across the genome can substantially influence plant phenotypes (Wang et al., 2023; Khan et al., 2024), as demonstrated in soybeans (Liu et al., 2020) and potato (Zhu et al., 2025). Advances in sequencing technologies have enabled the construction of *Rhododendron* pangenomes, providing a powerful framework for examining the genetic basis of adaptation. A pangenome was constructed using nine *Rhododendron* species and environmental genome‐wide association studies (enGWAS) to examine alpine adaptability in *R. nivale* populations (Xia et al., 2024). More recently, a pangenome incorporating 15 *Rhododendron* species was developed using the telomere‐to‐telomere (T2T) assembled *R. liliiflorum* genome, and heat tolerance mechanisms in the genus were elucidated using transcriptomic profiling (Wang et al., 2025). Despite these advances, the karyotype evolution of *Rhododendron*, the shared adaptive features of high‐latitude species, and the differences between these and low‐altitude azaleas remain poorly understood.

In the present study, the whole genomes of seven *Rhododendron* species were sequenced, and a comprehensive pangenome was constructed by integrating them with 11 previously published genomes. Analysis of 18 *Rhododendron* genome assemblies alongside 389 resequenced accessions provides a significantly improved resource for future research on this genus. This study identifies adaptive commonalities among high‐altitude *Rhododendron* species by combining structural variation analysis, altitude‐specific metabolic pathway enrichment assessment, and multimodel population genetic analyses. Transcriptomic profiling combined with RT‐qPCR validation identified key differences in cold tolerance mechanisms between high‐ and low‐altitude *Rhododendron*. These findings elucidate both shared and species‐specific adaptive mechanisms in *Rhododendron*, thereby expanding the study of adaptation in this genus.

## RESULTS

### *De novo* sequencing, assembly, and annotation of the seven *Rhododendron* genomes

**Figure 1:**
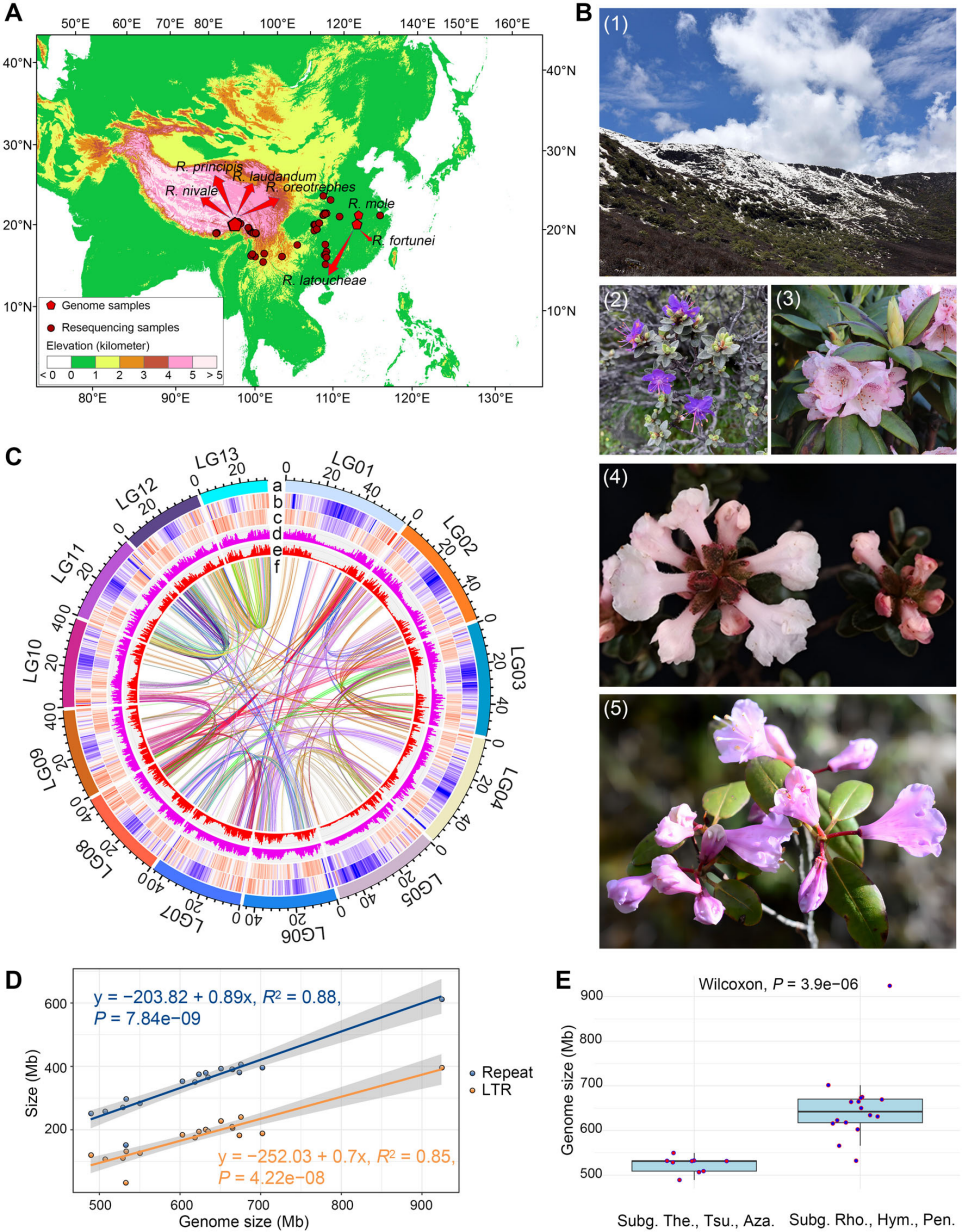
**Collection of** ***Rhododendron*** **samples and genome sequencing** **(A)** Geographic distribution of the *Rhododendron* accessions analyzed in this study. The brown dots indicate the sampling locations for re‐sequencing, whereas the red pentagons denote the sampling sites for whole‐genome sequencing. The size of the pentagon corresponds to the number of samples collected at each site. **(B)** Representative *Rhododendron* species from the Xizang (Tibetan) Plateau. (1) Typical growth habitat of *Rhododendron* in Xizang; (2–5) flowers of *R. nivale*, *R. principis*, *R. laudandum*, and *R. oreotrephes*. **(C)** Overview of the *R. oreotrephes* genome assembly. (a) Thirteen pseudochromosomes; (b) gene density; (c) repeat sequence density; (d) single‐nucleotide polymorphism (SNP) density; (e) insertion–deletion (Indel) density; and (f) collinear genomic blocks. The graphical layout is consistent with Figure S1. **(D)** Correlation between repeat content (or LTR) and genome size across 18 *Rhododendron* genomes. The *P*‐value was calculated using a two‐tailed student's *t*‐test based on the Pearson correlation coefficient (the *t*‐test was used specifically to assess whether the Pearson correlation coefficient differs significantly from zero). **(E)** Comparison of genome sizes among different *Rhododendron* subgenera. The., subg. *Therorhodion*; Tsu., subg. *Tsutsusi*; Aza., subg. *Azaleastrum*; Rho., subg. *Rhododendron*; Hym., subg. *Hymenanthes;* and Pen., subg. *Pentanthera*.

**Figure 2:**
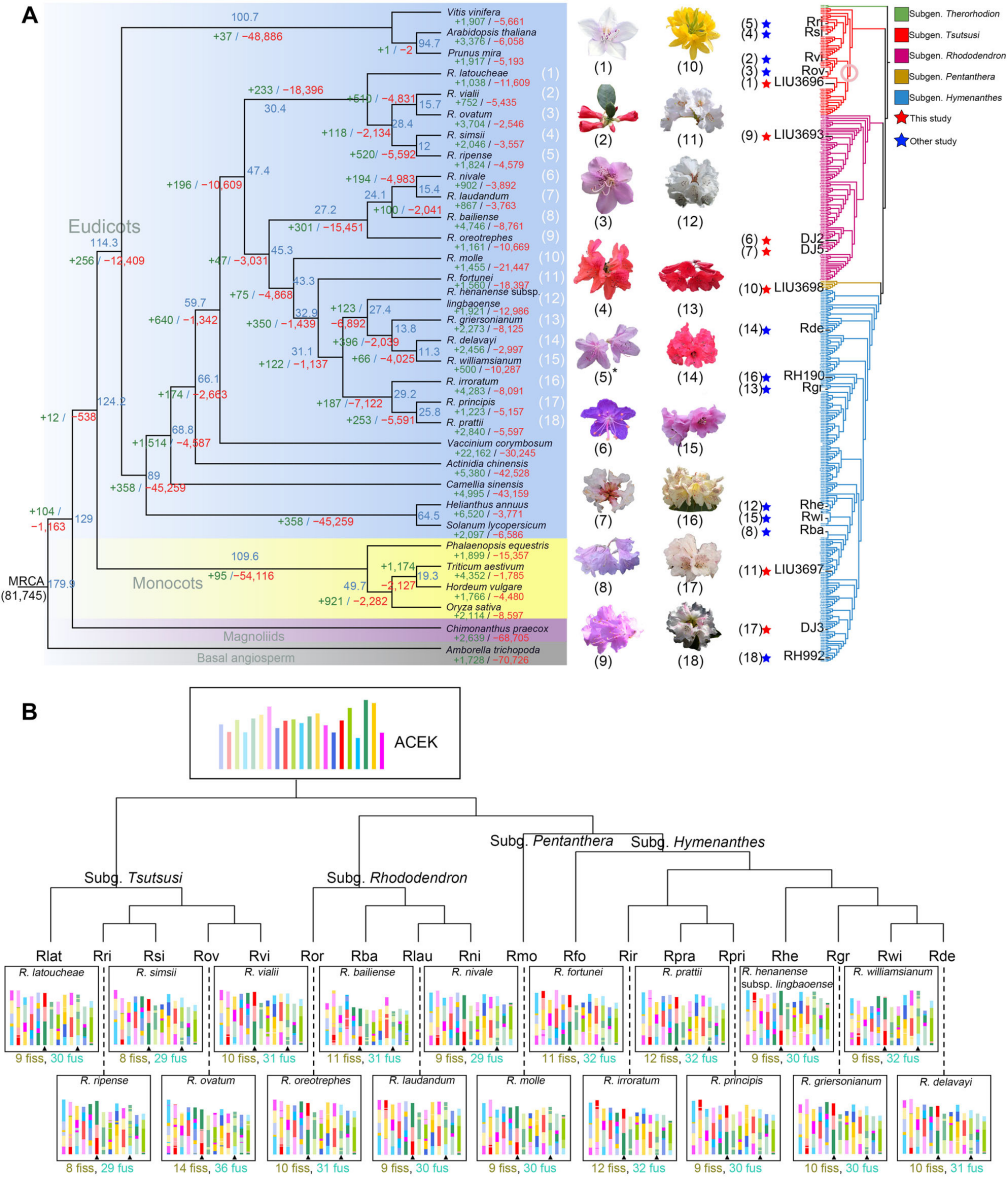
Comparative genomic analyses and karyotype evolution **(A)** Left: phylogenetic tree of 32 angiosperms. Blue numbers indicate estimated divergence times at each node (Mya), whereas green and red denote the total number of expanded and contracted orthologs, respectively. Middle: photographs of 18 *Rhododendron* species. Right: phylogenetic tree of 389 *Rhododendron* accessions based on SNP. The tree was constructed using maximum likelihood (ML) analysis of SNPs from single‐copy nuclear gene regions shared among the 389 accessions. The 18 accessions included in the pangenome (indicated by pentagrams) occupy representative positions in the phylogenetic tree. The clade marked by pink circles corresponds to subgenus *Azaleastrum*. **(B)** Karyotype evolution model of *Rhododendron* species. Modern genome structures are depicted at the bottom, with colors corresponding to Ancestral Core Eudicot karyotypes.

Seven *Rhododendron* species were selected for *de novo* genome sequencing based on their distinct geographical distributions and research relevance (Figure 1A, B). Four of these samples were collected from Xizang and originated from substantially higher altitudes than those of the remaining species. The estimated heterozygosity levels of the seven genomes ranged from 1.00% to 1.90%. The predicted genome sizes of the seven *Rhododendron* accessions ranged from 549.18 to 657.75 Mb. Each accession was independently sequenced using three complementary technologies: DNBSEQ‐T7, with an average coverage depth of 56×; single‐molecule real‐time sequencing, with an average depth of 52×; and high‐throughput chromosome conformation capture (Hi‐C) sequencing, with an average coverage depth of 100× (Tables S1, S2). Genomes were separately assembled for the seven accessions. Remapping of next‐generation sequencing reads from a single germplasm to the corresponding assembled genome yielded mapping rates of 97.14%–99.70%, while PacBio reads achieved > 99% mapping for each genome, demonstrating high continuity and completeness of the assemblies (Tables S1, S2). Hi‐C data were subsequently used to anchor contigs into scaffolds, resulting in chromosome‐level assemblies ranging from 489.17 to 664.61 Mb, with a mean contig N50 of 24.01 Mb and a mean scaffold N50 of 45.69 Mb (Table S3). The assembled genome sizes closely matched prior estimates, with 95.36%–97.93% of sequences successfully anchored to 13 pseudochromosomes (2*n* = 26). The contigs demonstrated robust clustering, ordering, and orientation (Figures 1C, 2, and S1; Table S3). Benchmarking universal single‐copy orthologs (BUSCO) analyses revealed an average of 98.38% complete gene elements across the seven genomes, confirming the high reliability and quality of the assemblies (Table S4).

Subsequently, protein‐coding genes were annotated for each genome, yielding 33,438–37,399 genes with average coding sequence (CDS) lengths of 1,085.05–1,148.01 bp (Table S5). Repetitive sequences accounted for an average of 58.25% of each genome, whereas transposable elements (TEs) contributed 36.38%–46.35% (Table S6). Correlation analysis indicated that both repeat sequence size and long terminal repeat (LTR) abundance were positively correlated with genome size (Figure 1D; Table S6), implying that transposons, particularly LTRs, are a major determinant of genome size. The predicted noncoding ribonucleic acids (ncRNAs) included 1,723–4,454 rRNAs, 1,316–1,713 small RNAs, 5–15 regulatory RNA, and 481–723 transfer RNAs (Table S7). The annotated BUSCO scores ranged from 94.92% to 97.40%, confirming the successful assembly of conserved genes and demonstrating the high reliability of the genome annotations (Table S4).

### Phylogenetic and evolutionary genome analyses

Data from the seven *Rhododendron* species analyzed in this study were integrated with genome data from 11 additional representative *Rhododendron* species. These species are indigenous to Asia and span an expansive range of altitudinal distributions (Figure S3). To elucidate the evolutionary relationships of *Rhododendron*, a phylogenetic tree was constructed using 490 low‐copy number (LCN) orthologs identified from 32 angiosperm genomes, including four monocots, 26 eudicots (21 Ericales), one magnoliid, and one basal angiosperm (Figures 2A, S4). The resulting topology was consistent with the Angiosperm Phylogeny Group IV classification. Molecular clock analysis estimated that *Rhododendron* and *Vaccinium* diverged approximately 57.7–62.8 million years ago (Mya; 95% confidence interval [CI]). The two ancient subgenera, *Tsutsusi* and *Azaleastrum*, diverged around 47.4 (39.9–56.1) Mya from the last common ancestor of the remaining 13 *Rhododendron* species. After this divergence, *Rhododendron* genomes expanded markedly (Figure 1E), possibly associated with a contemporaneous decline in atmospheric oxygen levels during the same period. Research has shown a significant association between variation in plant genome size and ecological adaptation (Ranaivoson et al., 2026). Phylogenetic evidence from both LCN orthologs and SNPs supports the integration of subgenus *Azaleastrum* (pink circle‐marked clade) into subgenus *Tsutsusi* (Figure 2A). During the emergence of this subgenus, 18,396 gene families experienced contraction, potentially contributing to its reduced genome size. Subsequently, the subgenus *Rhododendron* diverged before the subgenera *Pentanthera* and *Hymenanthes* split, with the latter pair separating approximately 43.3 Mya.

Gene family expansion and contraction patterns at key evolutionary nodes were investigated using Kyoto Encyclopedia of Genes and Genomes (KEGG) pathway analysis. The findings revealed that contracted gene families were primarily enriched in pathways related to terpenoid and polyketide metabolism (e.g., zeatin biosynthesis), amino acid metabolism, and DNA replication and repair processes during subgenus *Tsutsusi* differentiation (Figure S5). Gene family expansion and contraction patterns were further compared among three *Rhododendron* species inhabiting extremely high‐altitude environments (> 4,000 m above sea level), revealing a shared evolutionary trend between *R. nivale* and *R. laudandum*. In both species, expanded gene families were substantially overrepresented in pathways associated with alanine, aspartate, and glutamate metabolism, whereas contracted gene families were predominantly enriched in betalain and indole alkaloid biosynthesis (Figure S6). In contrast, *R. principis* displayed a unique pattern, characterized by contractions principally linked to sesquiterpenoid and triterpenoid biosynthesis and expansions markedly enriched in phenylpropanoid biosynthesis (Figure S7). *R. nivale* and *R. laudandum* are low‐growing shrubs. Notably, *R. principis* displays a taller phenotype, which may contribute to differences in gene family expansion and contraction patterns relative to low‐growing species such as *R. nivale* and *R. laudandum*.

To examine whole‐genome duplication (WGD) events in the evolutionary history of *Rhododendron*, synteny analyses were performed across nine representative *Rhododendron* species encompassing all five recognized subgenera. A total of 10,058 paralogous blocks comprising 95,690 genes were identified. The evaluation of synonymous substitution rates (*K*s) among gene pairs identified two distinct peaks across all nine accessions, which is in agreement with findings reported in earlier studies (Zhang et al., 2017; Soza et al., 2019; Yang et al., 2020; Ma et al., 2021; Shirasawa et al., 2021; Wang et al., 2021b; Zhou et al., 2022a; 2022b; Figure S8A). Subsequently, *K*s values from *Rhododendron* were compared with those obtained from outgroup species, including *Actinidia chinensis*, *Camellia sinensis*, *Vitis vinifera*, *Arabidopsis thaliana*, and *Solanum lycopersicum*. The WGD patterns observed in these outgroups were consistent with previously reported results (Jaillon et al., 2007; Shi et al., 2010; Song et al., 2012; Amborella Genome Project, 2013; Bomblies and Madlung, 2014; Wei et al., 2018; Figure S8B).

Additionally, dotplot analyses of paralogous gene pairs across the nine *Rhododendron* genomes identified a syntenic block on the chromosome with five additional copies located at corresponding positions across all genomes (Figures S9, 10). This 1:6 copy number ratio indicates that *Rhododendron* experienced an ancient whole‐genome triplication (WGT) event (*K*s = 1.51–1.59; mean = 1.54), followed by a more recent WGD event (*K*s = 0.70–0.76; mean = 0.72; Figures S8A, S9, and S10). Conversely, as previously reported, the *Actinidia chinensis* (kiwifruit) genome has undergone one WGT event and two WGD events, whereas the *Vitis vinifera* (grape) genome has experienced only a single WGT event (Jaillon et al., 2007; Shi et al., 2010). Comparative synteny analyses between *Rhododendron* and kiwifruit and between *Rhododendron* and grape revealed clear 2:4 and 2:1 syntenic relationships, respectively, further supporting the conclusion that two WGD events occurred in the *Rhododendron* lineage (Figure S11).

The timing of WGD events in *Rhododendron* was estimated using Ks values derived from *R. principis*, *R. vialii*, *C. sinensis*, and *V. vinifera* syntenic homeologs (Figure S8C). The rate of synonymous substitutions per site per year was determined using the formula *T* = *K*s/2*r*, based on a divergence time of 118.0 Mya between asterids (represented by tea and rhododendron) and rosids (represented by grape). These analyses revealed that the most recent WGD event occurred during the Upper Cretaceous at approximately 87.6 Mya (CI: 82.7–92.0 Mya), whereas the more ancient WGT event occurred in the Lower Jurassic at 187.4 Mya (CI: 176.9–196.7 Mya).

Furthermore, the evolutionary trajectory of *Rhododendron* karyotypes was reconstructed from ACEK to their extant configurations using orthologous genes from 18 *Rhododendron* genomes in conjunction with the Ancestral Core Eudicot Karyotypes (ACEK). Throughout their evolution, these 18 *Rhododendron* species experienced 8–14 chromosomal fissions and 29–36 fusions, resulting in a change from the ancestral karyotype (*n* = 21) to the present karyotype (*n* = 13) (Figures 2B, S12–S20). No consistent pattern of chromosomal fission or fusion was observed within or among subgenera, and the underlying processes were broadly conserved among species, except in *R. ovatum*, which displayed an increased frequency of chromosomal rearrangements. In *R. latoucheae*, the ancestral chromosomes A2, B2, C3, D3, E3, F1, F2, G1, and G3 each undergo a single break, producing two distinct chromosomes per event. Comparative alignment of chromosomes across all species using *R. latoucheae* as a reference revealed a conserved fusion mechanism for chromosomes 6 and 11 throughout the genus. Chromosome 6 originated through the breakage, fusion, and inversion of E3, B1, and B3, whereas chromosome 11 arose through the analogous rearrangements of C2, D1, C1, and E2 (Figure S21).

### Graph‐based analysis of the *Rhododendron* pangenome

**Figure 3:**
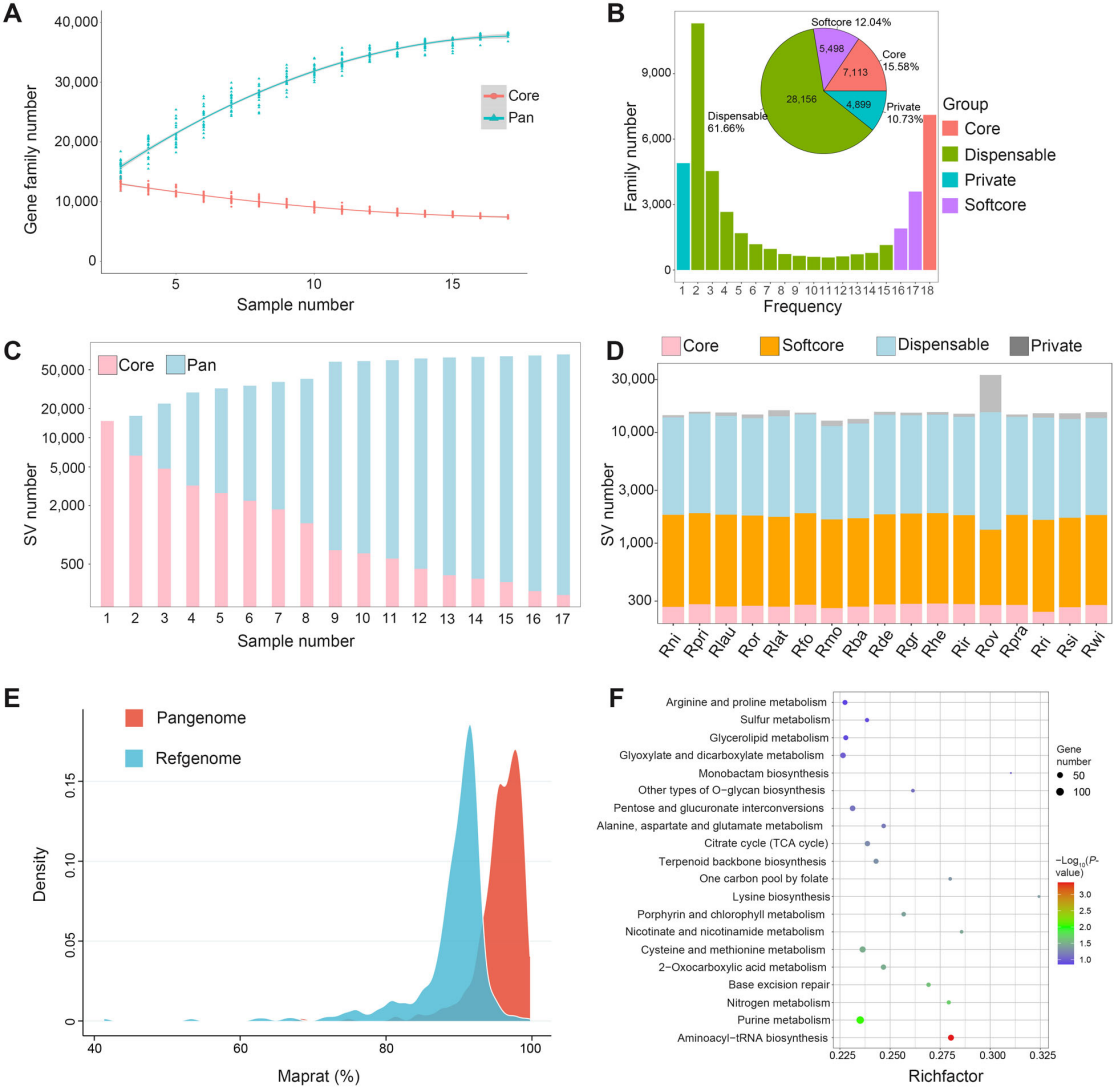
**Pangenome of** ***Rhododendron*** **species** **(A)** Variation in gene families as additional genomes are incorporated, showing trends in pan and core gene sets. **(B)** Compositions of identified pan genes. The histogram depicts the number of gene families with varying frequencies across the 18 genomes. The pie chart illustrates the proportion and specific counts of each gene family. **(C)** Number of variants per accession in each discovery class. **(D)** Counts of different SVs in each genome. Abbreviations: Rni—*Rhododendron nivale*, Rpri—*R. principis*, Rlau—*R. laudandum*, Ror—*R. oreotrephes*, Rlat—*R. latoucheae*, Rfo—*R. fortunei*, Rmo—*R. molle*, Rba—*R. bailiense*, Rde—*R. delavayi*, Rgr—*R. griersonianum*, Rhe—*R. henanense* subsp. *lingbaoense*, Rir—*R. irroratum*, Rov—*R. ovatum*, Rpra—*R. prattii*, Rr—*R. ripense*, Rsi—*R. simsii*, and Rwi—*R. williamsianum*. **(E)** Genetic coverage of the pangenome. Re‐sequencing data were aligned to both the reference genome and the *Rhododendron* pangenome, demonstrating that the pangenome encompasses the majority of genetic variation present in the resequenced accessions. **(F)** KEGG enrichment of core gene families.

The high‐contiguity genome assemblies generated in this study enabled a comprehensive comparison of genetic variations across 18 *Rhododendron* species and supported the development of a graph‐based pangenome for the genus. Among these assemblies, *R. vialii* showed the highest assembly completeness and the lowest number of gaps, indicating that it is the optimal reference genome. The *Rhododendron* pangenome was constructed by grouping 45,666 nonredundant gene clusters from 18 genomes. With the incorporation of additional genomes, both the core and pan gene sets approached plateauing (Figure 3A). The gene clusters were classified into four categories based on their distribution across accessions: 28,156 dispensable gene families (present in 2–15 accessions), representing the largest proportion (61.66%); 7,113 core gene families (found in all 18 accessions); 5,498 softcore gene families (present in 16–17 accessions); and 4,899 private gene families (restricted to a single accession) (Figure 3B; Table S8). Genetic data from resequenced accessions were aligned to both the reference genome and the pangenome to evaluate pangenome representativeness. On average, the pangenome captured 95.61% of the genetic diversity within the resequencing population, compared with 88.97% for the reference genome (Figure 3E; Dataset S1). These findings highlight the superior capacity of the pangenome to encompass genetic diversity relative to a single high‐quality reference genome. Gene ontology (GO) analysis indicated that core gene families were markedly overrepresented in cellular processes associated with intracellular architecture, cellular compartments, and membrane‐bound organelles, highlighting their primary role in the maintenance of cellular structural integrity (Dataset S2). Dispensable gene clusters were markedly enriched in molecular functions, including ion binding, carbohydrate derivative binding, and purine ribonucleotide binding (Dataset S2). Similarly, KEGG pathway analysis demonstrated that core genes were significantly enriched in pathways related to translation, nucleotide metabolism, and cofactor and vitamin metabolism, including aminoacyl‐tRNA biosynthesis and purine metabolism (Figure 3F; Dataset S3). Private gene clusters were enriched in specialized metabolic pathways, including biosynthesis of sesquiterpenoid and triterpenoid and circadian rhythm regulation (Dataset S3).

Statistical analyses of the altitudinal distribution of the 18 *Rhododendron* species included in the pangenome indicated that nine species predominantly occur at elevations above 2,000 m (Figures S3, S22; Dataset S4). Accordingly, *Rhododendron* species were classified into high‐altitude (hereafter H) and low‐altitude (hereafter L) groups using 2,000 m as the elevation threshold, and altitude‐specific gene sets were subsequently identified (as described in Methods). This classification yielded 7,862 gene families distinct to the high‐altitude group and 5,428 gene families unique to the low‐altitude group. GO analysis indicated that high‐altitude‐specific genes were primarily associated with nucleotide‐binding and membrane‐related processes, whereas low‐altitude‐specific genes were primarily associated with nuclease activity and ion binding (Dataset S5). Further KEGG pathway analysis demonstrated that high‐altitude *Rhododendron* species were significantly enriched in pathways involved in the metabolism of cofactors, vitamins, amino acids, and carbohydrates (Figure S23; Dataset S6). Genes involved in glycolysis and gluconeogenesis were significantly enriched, indicating an enhanced capacity for energy metabolism in response to stress. Conversely, genes specific to low‐altitude *Rhododendron* species were predominantly enriched in pathways associated with terpenoid and polyketide biosynthesis and environmental response pathways, including sesquiterpenoid and triterpenoid biosynthesis and plant–pathogen interactions (Figure S23; Dataset S6).

Seven *de novo* assembled genomes and 10 previously published genomes were anchored to the reference genome, resulting in the identification of 1,309,995 SNPs, 84,987 Indels ≤ 50 bp, 60,662 presence/absence variants (PAVs) (Indels > 50 bp), 4,799 copy number variations (CNVs), 1,540 translocations (including 337 intrachromosomal and 1,203 interchromosomal events), and 5,088 inversions (Figures S24–S26; Tables S9–S11). PAVs in *R. ovatum* resulted in the deletion of 27.99 Mb of genomic sequences (Table S9), indicating that SVs can substantially influence genome size. Consistent with the patterns observed for pan genes, the total number of SVs increased with the inclusion of additional genomes before reaching saturation, whereas the number of core SVs declined (Figure 3C). Ultimately, 240 SVs were shared across all accessions (Table S12). Subsequently, the identified SVs were categorized into four classes—core, softcore, dispensable, and private—with dispensable and private accounting for the majority of SVs in each accession (Figure 3D; Table S13). Synteny analysis revealed that *R. ovatum* showed a higher frequency of chromosomal translocations than other *Rhododendron* species (Figures S24–S26), likely reflecting an increased number of chromosomal rearrangements during its evolution (Figure 2B), and resulted in a greater number of species‐specific SVs (Figure 3D). To assess the effect of SVs on CDS, KEGG pathway analyses were performed on genes in which at least 50% of the CDS was affected by SVs. These genes were significantly enriched in pathways associated with the processing of genetic information, including spliceosome activity and basal transcriptional machinery, as well as in cellular processes such as eukaryotic autophagy (Dataset S7), indicating that SV‐affected genes contribute to fundamental metabolic and regulatory functions in *Rhododendron*.

LTRs are recognized as a primary contributor to variation in genome size and are likely to modulate the expression of downstream genes (Zhang et al., 2019b). Analysis of LTR insertion times across the 18 *Rhododendron* species included in the pangenome revealed that insertion peaks, including recent peaks observed in species with biphasic patterns, occurred between 0.08 and 0.25 Mya, with the exception of *R. williamsianum* (Figure S27). These findings indicate that LTR acquisition across different subgenera and altitudinal ranges was largely synchronous, occurring in the middle to Late Pleistocene. In this study, both *R. simsii* and *R. molle* occur at low altitudes but show substantial differences in genome size (528.64 Mbp vs. 650.25 Mbp), primarily attributable to LTR content variations. *R. principis* and *R. fortunei*, although classified within the same subgenus (*Hymenanthes*) and possessing comparable genome sizes, occupy vastly different altitudinal ranges. Species‐specific or additional LTR insertions may contribute to divergence or modulate adaptive capacity by affecting downstream gene expression. Two comparative groups were selected to evaluate the effect of unique LTRs on *Rhododendron* species: *R. simsii* (low altitude, small genome) versus *R. molle* (low altitude, large genome), and *R. principis* (high altitude) versus *R. fortunei* (low altitude). Species‐specific LTRs were detected in these comparisons, and KEGG pathway analyses were conducted on genes situated within 1 kb of the LTRs. Genes associated with unique LTRs in *R. simsii* (small genome) were predominantly enriched in pathways related to amino acid metabolism, carbohydrate metabolism, and secondary metabolite biosynthesis, including valine, leucine, and isoleucine biosynthesis (Figure S28; Dataset S8). In contrast, the genes impacted by the expanded LTR content in *R. molle* (large genome) were primarily enriched in lipid metabolism, amino acid metabolism, and nucleic acid‐associated pathways, including replication and repair mechanisms such as homologous recombination, the mRNA surveillance pathway, and mismatch repair (Figure S28; Dataset S8). The accumulation of extra genetic material in *R. molle* after species divergence may help sustain genetic diversity within the species. Genes impacted by distinct LTRs were significantly enriched in pathways associated with lipid, carbohydrate, and amino acid metabolism in *R. principis* (high altitude). These included the biosynthesis of unsaturated fatty acids, cutin, suberin, and wax, as well as the metabolism of starch, sucrose, and cyanoamino acids (Figure S29; Dataset S8). These pathways are associated with cold and UV stress tolerance, serving as essential adaptive mechanisms for survival at high altitudes. Genes impacted by high‐altitude‐specific LTRs in *Rhododendron* display pathway enrichment patterns that closely reflect those of species‐specific gene families in high‐altitude species. Conversely, genes in *R. fortunei* (low altitude) were significantly enriched in pathways associated with environmental adaptation, nucleic acid replication and repair, and terpenoid and polyketide biosynthesis (encompassing plant–pathogen interactions and diterpenoid biosynthesis) (Figure S29; Dataset S8).

### Population genetic studies revealed genomic commonalities underlying high‐altitude adaptation in *Rhododendron*

**Figure 4:**
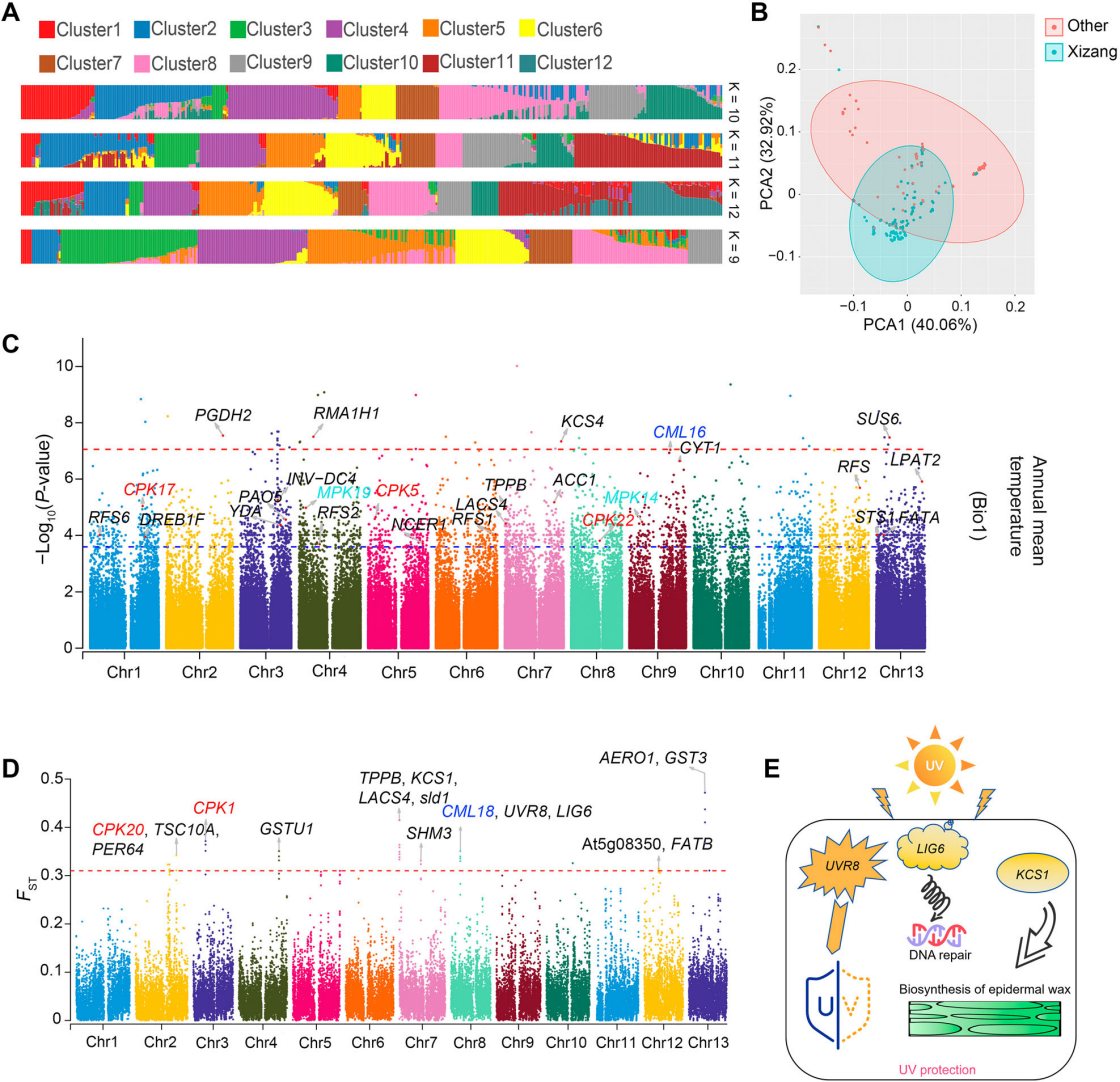
**Genetic analyses of** ***Rhododendron*** **populations** **(A)** Population structure inferred from 389 resequenced samples. **(B)** PCA of genome‐wide variation in *Hymenanthes*. The green and red circles denote accessions from Xizang and other regions, respectively. **(C)** Manhattan plot of the LFMM results based on BIO1. **(D)** Manhattan plot of the genetic differentiation analysis. The regions above the dashed line represent the top 1% of the empirical *F*_ST_ distribution (*F*_ST_ = 0.31). **(E)** Candidate genes involved in UV stress tolerance.

Variations in allele frequencies among populations may be associated with environmental conditions (Aguirre‐Liguori et al., 2021). Populations native to the Qinghai–Xizang (Tibet) Plateau are exposed to harsher environmental pressures than *Rhododendron* species from other regions. These populations may have developed strong, conserved adaptive mechanisms at the genomic level. To explore shared genetic adaptations, 101 samples were resequenced using next‐generation sequencing technology, attaining an average sequencing depth of 49.1× per accession (Dataset S1). A total of 1,419,326 SNPs and 94,476 Indels were identified across 389 resequenced samples (including 288 high‐quality samples from published data sets). A latent factor mixed‐model (LFMM) analysis was performed using 19 precipitation‐ and temperature‐related variables to investigate environmental‐related genomic variations. The population structure was examined through admixture analysis, and the optimal number of ancestral clusters (*K*) was determined by assessing cross‐validation (CV) errors across varying *K* values. The results revealed that *K* = 10 minimized the CV error, indicating the optimal population structure (Figures 4A, S30A). Subsequently, this *K* value was adopted as the optimal number of latent factors for the LFMM analysis. To further assess the influence of the environmental variables on population genetic structure and to determine allele frequency changes along climatic gradients, a gradient forest (GF) analysis was performed to rank the relative importance of the 19 climate‐related variables. The analyses indicated that five temperature‐associated variables—Bio10 (mean temperature of the warmest quarter), Bio8 (mean temperature of the wettest quarter), Bio5 (maximum temperature of the warmest month), Bio1 (annual mean temperature), and Bio11 (mean temperature of the coldest quarter)—exerted the strongest influence on genetic differentiation (Figure S30B). Correspondingly, pronounced shifts in allele composition were observed across gradients of these climatic variables (Figure S31). Finally, LFMM analysis was conducted using these five temperature variables in conjunction with geographic coordinates, elevation, and SNP data derived from the resequenced populations, resulting in the identification of 27 loci significantly correlated with temperature across three variables (Figures 4C, S32). These 27 candidate genes mediate temperature responses primarily through signal transduction, membrane lipid stabilization, osmotic adjustment, oxidative stress responses, and transcriptional regulation pathways. For instance, *CML16* functions as a putative calcium sensor implicated in cold sensing, whereas *CPK17* and *CPK22* mediate calcium‐dependent cold signal transduction. *MPK14* and *MPK19* are components of the mitogen‐activated protein kinase (MAPK) cascade, whereas *DREB1F*, a member of the *DREB1* gene family, is widely recognized for its role in cold tolerance. *LACS4* catalyzes the activation of long‐chain fatty acids and maintains membrane lipid stability. *RFS6*, *TPPB*, and *STS1* control the biosynthesis of raffinose, trehalose, and oligosaccharides, respectively, thereby facilitating osmotic homeostasis during cold stress. *PAO5* mediates the production of hydrogen peroxide (H_2_O_2_) under cold‐stress conditions (Figure 4C). Notably, *RFS6* and *STS1*, both involved in sugar biosynthesis, exclusively carried reference alleles in all population samples from the QXP and surrounding mountain regions, which are characterized by lower mean annual temperatures, whereas their mutant alleles were confined to populations from regions with higher mean annual temperatures (Figure S33A). The reference allele (A) of *PAO5* was primarily present in QXP *Rhododendron* populations, whereas the mutant allele (T) was largely confined to populations from other regions (Figure S33B). For *LACS4*, the mutant allele (A) was largely concentrated in populations from the Tibetan Plateau, whereas the G allele predominated in populations from other regions (Figure S33C). Additionally, two candidate genes were shared between Bio1 and Bio11: *PGDH2*, positioned on chromosome 2 and involved in serine biosynthesis, and *RMA1H1*, situated on chromosome 4 and implicated in aquaporin regulation (Figures 4C, S32). In Bio5, *FATB1*, a gene essential for *de novo* fatty acid synthesis, shared a similar function with *FATA*, a gene associated with Bio1 (Figures 4C, S32).

**Figure 5:**
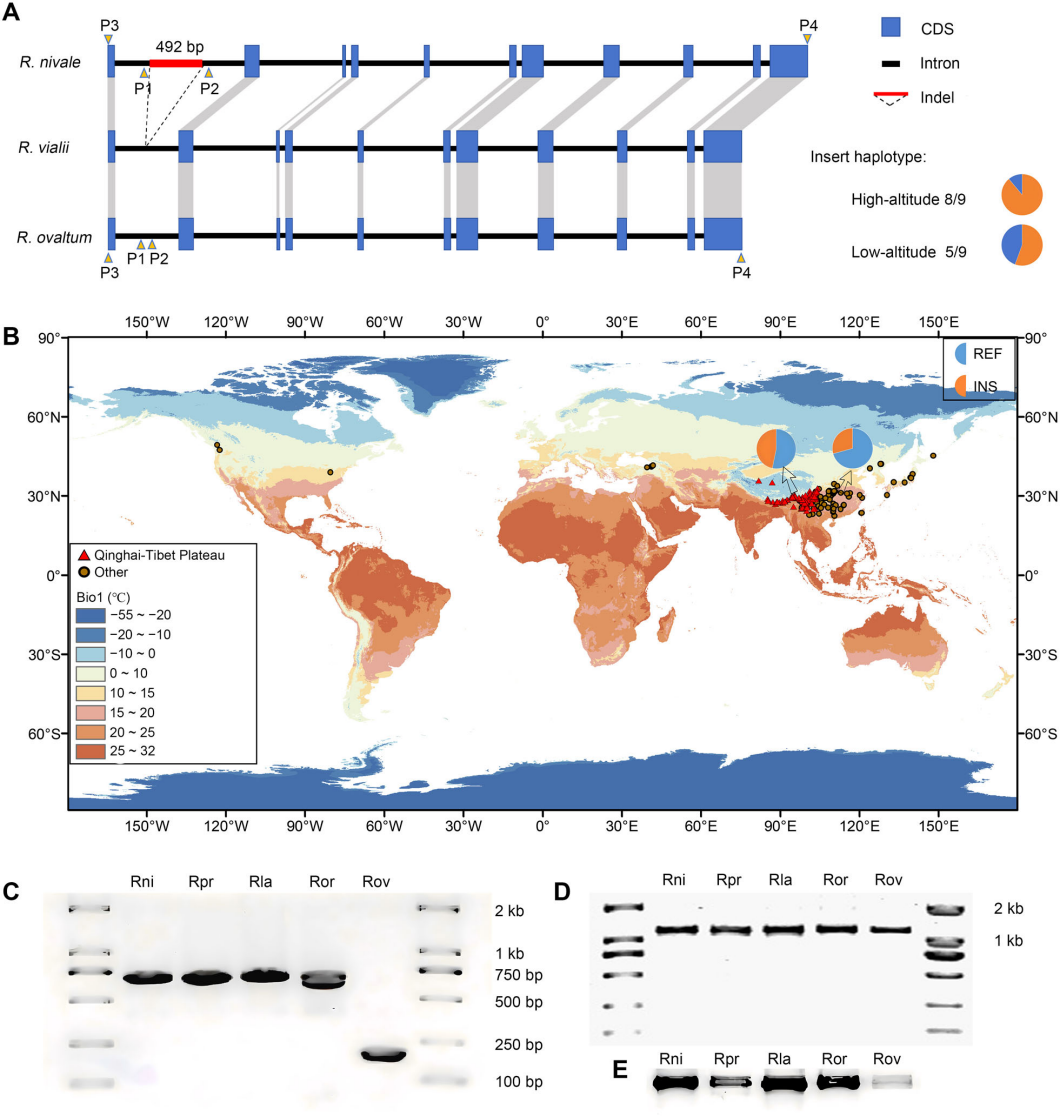
**Structural variations in** ***LPAT2*** **across high‐ and low‐altitude** ***Rhododendron*** **species** **(A)** Location and distribution of structural variations (SVs) in the *LPAT2* gene across high‐ and low‐altitude *Rhododendron* species. **(B)** Frequency distribution of BIO1‐associated SVs in *LPAT2* across resequenced populations from the QXP and other regions. Colors represent variations in the relevant climate variables across the distribution range. REF, reference type, consistent with the reference genome, and absence of SV within the gene. INS, Insertion type, SV insertion present within the gene. **(C)** PCR amplification of genomic DNA (gDNA) at p1‐p2 loci in high‐ and low‐altitude *Rhododendron* species. Rni—*R. nivale* (H), Rpr—*R. principis* (H), Rlau—*R. laudandum* (H), Ror—*R. oreotrephes* (H), and Rov—*R. ovatum* (L). **(D)** PCR amplification of complementary DNA (cDNA) at p3–p4 loci in high‐ and low‐altitude *Rhododendron* species. **(E)** Semi‐quantitative PCR of cDNA at p3–p4 loci in high‐ and low‐altitude *Rhododendron* species.

In addition to these genetic associations, specific amino acid sequence variations were observed. The amino acid sequence of *CPK5*, a regulator of reactive oxygen species (ROS), harbored a histidine insertion in 66.67% of low‐altitude *Rhododendron* species, whereas only 33.3% of high‐altitude species possessed this insertion (Figure S34A). Similarly, the CDS of *FATA* displayed a 17‐amino acid insertion in all nine low‐altitude *Rhododendron* species, whereas this insertion was detected in only five high‐altitude species (Figure S34B). *RFS6* harbored an insertion longer than three amino acids in six low‐altitude samples, whereas this insertion was present in only three high‐altitude samples (Figure S34C). *LPAT2*, which plays a role in phosphatidic acid metabolism, showed a unique genetic pattern among the 18 *Rhododendron* species examined. Relative to the reference genome, eight of the nine high‐altitude species (88.89%) contained an internal insertion of approximately 492 bp within this gene, with the variation particularly evident in *R. nivale*. However, this insertion was only detected in five of the nine low‐altitude species (Figure 5A). This insertion was predominantly observed in *Rhododendron* populations from the QXP, representing almost half of the samples, whereas populations from regions with higher mean annual temperatures displayed markedly fewer instances (Figure 5B). To validate this insertion, primers were designed for PCR amplification. The insertion fragment was successfully amplified in all four high‐altitude species, whereas *R. ovatum* (L) lacked the insertion, with its PCR band being approximately 500 bp shorter than those of the high‐altitude species (Figure 5C). Although the CDS lengths of *LPAT2* were identical between high‐ and low‐altitude species, semi‐quantitative PCR indicated weaker bands in *R. ovatum* (L), implying reduced gene expression potentially attributable to the SV (Figure 5D, E).

Following the analysis of shared climate‐associated variation in *Rhododendron*, attention was directed toward adaptive divergence among species within a single subgenus. *Hymenanthes* species are distributed in high‐ and low‐altitude environments, indicating a high degree of ecological adaptability. This extensive elevational range indicates that populations occupying different altitudes may have experienced varying degrees of genetic differentiation. PCA was conducted on 222 accessions of the subgenus *Hymenanthes*, highlighting two unique genetic clusters corresponding to the QXP and other regions, with a 90% CI (Figure 4B; Dataset S1). On the basis of population structure, samples falling within the CIs of the two clusters were selected for genetic differentiation analysis to detect highly differentiated genomic regions at the single‐nucleotide resolution (*F*_ST_ > 0.31). Figure 4D shows that the outlier variants spanning multiple chromosomes showed well‐defined unimodal patterns. Functional annotation and screening of these candidate regions revealed 17 genes probably involved in plateau environment adaptation. Notably, members of the *CML* (blue text) and *CPK* (red text) gene families were again detected, along with genes such as *UVR8*, *TPPB*, and *LACS4*, which are associated with UV tolerance and cold resistance (Figure 4D). *UVR8* functions as a well‐characterized UV‐B photoreceptor in plants, directly sensing UV stress and acting as a central regulator of UV defense responses, whereas *LIG6* and *KCS1* contribute to DNA repair and cuticular wax biosynthesis, respectively, thereby indirectly improving UV protection (Figure 4E). Based on previous reports of reduced cold tolerance in At5g08350 mutants of *A. thaliana* (Luhua et al., 2013), this gene may also play a role in environmental adaptation. Furthermore, *NCER1* (identified via LFMM analysis) and *sld1* (identified through *F*_ST_ analysis) were detected; both genes are involved in sphingolipid metabolism, a pathway that plays a critical role in environmental stress responses and cellular signaling.

### Differential gene expression contributes to cold resistance in high‐altitude *Rhododendron* species

**Figure 6:**
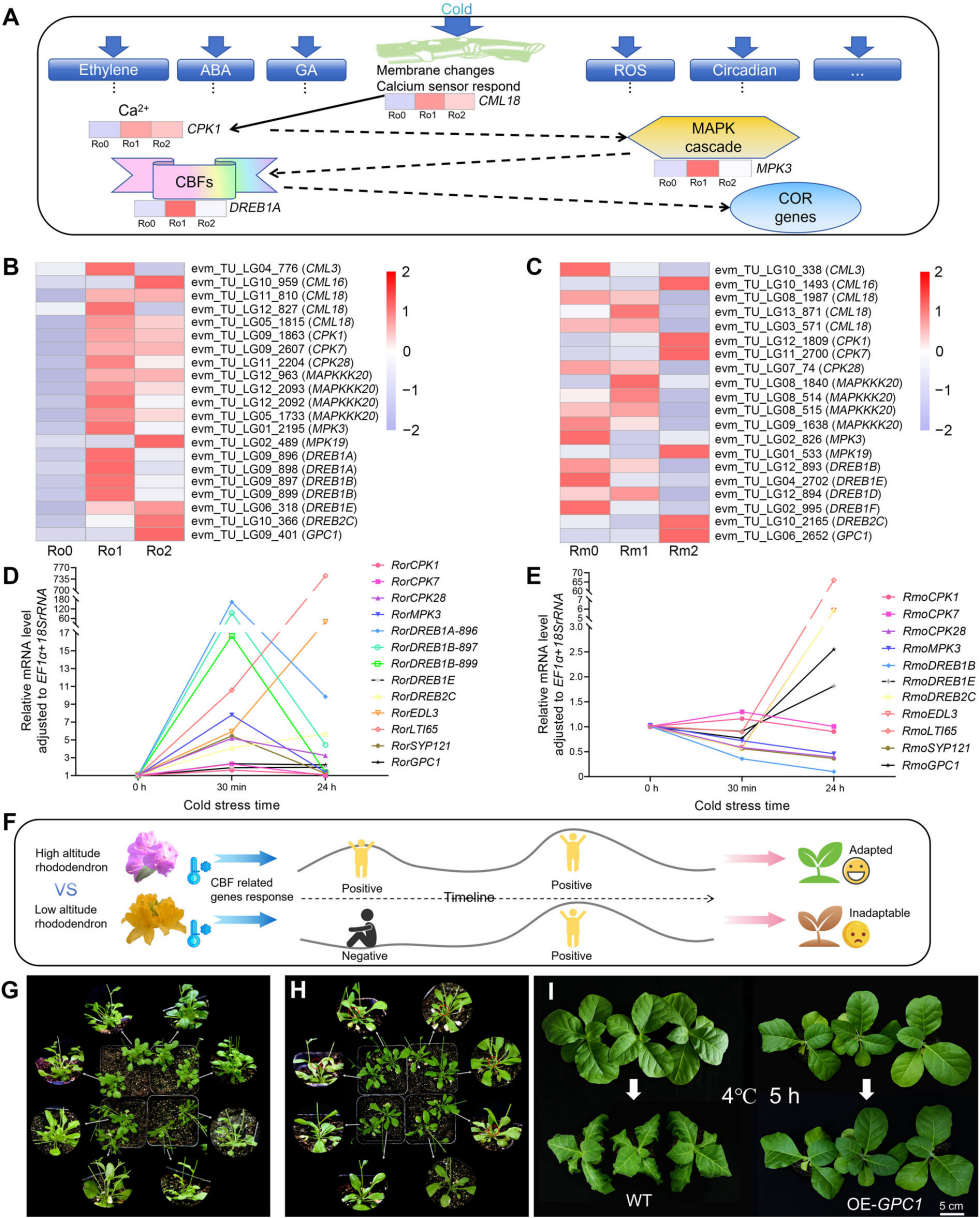
Differences in gene expression under cold‐stress conditions **(A)** Cold‐stress response patterns in *Rhododendron*. **(B**, **C)** Expression patterns of key candidate genes in *R. oreotrephes* (Ro) and *R. molle* (Rm) after 0 h, 0.5 h, and 24 h of low‐temperature exposure. **(D**, **E)** Relative expression levels of cold‐resistance genes in *Rhododendron* species at high **(D)** and low **(E)** altitudes under low‐temperature treatment over time. **(F)** Comparison of cold‐stress response patterns between high‐altitude (*R. oreotrephes*) and low‐altitude (*R. molle*) species. **(G)** Phenotypes of *DREB1E* overexpression T2 generation in *A. thaliana*. **(H)** Wild‐type *A. thaliana* phenotype; red circles indicate decapitation sites. **(I)** Phenotypic changes of wild‐type and *GPC1* overexpression T1 generation flue‐cured tobacco following low‐temperature stress.

Species‐specific responses to the cold plateau environment were investigated to understand why low‐altitude species do not thrive under these conditions. To address these questions, *R. oreotrephes* (high altitude) and *R. molle* (low altitude) were selected for transcriptome analysis. Tender leaves from both species were collected at 0 h, 30 min, and 24 h after exposure to cold treatment at 4°C and labeled as Ro0, Ro1, Ro2, Rm0, Rm1, and Rm2, respectively (see Materials and Methods for details). A higher number of DEGs were observed after 24 h compared with the 30 min cold treatment (Figure S35). Key cold‐responsive genes were identified using GO, KEGG pathway analysis, protein–protein interaction (PPI) networks, and Venn diagrams. The expression patterns of the two *Rhododendron* species were examined across varying cold‐stress durations. At 30 min post‐treatment, *R. oreotrephes* (H) showed significant upregulation of genes associated with potential calcium sensors, the MAPK signaling cascade, ABA signaling, *CBFs/DREB1s*, DNA Repair, transcriptional activators, and *DREB1A* inhibition. Conversely, *R. molle* (L) showed significant induction of genes involved in glutathione metabolism, redox processes, ethylene signaling, GA signaling, cuticular wax biosynthesis, photosynthesis, light perception, and sugar metabolism (Figure S36). After 24 h of cold exposure, most genes upregulated during the early stress response in *R. oreotrephes* (H) remained highly expressed, with significant upregulation of genes involved in GA signaling, calcium‐mediated signal transduction, reactive oxygen species (ROS) metabolism, brassinolide signaling, glutathione and sugar metabolism, cell wall and membrane maintenance, redox regulation, and circadian rhythm (Figure S37). Concurrently, *R. molle* (L) displayed significant upregulation of genes associated with glutathione metabolism, ABA signaling, ROS metabolism, *DREB1A* inhibition, anthocyanin biosynthesis, circadian rhythm, endoplasmic reticulum function, terpenoid metabolism, microtubule dynamics, cytokinin signaling, and osmotic stress responses. The cold‐stress response in *Rhododendron* operates via various interconnected pathways, including membrane lipid sensing, cold signal transduction, CBFs/DREB1s‐mediated transcription, ABA signaling, GA regulation, ethylene‐mediated responses, ROS metabolism, photoprotection mechanisms, and circadian rhythm modulation (Figure 6A).

Remarkably, genes associated with potential calcium sensors, the MAPK signaling cascade, and CBFs/DREB1s in *R. oreotrephes* (H) showed substantially higher activity than those in *R. molle* (L) at both 30 min and 24 h following low‐temperature treatment (Figures S36, S37). Specifically, in *R. oreotrephes* (H), cold perception genes (e.g., *CML3*, *CML16*, and *CML18*), signal transduction genes (e.g., *CPK1*, *CPK7*, *CPK28*, *MAPKKK20*, *MPK3*, and *MPK19*), and stress response genes, including the *DREB1* family, were rapidly upregulated during the initial cold exposure phases and sustained high expression levels throughout the subsequent cold‐stress stages (Figure 6B). In contrast, these genes in *R. molle* (L) displayed little changes or were downregulated, with some genes only becoming significantly upregulated during subsequent cold exposure. Notably, *DREB2C*, chiefly recognized for its function in regulating high‐salinity responses and ABA‐mediated transcription (Sakuma et al., 2002), was markedly upregulated in both species following 24 h of cold treatment (Figure 6C). Furthermore, multiple previously detected genes using LFMM and F‐statistics (*F*_ST_) analyses demonstrated pronounced differential expression in the transcriptome data. These included *KCS4*, *RFS*, *RFS2*, *PAO5*, *STS1*, *NCER1*, *RMA1H1*, *CML16*, and *MPK19* (identified via LFMM) and *CML18*, *CPK1*, *GST3*, and *sld1* (identified through *F*_ST_) (Table S14).

RT‐qPCR was performed to corroborate the transcriptome findings and evaluate the expression of key cold‐sensing and cold‐responsive genes. The findings aligned with the transcriptome data: all examined genes in *R. oreotrephes* (H) were upregulated within 30 min of cold exposure, and most of the highest expression levels were sustained after 24 h (Figure 6D). In *R. molle* (L), these genes demonstrated postponed or attenuated responses during the initial cold‐stress phase, with some being substantially upregulated after 24 h (Figure 6E). The swift and persistent activation of the CBF‐centered pathway likely constitutes a main framework allowing *Rhododendron* species to withstand high‐altitude cold conditions. Conversely, low‐altitude *Rhododendron* species primarily rely on alternative cold‐resistance pathways involving redox regulation, ethylene signaling, and sugar metabolism during the early stages of cold stress. Such responses may be inadequate to confer effective cold resistance, resulting in damage during the early period of cold exposure and compromising long‐term survival (Figure 6F).

### Functional verification of candidate genes

*GPC1*, which is associated with membrane lipid balance, and the cold‐responsive transcription factor *DREB1E* were selected for functional validation. *GPC1* was markedly upregulated in both high‐ and low‐altitude *Rhododendron* species during the later phase of cold stress (Figure 6B–E), although its role in cold resistance has rarely been reported. The *DREB1E* overexpression construct was transformed into Col‐0 wild‐type *Arabidopsis thaliana*. RT‐qPCR analysis demonstrated that *DREB1E* expression was substantially higher in the T2 transgenic Arabidopsis lines than in the wild‐type plants (Figure S38A). The transgenic T2 plants showed retarded growth relative to the wild type; however, no significant difference in biomass (fresh weight) was detected between the two groups (Figures 6G, H, S38C). Nevertheless, the transgenic plants produced a significantly greater number of basal rosette leaves than the wild type (Figures 6G, H, S38D). Moreover, a higher number of stems emerged from the transgenic plants' base. This variation persisted even after decapitation treatment of wild‐type plants, whose basal stem count remained lower than that of the transgenic lines (Figures 6G, H, S38E).

Wild‐type (WT) *Arabidopsis thaliana* and *DREB1E‐*overexpressing (OE) transgenic Arabidopsis plants at the T2 generation were first acclimated at 4°C for 3 d, during which no significant phenotypic differences were observed between the two groups. The plants were subsequently subjected to freezing stress at −6°C for 5 h. Following treatment, both WT and OE plants displayed leaf wilting; however, WT plants showed pronounced stem drooping and lodging, whereas OE plants showed only slight stem softening (Figure S38F). Physiological assessments indicated that OE plants had significantly reduced malondialdehyde (MDA) levels and markedly higher superoxide dismutase (SOD) activity than WT plants (Figure S38G, H). In addition, OE plants showed significantly lower soluble sugar content than WT plants (Figure S38I). Collectively, these physiological responses demonstrate that OE Arabidopsis plants possess enhanced cold tolerance relative to WT plants, indicating that *DREB1E* contributes to improved cold resistance in Arabidopsis.

In addition, an overexpression vector of *GPC1* from *R. oreotrephes* was generated and introduced into the WT flue‐cured tobacco cultivar K326. No significant morphological variations were observed between transgenic T1 tobacco plants and the WT under normal growth conditions. RT‐qPCR confirmed that *GPC1* expression levels were significantly higher in the transgenic T1 plants than those in the WT (Figure S38B). WT tobacco leaves showed wilting after exposure to 4°C for 5 h, whereas no obvious leaf damage was observed in the T1 plants (Figure 6I). Physiological analyses further showed that the MDA content in transgenic leaves was significantly lower than that in WT leaves, whereas SOD activity and soluble sugar content were significantly higher in transgenic plants than those in WT leaves (Figure S38J–L). These findings reveal that *GPC1* from *R. oreotrephes* markedly improves cold tolerance in flue‐cured tobacco K326 and provides a potential strategy for enhancing this cultivar's poor cold resistance.

## DISCUSSION

Genome sizes among *Rhododendron* species display pronounced bipolar differentiation, with smaller genomes arising before the divergence of subgenus *Tsutsusi*, followed by the subsequent emergence of larger genomes. Paleoclimatic evidence based on deep‐sea oxygen isotope (δ^18^O) records indicates that major global cooling events occurred during the Eocene (56–34 Mya) and the Early Oligocene (Zachos et al., 2001), with a reduction in atmospheric oxygen levels. These environmental changes may have facilitated subgeneric divergence and influenced genome size variation within *Rhododendron*. In addition, dynamic changes in gene family expansion and contraction, together with LTR retrotransposon activity, have played important roles in shaping genome architecture. Phylogenetic and ecological analyses further indicated that *R. nivale* and *R. laudandum* have a comparatively short genetic distance and both display an alpine cushion shrub growth form, whereas *R. nivale* and *R. principis* occupy the same slope (Figure S39). Despite this spatial overlap, only *R. nivale* and *R. laudandum* show evidence of a co‐evolutionary trend, indicating that species with tighter phylogenetic affinities and comparable morphologies are more prone to show convergent evolution. The periods during which WGD and WGT events occurred in *Rhododendron* coincided with episodes of global oceanic anoxia and rapid temperature fluctuations (Clarke and Jenkyns, 1999; Dromart et al., 2003; Forster et al., 2007; Korte et al., 2015), highlighting the potential influence of large‐scale environmental perturbations on genome evolution. In this study, *R. ovatum* experienced a greater number of chromosomal rearrangements and had more species‐specific SVs than other *Rhododendron* species examined. Comparative genomic variation analyses demonstrate that this species displays a higher frequency of chromosomal translocations relative to its congeners, indicating that karyotype evolutionary dynamics in *Rhododendron* may create the formation of SVs and be reflected in genome size. No uniform pattern of chromosomal fusion was detected across *Rhododendron* subgenera, indicating that shared fusion events are better suited for resolving phylogenetic relationships at broader taxonomic levels, such as families (Sun et al., 2024).

Genes influenced by distinct LTRs at high and low altitudes display functional parallels with altitude‐specific genes, demonstrating that LTRs not only impact genome size but may also promote adaptive evolution in *Rhododendron* by incorporating environment‐responsive genes. Candidate genes identified using two population genetic analyses functioned in analogous biological pathways. Subsequent validation via transcriptome profiling and RT‐qPCR corroborated these results, revealing that the expression patterns of these genes play a pivotal role in high‐altitude adaptation and constitute a common genetic mechanism underlying the resilience of the species to extreme conditions. Genetic variations within these candidate genes may govern their expression levels, allowing *Rhododendron* species to regulate physiological and developmental responses to environmental stress.

Comparative transcriptome analyses across multiple time points revealed unique low‐temperature response strategies between high‐ and low‐altitude *Rhododendron* species. High‐altitude species promptly perceive cold stress and trigger a robust response pathway centered on *DREB1*, whereas low‐altitude species display delayed cold sensing, with certain *DREB1*‐related genes being downregulated in the early phase. These low‐altitude species predominantly depend on alternative, comparatively weaker cold‐resistance pathways to cope with initial low‐temperature exposure, reflecting a strategy that emphasizes peripheral mechanisms over core resistance functions—an approach likely unsustainable over the long term. Moreover, *DREB1E* overexpression in Arabidopsis produces plants with enhanced tillering and leaf proliferation, yet a compact, dwarf morphology relative to WT *A. thaliana*. Functioning as a “master regulator” within the plant stress‐resistance network, DREB transcription factors coordinate the expression of multiple downstream genes. Continuous stimulation of stress‐protective proteins (e.g., those involved in drought and cold resistance) occurs in Arabidopsis strains overexpressing *DREB1E*, consuming considerable energy and carbon resources. In response to this persistent “stress signal”, plants may redirect resources from elongation growth toward increased tillering and stress‐mitigating compound synthesis, thereby showing a “dwarf and sturdy” phenotype with elevated leaf production. This phenotype is particularly pronounced in *Rhododendron* species inhabiting extremely high altitudes (Figure S39). Soluble sugar levels in *DREB1E*‐overexpressing plants were substantially lower than in WT plants after exposure to freeze stress, which may appear counterintuitive, as soluble sugar accumulation increases osmotic adjustment under cold stress. Nevertheless, plant cold‐tolerance strategies are intricate and multifaceted and are not dependent solely on soluble sugar accumulation. *DREB1E* may preferentially engage alternative, highly effective cold‐resistance pathways independent of soluble sugar levels, including elevated superoxide dismutase (SOD) activity and increased production of late embryogenesis abundant (LEA) proteins and antifreeze proteins (AFPs). As previously indicated, the continuous biosynthesis of large quantities of protective molecules is energetically demanding. As vital carbon reserves and energy sources, soluble sugars may be reallocated toward the synthesis of these essential protective compounds rather than being retained as free sugars.

The variability of samples sourced from distinct *Rhododendron* germplasm collections is an inherent limitation of this study. Nevertheless, integrative multiomics and multimodel analyses produced coherent results, supporting the identification of shared adaptive traits among diverse accessions. Previous research provides a valuable context for such comparable analyses: Genome‐wide association studies (GWAS) in various *Rhododendron* germplasms have identified genes associated with bark roughness (Ye et al., 2024), whereas LFMM analyses of 153 accessions from 17 *Solanum* species have uncovered the genetic underpinnings of environmental adaptation within this genus in Africa (Omondi et al., 2024). These studies serve as useful references for examining common high‐altitude adaptation mechanisms across *Rhododendron* species. Future research should broaden population sampling within and among *Rhododendron* species to further elucidate shared and species‐specific adaptive traits in response to environmental variables such as temperature, humidity, and soil pH.

In summary, this study systematically explored the evolutionary history of *Rhododendron* and uncovered genetic convergencies underlying adaptation to high‐altitude habitats, including specific metabolic pathways and gene families. Throughout the divergence of *Rhododendron* differentiation, LTRs and gene family expansion or contraction modulated genetic inheritance patterns. The pangenome framework offers a holistic and interpretable strategy for examining these genomic dynamics. Under the Qinghai–Xizang Plateau's extreme low‐temperature and high‐UV conditions, high‐altitude *Rhododendron* species have evolved distinct adaptive responses. Enhanced cold sensing has enabled the prompt activation of the principal cold‐stress pathways, thereby mitigating environmental damage. In response to intense UV radiation, genes such as *UVR8*, *LIG6*, and *KCS1* directly or indirectly elicit protective responses. Collectively, this study broadens the framework for understanding *Rhododendron* resilience and provides critical insights for phylogenetic interpretation and molecular breeding strategies within the genus.

## MATERIALS AND METHODS

### Material collection, sequencing, and genome estimation

Seven accessions were sampled from Nyingchi (Xizang), Jiujiang (Jiangxi Province), and Anqing (Anhui Province). Genomic DNA was extracted for subsequent next‐generation sequencing using the sodium dodecyl sulfate–protease K method (Bi et al., 2021). A short‐insert DNA library (200–300 bp) was constructed, and paired‐end high‐throughput sequencing was performed on the DNBSEQ‐T7 platform following the whole‐genome shotgun strategy. Raw reads were filtered with fastp v.0.19.4 (Chen et al., 2018a) using the parameters − n 0 − f 5 − F 5 − t 5 − T 5, and the quality of the resulting clean reads was assessed with FastQC (http://www.bioinformatics.babraham.ac.uk/projects/fastqc). The *K*‐mer frequencies of the filtered reads were calculated using Jellyfish v.2.3.0 (Marçais and Kingsford, 2011). The k‐mer distributions were fitted to the normal and negative binomial skew models. Genome size and heterozygosity were estimated according to the respective models using findGSE (Sun et al., 2018) and GenomeScope v.1.1.1 (Vurture et al., 2017), and the final genome metrics were consolidated.

High‐molecular‐weight DNA was extracted using QIAGEN® Genomic‐tip kits to construct SMRTbell libraries of the target size for long‐read sequencing. The library quality was evaluated using SMRTlink (PacBio), and low‐quality regions were filtered based on the signal‐to‐noise ratio thresholds. Circular consensus sequencing (CCS) reads were generated and processed using CCS (https://github.com/PacificBiosciences/ccs) with the following settings: ‐‐ min rq 0.99 ‐‐ min length 100.

### Genome assembly

The initial genome assembly was generated using the default parameters of Hifiasm v.0.16 (https://github.com/chhylp123/hifiasm). PacBio contigs were polished four times with Nextpolish v.1.4.0 (Hu et al., 2020) using quality‐controlled Next‐Generation Sequencing reads to improve assembly accuracy. BUSCO v.5.0 (Simão et al., 2015) was used to assess gene completeness based on single‐copy orthologs in the OrthoDB embryophyta_odb10 data set, thereby evaluating the integrity of the assembled genomes. To further validate assembly accuracy, NGS and PacBio reads were aligned to the polished genome using BWA v.0.7.17‐r1188 (Li and Durbin, 2010) and minimap2 v.2.22 (Li, 2016).

Hi‐C (Lieberman‐Aiden et al., 2009) was performed to facilitate scaffolding and achieve chromosome‐level assemblies. Contigs were clustered, ordered using a bottom‐up hierarchical clustering algorithm, and oriented into chromosomes with *LACHESIS* (https://github.com/shendurelab/LACHESIS) using the following parameters: CLUSTER MIN RE SITES = 100; CLUSTER MAX LINK DENSITY = 2.5; CLUSTER NONINFORMATIVE RATIO = 1.4; ORDER MIN N RES IN TRUNK = 60; and ORDER MIN N RES IN SHREDS = 60. The draft contigs were grouped into 13 chromosome clusters and subsequently ordered within each cluster based on the results. A Hi‐C contact heatmap with 100‐kb bins representing genomic intervals was generated using Python. The number of Hi‐C read pairs connecting any two bins was used to quantify the interaction intensity between them. Chromosomal coordinates are represented on the axes, and the color intensity of each point reflects the logarithmic value of the interaction frequency between corresponding bin pairs. BUSCO (v. 5.2.1) was used to evaluate the completeness of the chromosome‐level genome assembly.

### Genome annotation and gene clustering

Tandem repeats (TRs) containing simple sequence repeats were identified and characterized using GMATA v.2.2 (Wang and Wang, 2016) with default settings and TRF v.4.07b (Benson, 1999). To investigate the relationship between TEs, other repetitive sequences, and genome size in *Rhododendron*, repetitive sequences in 18 *Rhododendron* genome assemblies were re‐annotated using a homology‐based repeat library constructed from Repbase (Jurka et al., 2005) and a *de novo* repeat library generated with RepeatModeler (Bedell et al., 2000). Repeat elements were subsequently annotated using RepeatMasker (v.1.331).

Gene structure was predicted using ab initio, and homology‐ and transcriptome‐based approaches. The results were integrated using EvidenceModeler (EVM) v.1.1.1 (Haas et al., 2008) to generate an initial set of predicted genes. Genes containing TEs were filtered using TransposonPSI (Urasaki et al., 2017) with default parameters to obtain the final annotated gene set. The functional annotation of these genes was performed against the KEGG, Eukaryotic Orthologous Groups of Protein, GO, Swissprot, and Trembl databases.

Infernal v.1.1.2 (Nawrocki and Eddy, 2013) was used to align the genome against the Rfam database (Griffiths‐Jones et al., 2005) to identify microRNAs and small nuclear RNAs. tRNAscan‐SE v.2.0 (Lowe and Eddy, 1997) and RNAmer v.1.2 (Lagesen et al., 2007) were used to predict tRNAs and rRNAs, respectively.

Gene families from 17 accessions and *R. vialii* were clustered using OrthoMCL v.2.0.9 (Li et al., 2003). Homologous proteins were identified using BLASTp (Camacho et al., 2009) with an E‐value cutoff of 1e−5. The resulting similarity data were used to cluster gene families in OrthoMCL with parameters percent Match Cutoff = 50 and − I 1.5. GO terms were inferred from SwissProt and Trembl, and KEGG pathway enrichment was analyzed using KOBAS v.3.0 (Xie et al., 2011). Enrichment significance was calculated using the R package phyper, and the *P*‐values were adjusted.

### Phylogenetic analysis

For the resequencing data, SNPs were used to construct a maximum likelihood (ML) phylogenetic tree with RAxML‐NG v.1.1.0 (Stamatakis, 2006), using the parameters ‐‐model GTR + G4 and ‐‐ bs ‐ trees 1000. Two species of the subgenus *Therorhodion* (RH001 and RH990) were designated as outgroups. The optimal nucleotide substitution model was selected using ModelTest‐NG v.0.1.7.

Genome assemblies were collected from 32 angiosperm species for comparative genomic analyses. Gene families were identified using OrthoMCL v.2.0.9, yielding 490 LCN orthologs shared among these 32 species. LCN orthologs were defined as genes present as a single copy in each of the 31 genomes and with at least one copy in *Vaccinium corymbosum*. The protein‐coding sequences were aligned using MAFFT v.7.490 software. Then, an ML phylogenetic tree was reconstructed with RAxML using the optimal amino acid substitution model (‐m PROTGAMMAIJTTF).

Divergence time estimation was performed using the MCMCTree module in PAML v.4.9 h (Yang, 2007) with six calibration constraints: (i) the crown node of Ericales (89.8 Mya) (Nixon and Crepet, 1993); (ii) the stem node of *Rhododendron* (56 Mya) (Collinson and Crane, 2010); (iii) Angiosperms (199–167 Mya); (iv) asterids–rosids (126–116 Mya) (Li et al., 2019); and two additional calibration points obtained from the TimeTree database (*Phalaenopsis equestris*–*Oryza sativa*, 123.6–108 Mya; *Arabidopsis thaliana*–*Prunus mira*, 112.5–102 Mya). CAFÉ (De Bie et al., 2006) was used to identify expanded and contracted gene families based on the time‐calibrated phylogeny. Subsequently, these gene families were mapped to GO terms and KEGG pathways. Functional enrichment analyses were conducted using the R package phyper, with *P*‐values adjusted for multiple testing; GO terms and KEGG pathways with adjusted *P* value < 0.05 were considered significantly enriched.

### Whole‐genome duplication (WGD) and chromosome evolution

The whole‐genome duplication identifier (WGDI) pipeline was used to investigate WGD events and chromosomal evolutionary patterns (Sun et al., 2022). The inferred ancestral karyotype of core eudicots generated by WGDI was used as the reference framework for subsequent analyses. Protein sequences from each accession were aligned to the ancestral karyotype using BLAST with default parameters. Chromosomal evolutionary trajectories were inferred from the resulting syntenic scatter plots, with the most parsimonious scenario requiring the minimum number of chromosomal fusion events.

### Genomic variants' identification and graph‐based pangenome construction

SNPs and small Indels were identified at the genomic scale using the show‐snps module (‐ClrT) of the MUMmer4 toolkit. PAVs were detected based on Indels identified by the SV module of the MUMer‐based SVMU pipeline (Chakraborty et al., 2019). Genome regions that were neither classified as syntenic blocks by MUMmer nor identified as SVs by the SVMU pipeline were designated as candidate PAV regions. Regions with multiple alignments in MUMmer were considered candidate CNVs after excluding short segments < 100 bp in size.

The SVMU pipeline was further applied to identify chromosomal translocations and inversions based on the positional relationships and orientations of neighboring syntenic blocks. PAVs were subsequently merged following criteria adapted from previous soybean studies (Liu et al., 2020): (i) PAVs of the same type were merged based on overlapping genomic intervals for single accessions; (ii) PAVs identified in the previous step were then merged sequentially across accessions; and (iii) the reference genome of *R. vialii*, together with other nonredundant SVs present in < 90% of the 13 genomes and associated with repetitive sequences, was incorporated into a variant graph using *vg* (Garrison et al., 2018) without excluding alternative alleles.

Minimap2 v.2‐2.24 was used to align the query genomes to the reference genome using default parameters. Structural variants were identified using syri v.1.7.1, and the resulting variation patterns were visualized using the appendant tool “plotsr”.

### Processing of the resequenced data

For resequencing, high‐quality genomic DNA was isolated from 101 samples using a modified CTAB protocol (Porebski et al., 1997). DNA concentrations were quantified using the Qubit v.4.0 fluorometer, and DNA purity and integrity were assessed using the A_260/280_ absorbance ratio and 1% agarose gel electrophoresis, respectively. Qualified samples were sequenced on the DNBSEQ‐T7 platform, achieving an average genome coverage depth of 49.1×, calculated based on the genome size of *R. vialii* 532.73 Mb. Additional resequencing data sets were retrieved from the National Center of Biotechnology Information (NCBI) and the National Genomics Data Center (NGDC), with detailed information provided in Dataset S9. The mean sequencing depth across all resequenced samples was 50.2×.

To identify SNPs and Indels (< 50 bp) from the resequencing data of 389 samples, sequencing reads were aligned to the reference genome using BWA v.0.7.17 with default settings. Duplicate reads were marked using SAMtools v.1.4. Variant calling was performed using GATK v.4.1.2.0 in the Genomic variant call format (GVCF) mode (‐ERC GVCF). Low‐confidence variant sites were filtered using VCFtools v.0.1.16 with the following criteria: ‐‐min‐alleles 2, ‐‐max‐alleles 2, ‐‐min‐meanDP 5, ‐‐maf 0.05, and ‐‐max‐missing 0.8. Subsequently, raw SNPs and Indels were further filtered using GATK with a sliding‐window approach (–cluster 3 –window 10). SNPs were filtered using the expression “QUAL < 30.0 ‖ QD < 2.0 ‖ FS > 60.0 ‖ SOR > 4.0” (‐‐filter‐name snp_filter), whereas Indels were filtered using “QUAL < 30.0 ‖ QD < 2.0 ‖ FS > 200.0 ‖ SOR > 10.0” (‐‐filter‐name indel_filter).

### LTR analysis

LTR retrotransposons were identified using LTR_FINDER v.1.07 and LTR_retriever v.2.9.0. LTR_FINDER was executed with the following parameters: –D 15000 –d 1000 –L 7000 –l 100 –p 20 –C –M 0.9, while the default settings were applied to LTR_retriever. The density of LTR insertion times was visualized using the ggplot2 package in R and extracted from the LTR_retriever output.

LTRs from accessions DJ3 (*R. principis*) and *R. fortunei* (both belonging to subgenus *Hymenanthes*) were selected to represent the high‐ and low‐altitude LTR elements, respectively, based on the repeat annotation results described above. Pairwise all‐against‐all BLASTN alignments were performed between the LTR sequences from the two accessions using an E‐value threshold of 1e−5. LTRs from *R. principis* that did not yield significant matches with *R. fortunei* LTRs were classified as high‐altitude‐specific LTRs, and vice versa. An analogous procedure was applied to *R. molle* (characterized by a large genome) and *R. simsii* (characterized by a small genome), both of which are distributed at low altitudes. Altogether, 46,005 and 97,664 LTRs were classified as high‐altitude‐specific and large‐genome‐specific, respectively. Genes located within unique LTRs or within 1 kb downstream of these LTRs were extracted as candidate genes using in‐house Perl scripts. Functional enrichment analysis based on KEGG pathways was subsequently conducted following the procedures described above.

### Screening of specific gene families

Global occurrence records for 18 *Rhododendron* species were obtained from the global biodiversity information facility (GBIF). The initial data filtering involved removing entries with missing latitude or longitude values and eliminating duplicate records. The data set was further refined using CoordinateCleaner v.3.0.1 to improve geospatial accuracy. Records corresponding to introduced or cultivated populations were excluded using rWCVP v.1.2.4, yielding the final set of natural distribution points. These points were visualized using ArcMap v.10.8. Elevation data were retrieved from the WorldClim database at a resolution of 2.5 arcmin. The corresponding altitudes were extracted using ArcMap by integrating the cleaned coordinate points, including records from introduced and cultivated areas to expand the observational range with the elevation data set. Subsequently, global elevation distribution statistics for the 18 species were generated by combining these extracted elevations with the specimen‐based elevation information from the National Plant Specimen Resource Center.

Gene families unique to high‐ or low‐altitude groups were identified using the following criterion: A gene family was considered unique to a group if it was present in at least one accession of that group and entirely absent in all accessions of the other group.

### Identification of regional climate adaptation variation sites

The genetic structure of the population was analyzed using ADMIXTURE v.1.3.0 to determine the optimal number of genetic clusters (*K*). Environmental data for 19 bioclimatic variables related to temperature and precipitation (resolution: 2.5 arcmin) were obtained from the WorldClim website (https://www.worldclim.org/data/bioclim.html) (Fick and Hijmans, 2017). The relative importance of climatic factors was assessed using the R package “gradientForest”. Associations between genetic variants and environmental variables were evaluated using LFMMs implemented in the R package “LEA” (Frichot and Francois, 2015). Significance thresholds were determined using a false discovery rate (FDR) approach (blue dashed line), while a more stringent cutoff was provided by the Bonferroni correction (0.05/N) (red dashed line). The frequency distribution of candidate variants was visualized by analyzing the mutation frequencies of SNP and SV sites within the resequencing population. The spatial visualization and mapping of these patterns were performed using ArcMap v.10.8.

### Analysis of selection signatures for high‐altitude adaptation

The population structure was evaluated by performing a PCA using PLINK (Chang et al., 2015) and R. The first two principal components derived from a PCA of 222 samples from the subgenus *Hymenanthes* were used as covariates to account for population structure. Genome‐wide genetic differentiation (*F*_ST_) was calculated using a sliding‐window approach with VCFtools (Danecek et al., 2011), using the following parameters: ‐‐fst‐window‐size 50000 ‐‐fst‐window‐step 10000.

### Transcriptome sequencing and RT‐qPCR

Total RNA was isolated from seedling leaves using the HiPure Plant RNA Mini Kit. Reverse transcription was performed using the HiScript® II Q RT SuperMix for RT‐qPCR (+gDNA wiper). RT‐qPCR was conducted using the PerfectStart® Green qPCR SuperMix on an ABI QuantStudio 7 Flex system. To investigate the similarities and differences in low‐temperature stress responses between high‐ and low‐altitude *Rhododendron* species, *R. oreotrephes* (high altitude) and *R. molle* (low altitude) seedlings were selected. Uniform seedlings were subjected to either low‐temperature treatment (4°C) or normal conditions once they reached an appropriate developmental stage. Leaf samples were collected at 0 h, 15 min, 30 min, 1 h, 2 h, 6 h, 12 h, 24 h, and 48 h, with three biological replicates per time point (each replicate comprising an equal number of seedlings). Subsequently, RNA was extracted from all collected leaf samples for downstream analyses. Reference genes were screened for RT‐qPCR in two *Rhododendron* species. The housekeeping genes *EF1α* and *18srRNA* were selected as the optimal reference gene combination because they showed stable expression under both normal and low‐temperature conditions in both species. RT‐qPCR analyses were conducted for the cold‐resistance‐related genes *GPC1*, *DREB1*, and the adjacent gene *AAA‐ATPase* across nine time points under both treatment conditions. The results indicated significant expression changes at 0 h, 30 min, and 24 h following low‐temperature exposure in both high‐ and low‐altitude *Rhododendron* species, with clear interspecific differences observed (Figure S40). Based on these preliminary findings, leaf tissues from the two species were collected at three selected time points under low‐temperature conditions for transcriptome sequencing. After quality control, RNA samples were used to construct cDNA libraries, which were subsequently sequenced on the Illumina platform to generate 150‐bp paired‐end reads. Raw sequencing data were filtered using the Fastp software to obtain high‐quality clean reads, which were then aligned to the reference genome. Gene expression levels were quantified using featureCounts (v.1.5.0‐p3), and differential expression analysis was performed using DESeq. 2 (v.1.20.0). Functional enrichment analyses for GO terms and KEGG pathways were performed using clusterProfiler (v.3.8.1). PPI networks of the DEGs were constructed using DIAMOND (v.0.9.13) based on the STRING database. Table S15 lists the primers used for RT‐qPCR, and the relative gene expression levels were calculated using the 2^−∆∆CT^ method.

### Identification of the gene function

To validate the intronic structural variation within the LPAT2 gene, PCR amplification was performed using p1/p2 primers targeting the SV region. The coding sequence length of *LPAT2* was verified using p3/p4 primers (Figure 5D shows the agarose gel electrophoresis image of PCR‐amplified and sequentially spotted *LPAT2* coding sequences from various species following cloning). Semi‐quantitative analysis was also conducted using the p3/p4 primers. The *DREB1E* gene from *R. oreotrephes* (H) was cloned into an overexpression vector and introduced into WT *A. thaliana* via *Agrobacterium*‐mediated floral dip transformation, generating T0 transgenic plants. Transformed seeds were sown, and positive seedlings were screened to obtain the T1 generation. Homozygous positive T2 Arabidopsis lines were established through successive rounds of selection for phenotypic characterization and RT‐qPCR analysis. For cold‐stress assays, WT and *DREB1E*‐overexpressing T2 Arabidopsis plants were acclimated at 4°C for 3 d, subsequently exposed to −6°C for 5 h, allowed to thaw for 10 min, and then assessed for morphological changes. Plant tissues were collected and stored at −80°C for subsequent analyses. Physiological parameters, including MDA content, SOD activity, and soluble sugar content, were quantified using commercially available assay kits. For functional validation of the *GPC1* gene from *R. oreotrephes*, an overexpression construct was generated and introduced into the WT flue‐cured tobacco cultivar K326 using *Agrobacterium*‐mediated leaf disc transformation. Transformed leaf explants were cultured to obtain T0 seedlings. Following genomic DNA extraction and semi‐quantitative validation, plants showing high *GPC1* expression were selected for seed production. Subsequently, T1 seedlings were screened, and positive lines were evaluated for phenotypic variations and subjected to RT‐qPCR analysis. WT and *GPC1*‐overexpressing T1‐K326 plants were then exposed to 4°C for 5 h, after which morphological traits were recorded and samples were collected. Cold‐resistance‐related physiological indices were measured using the aforementioned assay kits.

## CONFLICTS OF INTEREST

The authors have no conflicts of interest.

## AUTHOR CONTRIBUTIONS

X.L., S.J., and H.Z. designed the study. X.L. and S.J. provided funding for the research. H.Z. performed most of the research and was responsible for drafting and revising the manuscript. X.L., H.Z., F.Z., H.S., Q.F., and K.Z. conducted field surveys and sampling. Z.X., F.Z., and H.S. participated in the cold treatment pre‐experiments on rhododendrons. Z.X. and Z.L. provided the pan‐genome construction platform and data storage space. M.S. offered code support for the global distribution map of rhododendrons. M.B. and D.T. were involved in revising the manuscript. X.L. and S.J. supervised the research progress and participated in manuscript revision. All authors have read and approved the contents of this paper.

## Supporting information

Additional Supporting Information may be found online in the supporting information tab for this article: http://onlinelibrary.wiley.com/doi/10.1111/jipb.70252/suppinfo

**Dataset S1.** Re‐sequencing accessions information used in this study

**Dataset S2.** GO enrichment information of core and dispensable gene families

**Dataset S3.** KEGG enrichment information of core and private gene families

**Dataset S4.** Global distribution information of 18 species of rhododendron

**Dataset S5.** GO enrichment information of unique genes of *Rhododendron* species at high and low altitudes

**Dataset S6.** KEGG enrichment information of unique genes of *Rhododendron* species at high and low altitudes

**Dataset S7.** KEGG enrichment information of genes affected by SVs

**Dataset S8.** KEGG enrichment analysis of genes influenced by unique LTRs from *R*. *simsii*, *R*. *molle*, *R*. *principis*, and *R*. *fortunei*

**Dataset S9.** Download link for the public resequencing data used in this study

**Figure S1.** Overview of the six rhododendron genome assemblies
**Figure S2.** Genome‐wide all‐by‐all interaction maps of rhododendrons at 100‐kb resolution
**Figure S3.** Global distribution of the native regions of 18 *Rhododendron* species
**Figure S4.** Phylogenetic tree based on 490 orthologs from 32 species
**Figure S5.** KEGG enrichment analysis of contracted gene families in subgenus *Tsutsusi*
**Figure S6.** KEGG enrichment analysis of expanded and contracted gene families in *R. nivale* and *R. laudandum*
**Figure S7.** KEGG pathway enrichment distribution of expanded and contraction gene families in *R. principis*
**Figure S8.** Whole‐genome duplication (WGD) events of *Rhododendron* species
**Figure S9.** Syntenic blocks of *R. nivale*, *R. principis*, *R. laudandum*, *R. oreotrephes*, and *R. latoucheae* genomes
**Figure S10.** Syntenic blocks of *R. fortunei*, *R. molle*, *R. simsii*, and *R. vialii* genomes
**Figure S11.** Homologous blocks between rhododendrons and kiwifruit and grape genomes
**Figure S12.** Genomic comparison between ACEK and *R. nivale*/*R. principis* based on dotplot
**Figure S13.** Genomic comparison between ACEK and *R. laudandum*/*R. oreotrephes* based on dotplot
**Figure S14.** Genomic comparison between ACEK and *R. latoucheae*/*R. fortunei* based on dotplot
**Figure S15.** Genomic comparison between ACEK and *R. molle*/*R. bailiense* based on dotplot
**Figure S16.** Genomic comparison between ACEK and *R. ripense*/*R. simsii* based on dotplot
**Figure S17.** Genomic comparison between ACEK and *R. vialii*/*R. williamsianum* based on dotplot
**Figure S18.** Genomic comparison between ACEK and *R. henanense* subsp. *lingbaoense*/*R. irroratum* based on dotplot
**Figure S19.** Genomic comparison between ACEK and *R. ovatum*/*R. prattii* based on dotplot
**Figure S20.** Genomic comparison between ACEK and *R. delavayi*/*R. griersonianum* based on dotplot
**Figure S21.** Derivation of karyotype evolution of *R. latoucheae*
**Figure S22.** Global altitude distribution statistics of 18 *Rhododendron* species, including records of cultivated introductions
**Figure S23.** KEGG pathway enrichment distribution of the unique‐gene families of rhododendrons at different altitudes
**Figure S24.** Homology and rearrangement of *R. nivale*, *R. principis*, *R. laudandum*, *R. oreotrephes*, *R. latoucheae*, and *R. fortunei* genome using *R. vialii* as the reference
**Figure S25.** Homology and rearrangement of *R. molle*, *R. bailiense*, *R. delavayi*, *R. ovatum*, *R. prattii*, and *R. ripense* genome using *R. vialii* as the reference
**Figure S26.** Homology and rearrangement of *R. griersonianum*, *R. henanense* subsp. *lingbaoense*, *R. irroratum*, *R. simsii*, and *R. williamsianum* genome using *R. vialii* as the reference
**Figure S27.** Insertion time of LTRs in 18 *Rhododendron* species
**Figure S28.** KEGG pathway enrichment distribution of genes in the 1k downstream region of the unique LTRs of two rhododendrons
**Figure S29.** KEGG pathway enrichment distribution of genes in the 1k downstream region of the unique LTRs of two rhododendrons
**Figure S30.** Cross‐validation (CV) error curves (A) and the order of importance of climatic factors (B)
**Figure S31.** Cumulative importance of allele changes on 19 environmental gradients
**Figure S32.** Manhattan plot of LFMM for variants associated with BIO5 (A) and BIO11 (B)
**Figure S33.** Allele frequencies of candidate adaptive SNPs associated with BIO1 across the Qinghai–Tibet Plateau and other populations
**Figure S34.** Mutation status of candidate gene insertions or deletions
**Figure S35.** Volcano plot of differentially expressed genes in *R. oreotrephes* and *R. molle* after 0.5 and 24 h of low‐temperature stress
**Figure S36.** Changes in the expression levels of cold resistance‐related genes in *R. oreotrephes* (high‐altitude) and *R. molle* (low‐altitude) under 4°C low‐temperature treatment for 30 min
**Figure S37.** Changes in the expression levels of cold resistance‐related genes in *R. oreotrephes* (high‐altitude) and *R. molle* (low‐altitude) under 4°C low‐temperature treatment for 24 h
**Figure S38.** Functional validation of cold resistance candidate genes
**Figure S39.** Ecological environments of the high‐altitude rhododendron community in Tibet
**Figure S40.** qPCR analysis of *Rhododendron* species at different altitudes
**Table S1.** Information of genome MGI sequencing
**Table S2.** Information of genome PacBio sequencing
**Table S3.** Information of Hi‐C auxiliary assembly
**Table S4.** Benchmarking universal single‐copy orthologs of genome HiC assembly and annotation
**Table S5.** The predicted coding protein gene information of in genome
**Table S6.** Information of genomic transposable elements
**Table S7.** Information of genomic non‐coding RNAs
**Table S8.** Information of pan‐genome gene family clustering
**Table S9.** Summary of identified insertions, presence and absence variation
**Table S10.** Summary of identified intra‐chromosomal and inter‐chromosomal translocations
**Table S11.** Summary of identified copy number variants
**Table S12.** Information of SVs as the number of samples changes
**Table S13.** Information on SVs classification of different rhododendrons
**Table S14.** Significant gene information screened by different analysis
**Table S15.** The primers used in the PCR

## Data Availability

All data supporting the findings of this study are available in the main text and supplementary information. The whole‐genome raw sequencing data, transcriptome raw sequencing data, and genome assemblies generated in this study have been deposited at the Genome Sequence Archive and the Genome Warehouse of the National Genomics Data Center (NGDC), China National Center for Bioinformation, Chinese Academy of Sciences, under BioProject ID PRJCA021036. The corresponding accession numbers are CRA013427, CRA013492, CRA041670, GWHEQCY00000000, GWHEQCZ00000000, GWHEQDA00000000, GWHEQDB00000000, GWHEQDC00000000, GWHEQDD00000000, and GWHEQDE00000000. These data sets are publicly accessible on the NGDC platform at https://ngdc.cncb.ac.cn.
