# Supplementary material for: Pangenome analysis reveals the genetic mechanism underlying high‐altitude adaptation in Qinghai–Xizang (Tibet) Plateau Rhododendron
Source: J Integr Plant Biol. 2026 Apr 12;68(7):2166–88. doi: 10.1111/jipb.70252 (PMC13326988; doi:10.1111/jipb.70252)
Supplement: Supplementary file 5 — Dataset S5. GO enrichment information of unique genes of Rhododendron species at high and low altitudes [file JIPB-68-2166-s002.docx]

**Dataset S5.** KEGG enrichment analysis of genes influenced by unique LTRs from *R. simsii*, *R. molle*, *R. principis*, and *R. fortunei*
