# Supplementary material for: Pangenome analysis reveals the genetic mechanism underlying high‐altitude adaptation in Qinghai–Xizang (Tibet) Plateau Rhododendron
Source: J Integr Plant Biol. 2026 Apr 12;68(7):2166–88. doi: 10.1111/jipb.70252 (PMC13326988; doi:10.1111/jipb.70252)
Supplement: Supplementary file 10 — Figure S1. Overview of the six rhododendron genome assemblies Figure S2. Genome‐wide all‐by‐all interaction maps of rhododendrons at 100‐kb resolution Figure S3. Global distribution of the native regions of 18 Rhododendron species Figure S4. Phylogenetic tree based on 490 orthologs from 32 species Figure S5. KEGG enrichment analysis of contracted gene families in subgenus Tsutsusi Figure S6. KEGG enrichment analysis of expanded and contracted gene families in R. nivale and R. laudandum Figure S7. KEGG pathway enrichment distribution of expanded and contraction gene families in R. principis Figure S8. Whole‐genome duplication (WGD) events of Rhododendron species Figure S9. Syntenic blocks of R. nivale, R. principis, R. laudandum, R. oreotrephes, and R. latoucheae genomes Figure S10. Syntenic blocks of R. fortunei, R. molle, R. simsii, and R. vialii genomes Figure S11. Homologous blocks between rhododendrons and kiwifruit and grape genomes Figure S12. Genomic comparison between ACEK and R. nivale/R. principis based on dotplot Figure S13. Genomic comparison between ACEK and R. laudandum/R. oreotrephes based on dotplot Figure S14. Genomic comparison between ACEK and R. latoucheae/R. fortunei based on dotplot Figure S15. Genomic comparison between ACEK and R. molle/R. bailiense based on dotplot Figure S16. Genomic comparison between ACEK and R. ripense/R. simsii based on dotplot Figure S17. Genomic comparison between ACEK and R. vialii/R. williamsianum based on dotplot Figure S18. Genomic comparison between ACEK and R. henanense subsp. lingbaoense/R. irroratum based on dotplot Figure S19. Genomic comparison between ACEK and R. ovatum/R. prattii based on dotplot Figure S20. Genomic comparison between ACEK and R. delavayi/R. griersonianum based on dotplot Figure S21. Derivation of karyotype evolution of R. latoucheae Figure S22. Global altitude distribution statistics of 18 Rhododendron species, including records of cultivated introductions Figure S23. KEGG pathway enrichm [file JIPB-68-2166-s004.docx]

**SUPPORTING INFORMATION**

Pangenome analysis reveals genetic mechanism underlying high-altitude adaptation in Qinghai-Xizang (Tibet) Plateau *Rhododendron*

Haoyang Zhou, Zhongping Xu, Fanhuang Zeng, Haiyu Sang, Zhenhua Liu, Miao Sun, Qiang Fu, Kaige Zhao, Daming Tan, Manzhu Bao, Shuangxia Jin and Xiuqun Liu

*Correspondence: Shuangxia Jin [(jsx@mail.hzau.edu.cn);](mailto:(jsx@mail.hzau.edu.cn);) Xiuqun Liu (liuxiuqun@mail.hzau.edu.cn, Dr. Liu is fully responsible for the distribution of all materials associated with this article)

**This PDF file includes:**

**SUPPORTING FIGURES S1 TO S40**

**SUPPORTING TABLES S1 TO S14**

**SUPPORTING FIGURES**


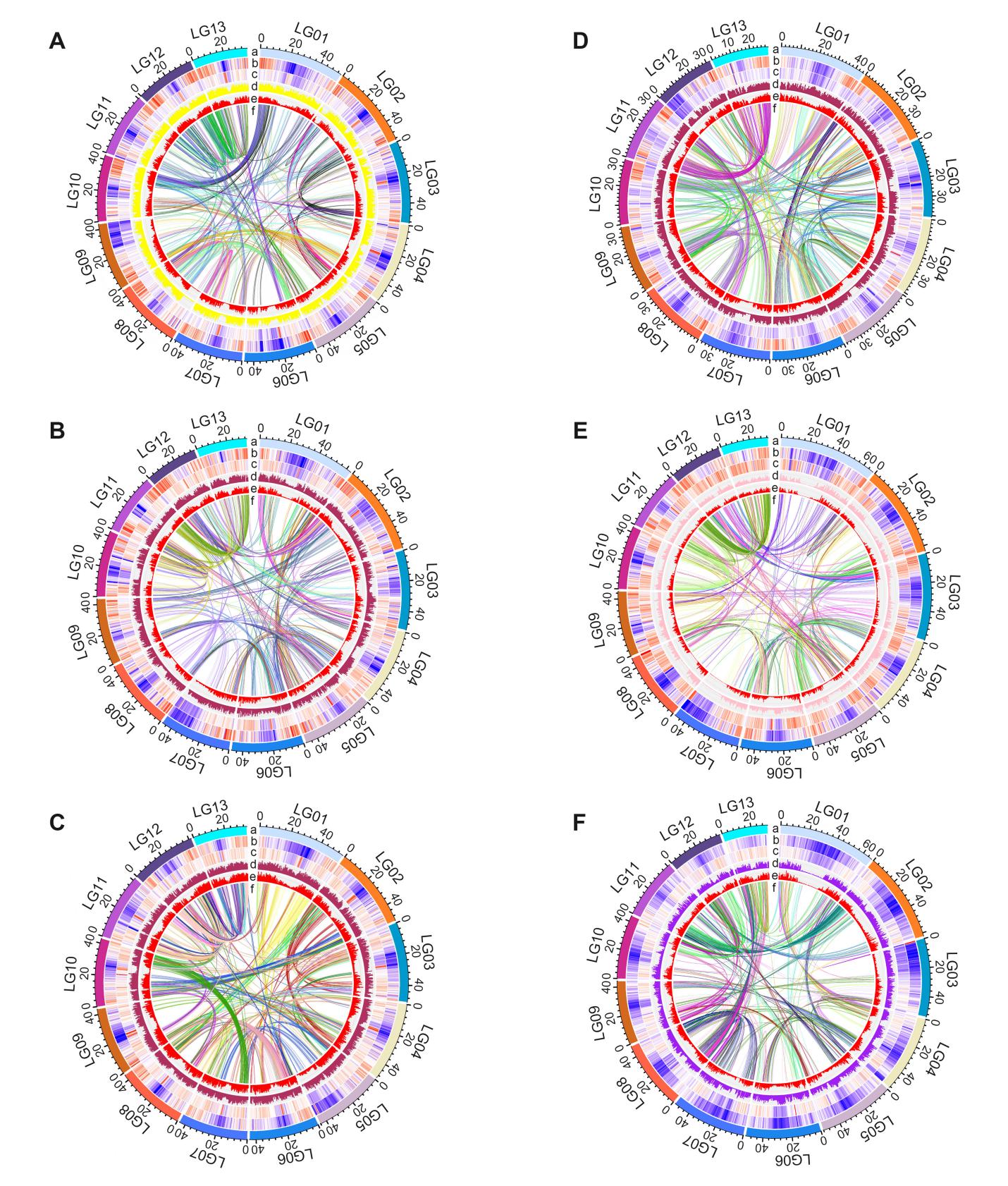


**Figure S1. Overview of the six rhododendron genome assemblies.**

**(A**–**F)** represent *R. nivale*, *R. principis*, *R. laudandum*, *R. latoucheae*, *R. fortunei* and *R. molle* respectively (in sequence). (a) The 13 pseudochromosomes, (b) gene density, (c) the density of repeat sequences, (d) the density of SNP, (e) the density of Indel, (f) collinear genomic blocks.


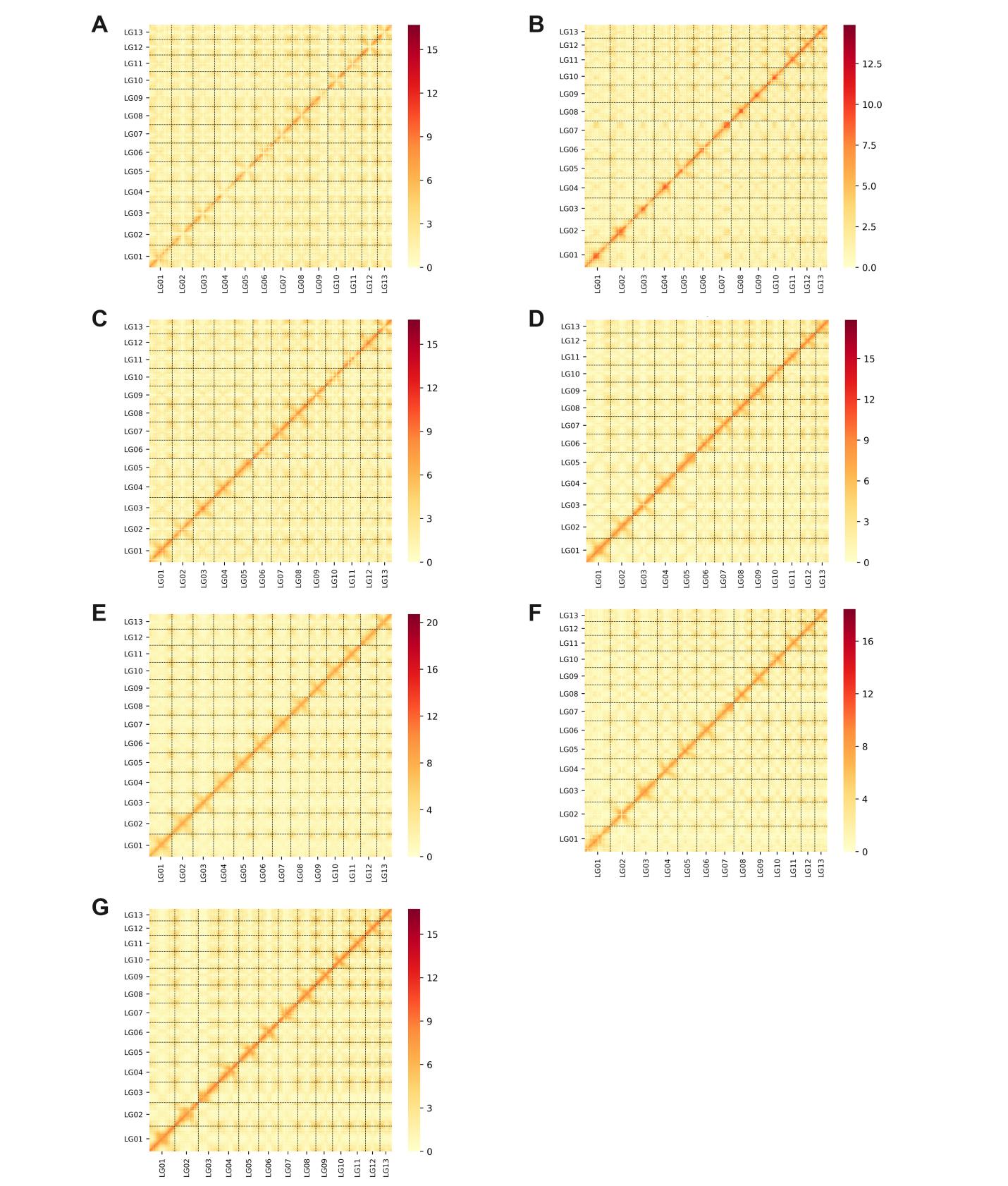


**Figure S2. Genome-wide all-by-all interaction maps of rhododendrons at 100-kb resolution.**

**(A**–**G)** represent *R. nivale*, *R. principis*, *R. laudandum*, *R. oreotrephes*, *R. latoucheae*, *R. fortunei* and *R. molle* respectively (in sequence).


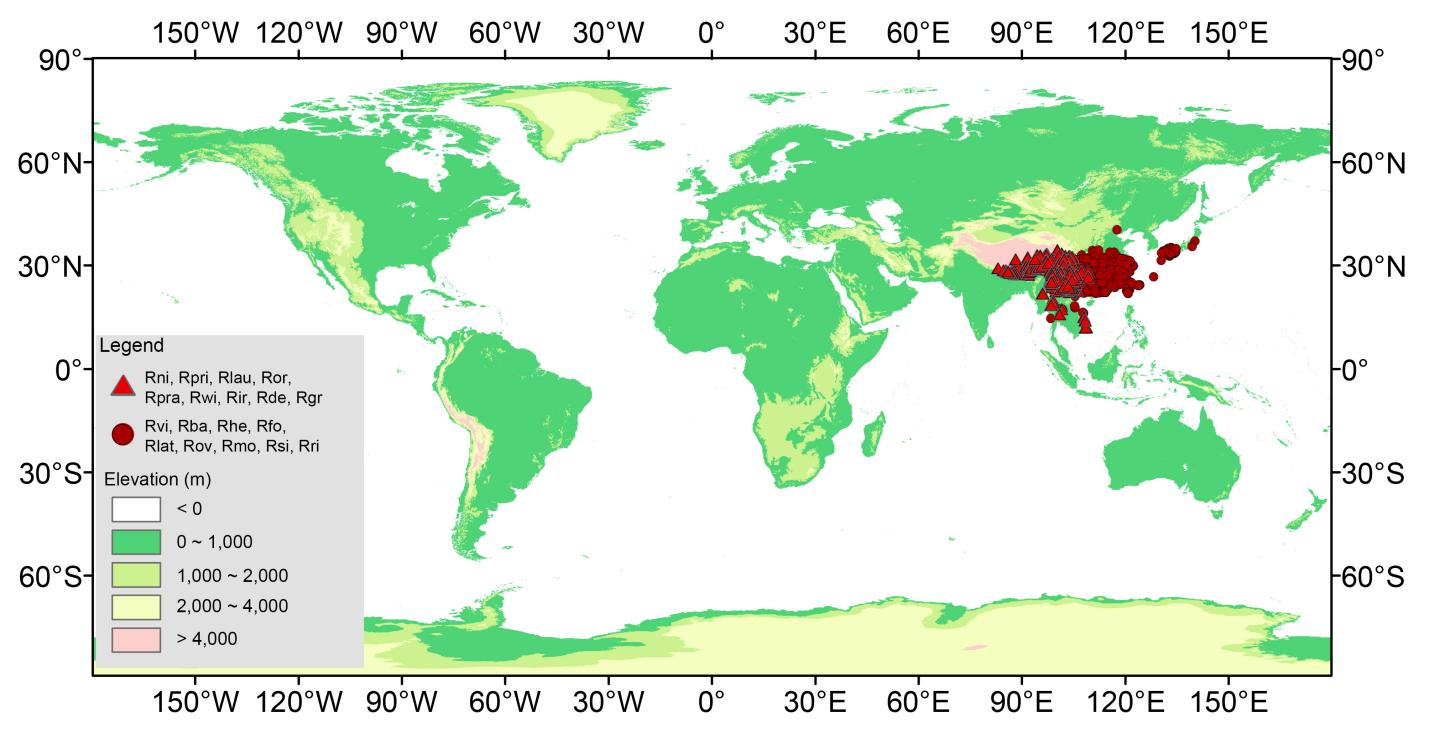


**Figure S3. Global distribution of the native regions of 18 *Rhododendron* species.**

The altitudes of different regions are indicated with different colors. The triangle represents the distribution positions of nine high-altitude *Rhododendron* species, while the circle represents the distribution positions of the other nine *Rhododendron* species. Rni–*Rhododendron nivale*, Rpri–*R. principis*, Rlau–*R. laudandum*, Ror–*R. oreotrephes*, Rpra–*R. prattii*, Rwi–*R. williamsianum*, Rir–*R. irroratum*, Rde–*R. delavayi*, Rgr–*R. griersonianum*, Rvi–*R. vialii*, Rba–*R. bailiense*, Rhe–*R. henanense* subsp*. lingbaoense*, Rfo–*R. fortunei*, Rlat–*R. latoucheae*, Rov–*R. ovatum*, Rmo–*R. molle*, Rsi–*R. simsii*, Rri–*R. ripense.*


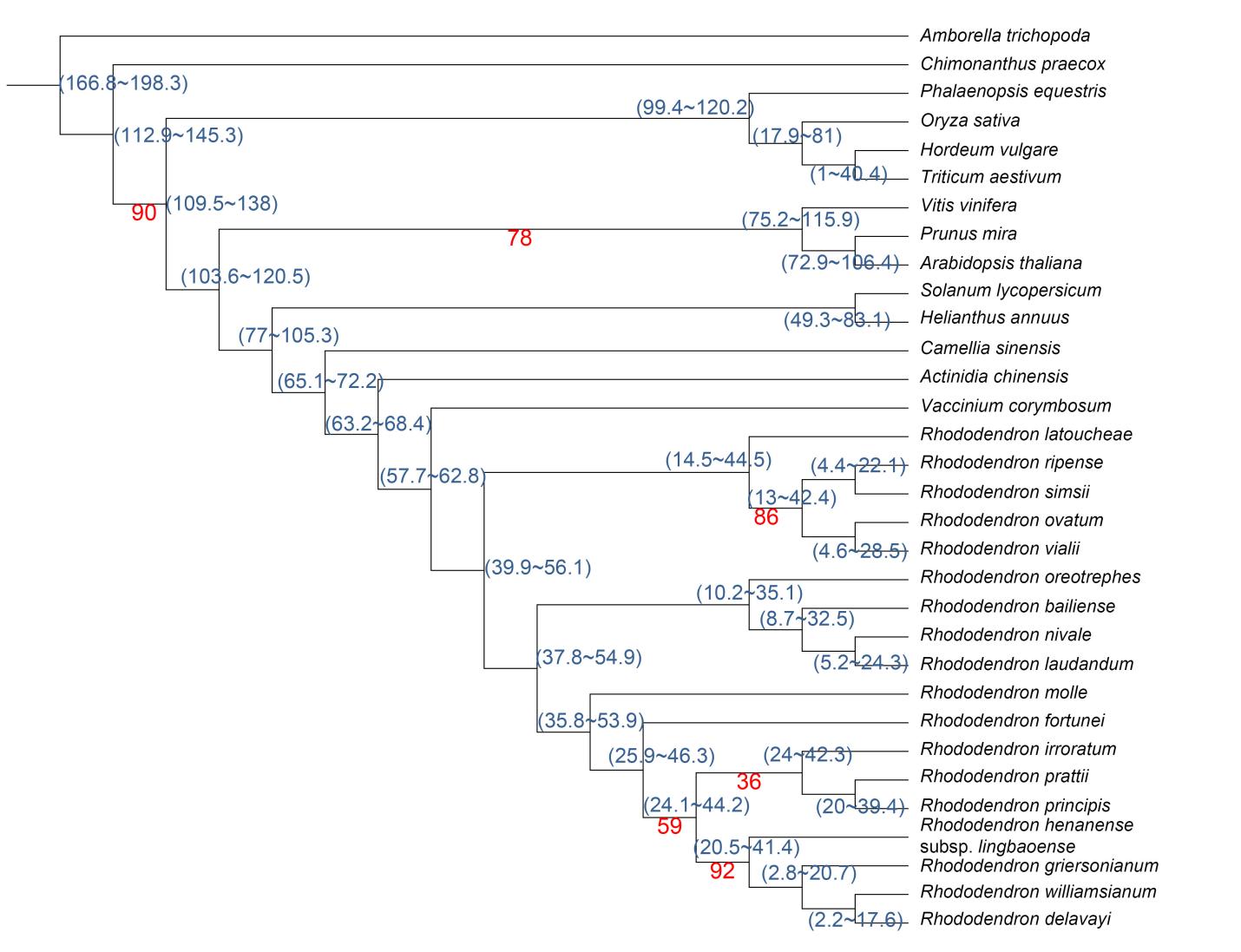


**Figure S4. Phylogenetic tree based on 490 orthologues from 32 species.**

The phylogenetic tree was constructed from 490 low-copy orthologues that were shared among 32 plants using the ML method. Non-100% bootstrap values are labeled in red font, while divergence times (with 95% confidence interval) are indicated in blue text.


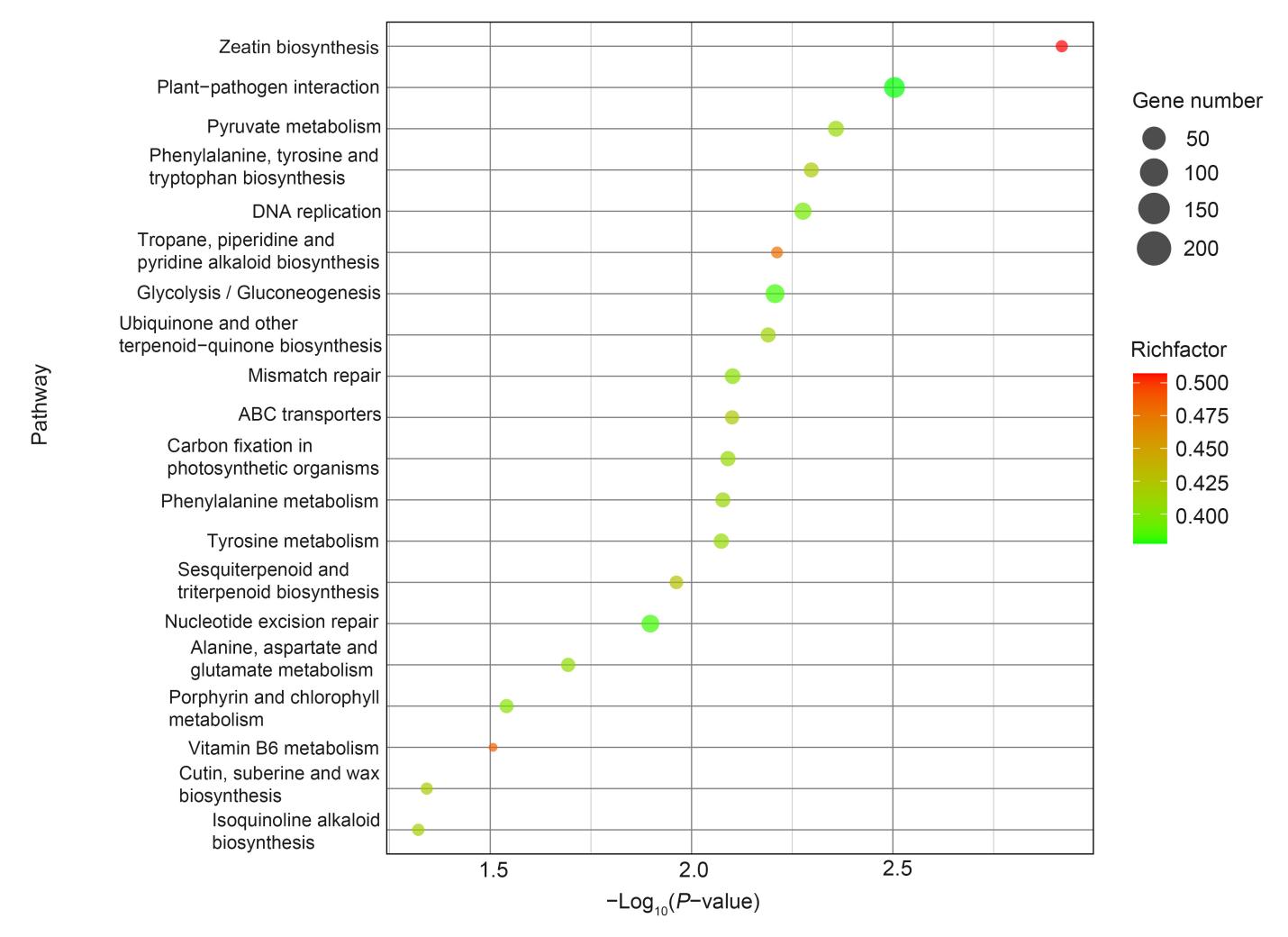


**Figure S5. KEGG enrichment analysis of contracted gene families in subgenus *Tsutsusi*.**


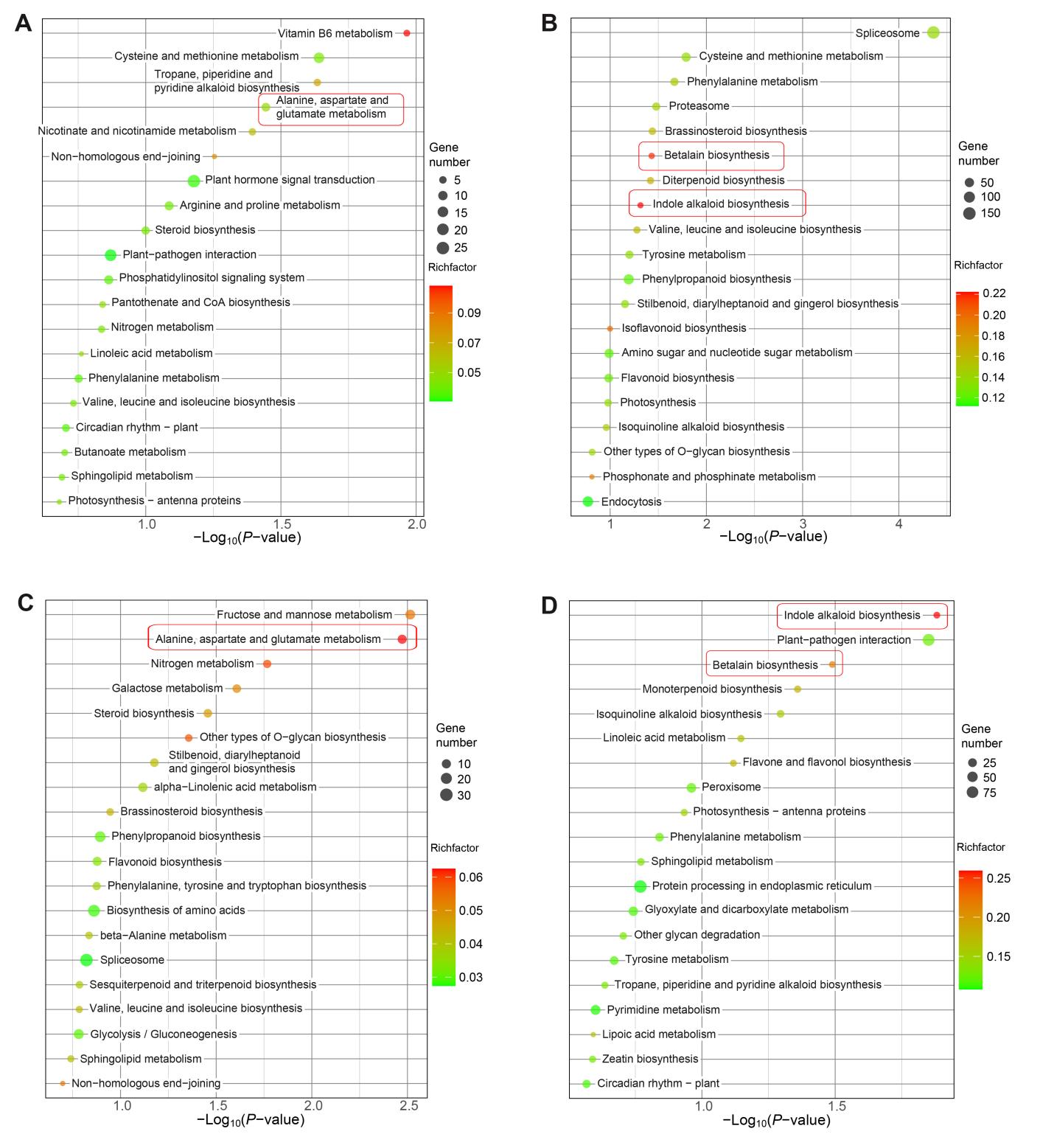


**Figure S6. KEGG enrichment analysis of expanded and contracted gene families in *R. nivale* and *R. laudandum*.**

**(A)** Expanded gene families in *R. nivale*. **(B)** Contracted gene families in *R. nivale*. **(C)** Expanded gene families in *R. laudandum*. **(D)** Contracted gene families in *R. laudandum*.


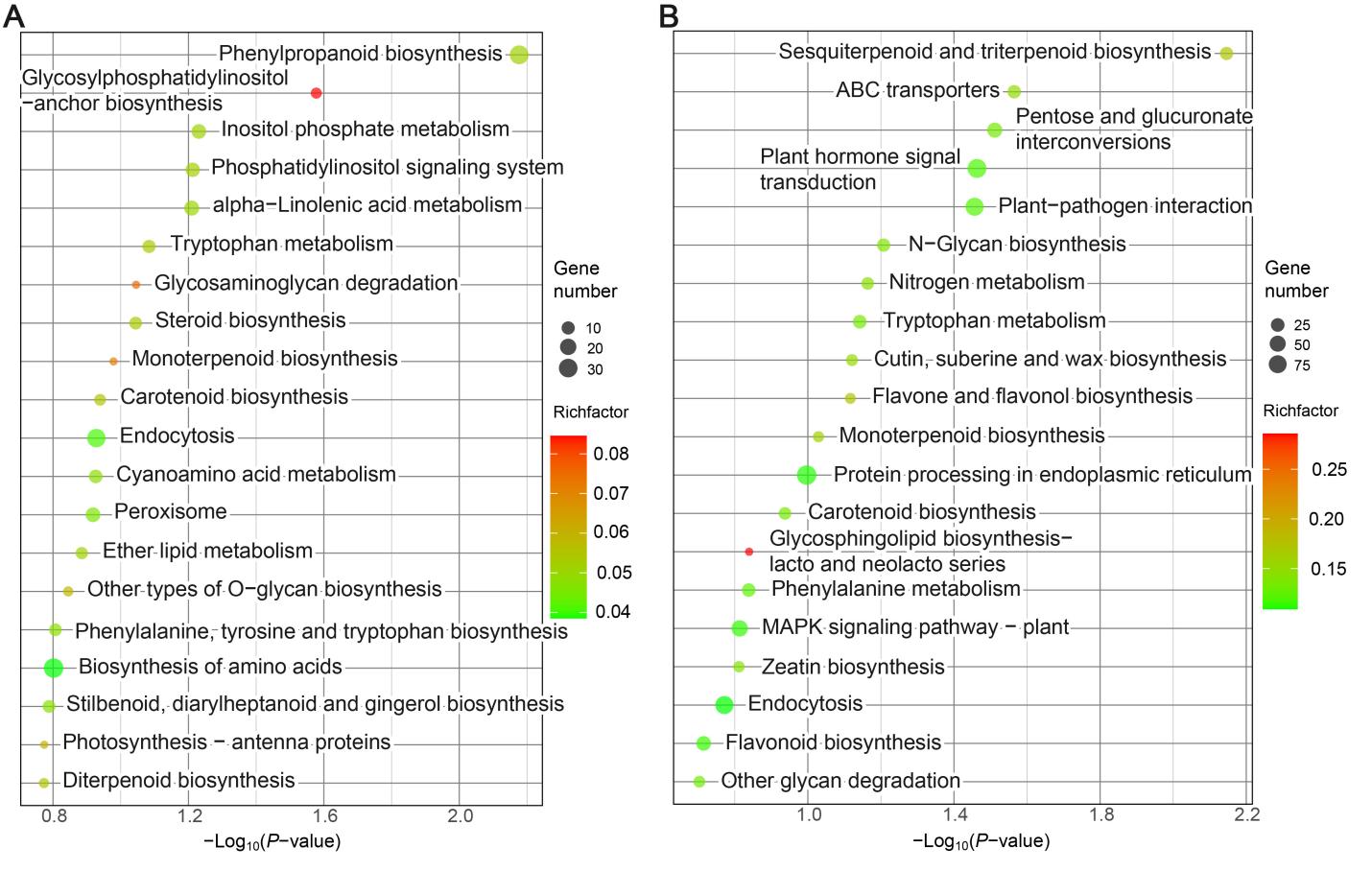


**Figure S7. KEGG pathway enrichment distribution of expanded and contraction gene families in *R. principis*.**

**(A)** Expanded gene families. **(B)** Contraction gene families.


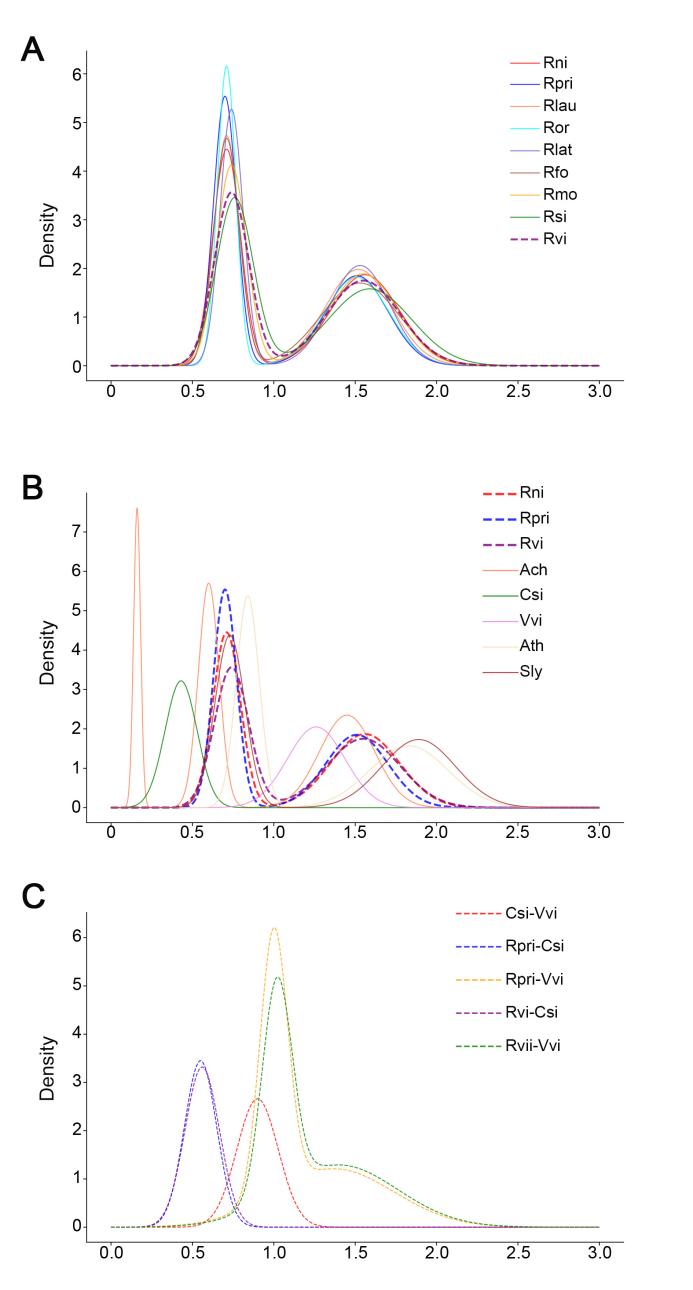


**Figure S8. Whole-genome duplication (WGD) events of *Rhododendron* species.**

The distributions of synonymous substitution levels (*Ks*) for paralogous genes in nine rhododendrons and five outgroup plants **(A**, **B)**. Ach–*Actinidia chinensis*, Csi–*Camellia sinensis*, Vvi–*Vitis vinifera*, Ath–*Arabidopsis thaliana*, Sly–*Solanum lycopersicum*. **(C)** *Ks* distributions from syntenic orthologous between four species.


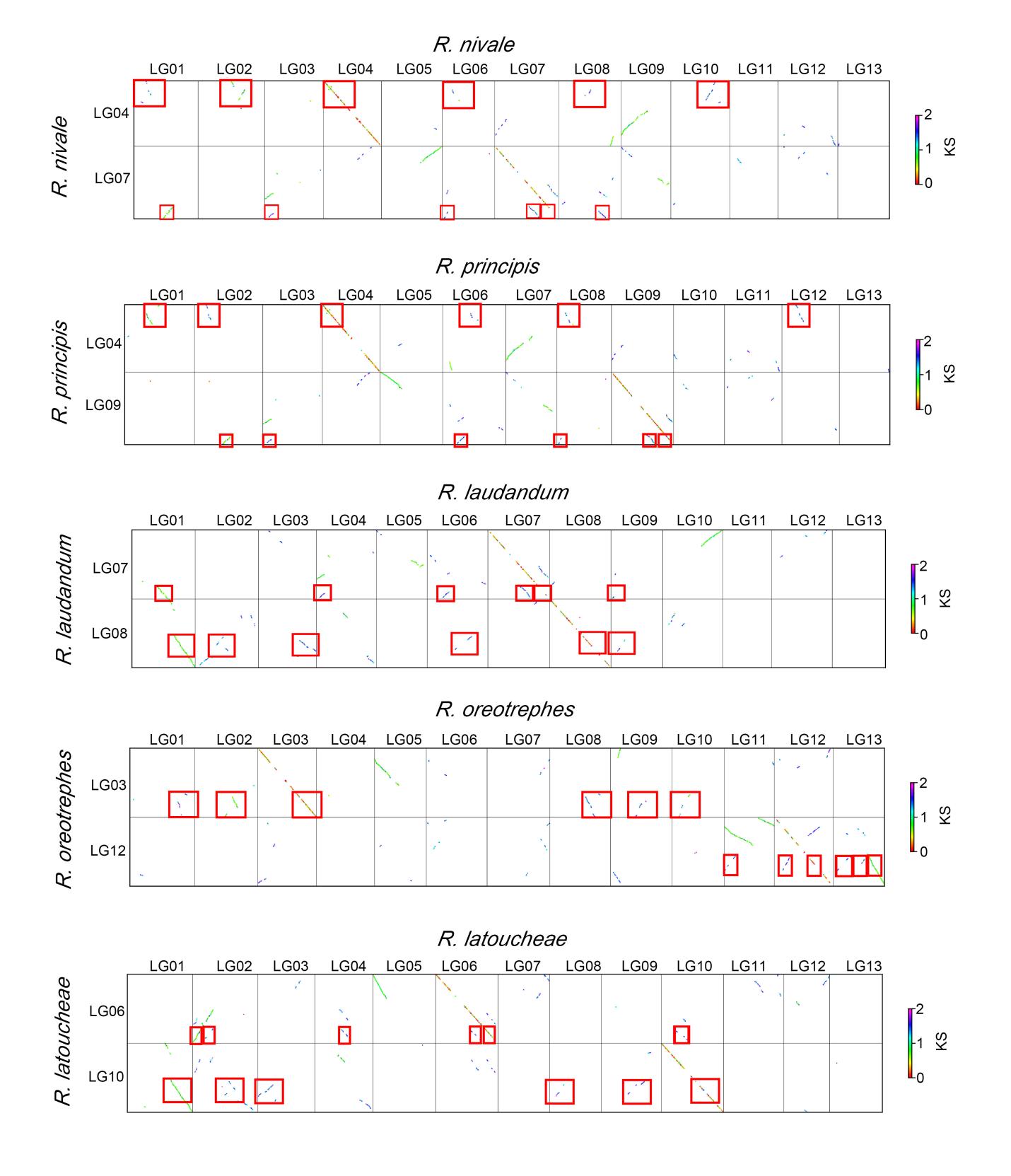


**Figure S9. Syntenic blocks of *R. nivale*, *R. principis*, *R. laudandum, R. oreotrephes* and *R. latoucheae* genomes.**


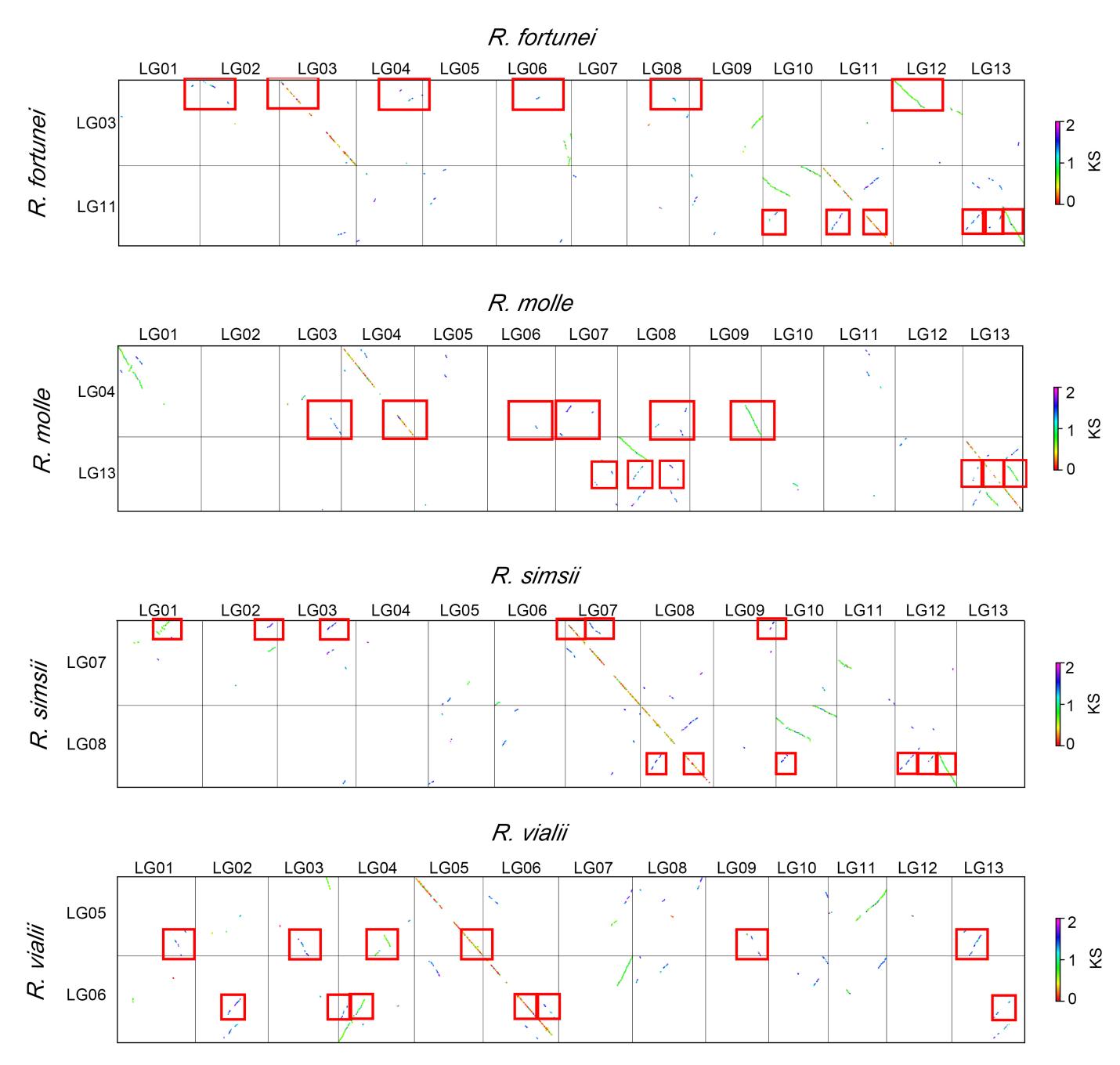


**Figure S10. Syntenic blocks of *R. fortunei*, *R. molle*, *R. simsii* and *R. vialii* genomes.**


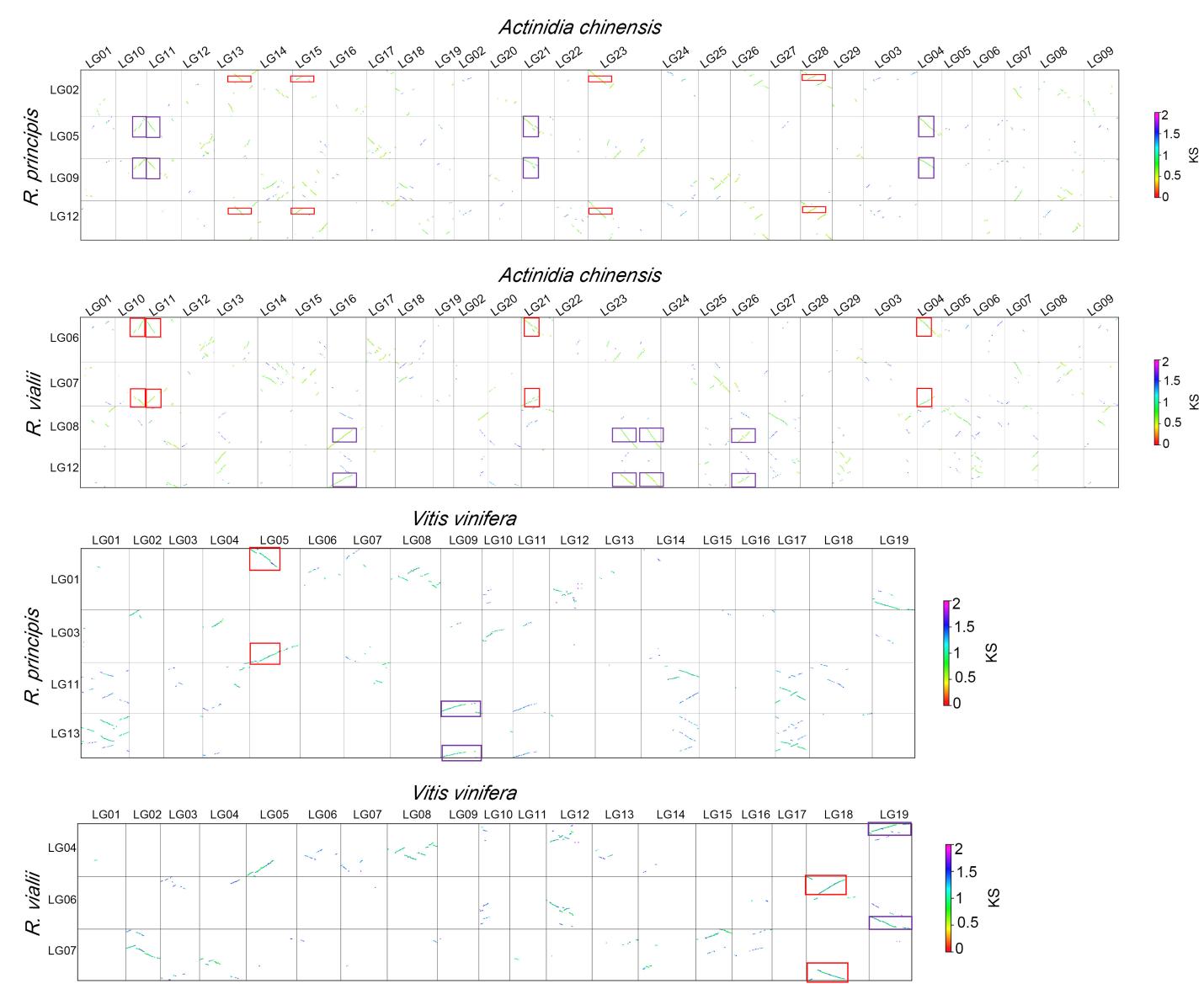


**Figure S11. Homologous blocks between rhododendrons and kiwifruit and grape genomes.**

Dot plots of orthologs show a 4:2 chromosomal relationship between the *A. chinensis* genome and *R. principis*/ *R. vialii* genome; and a 1:2 chromosomal relationship between the *V. vinifera* genome and *R. principis*/ *R. vialii* genome.


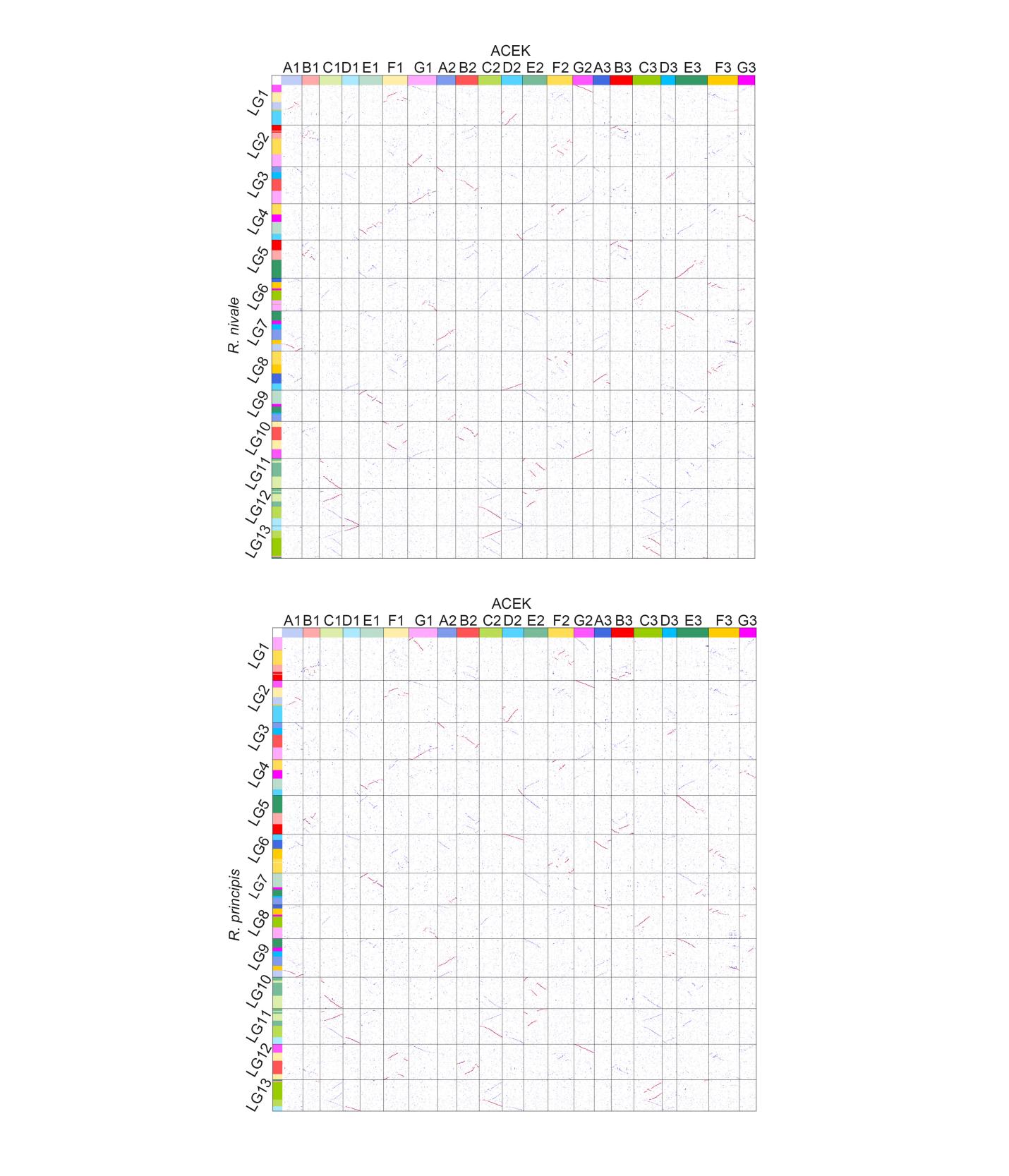


**Figure S12. Genomic comparison between ACEK and *R. nivale*/ *R. principis* based on dotplot.**

The red dots represent imprints of ancestral chromosomes on existing chromosomes.


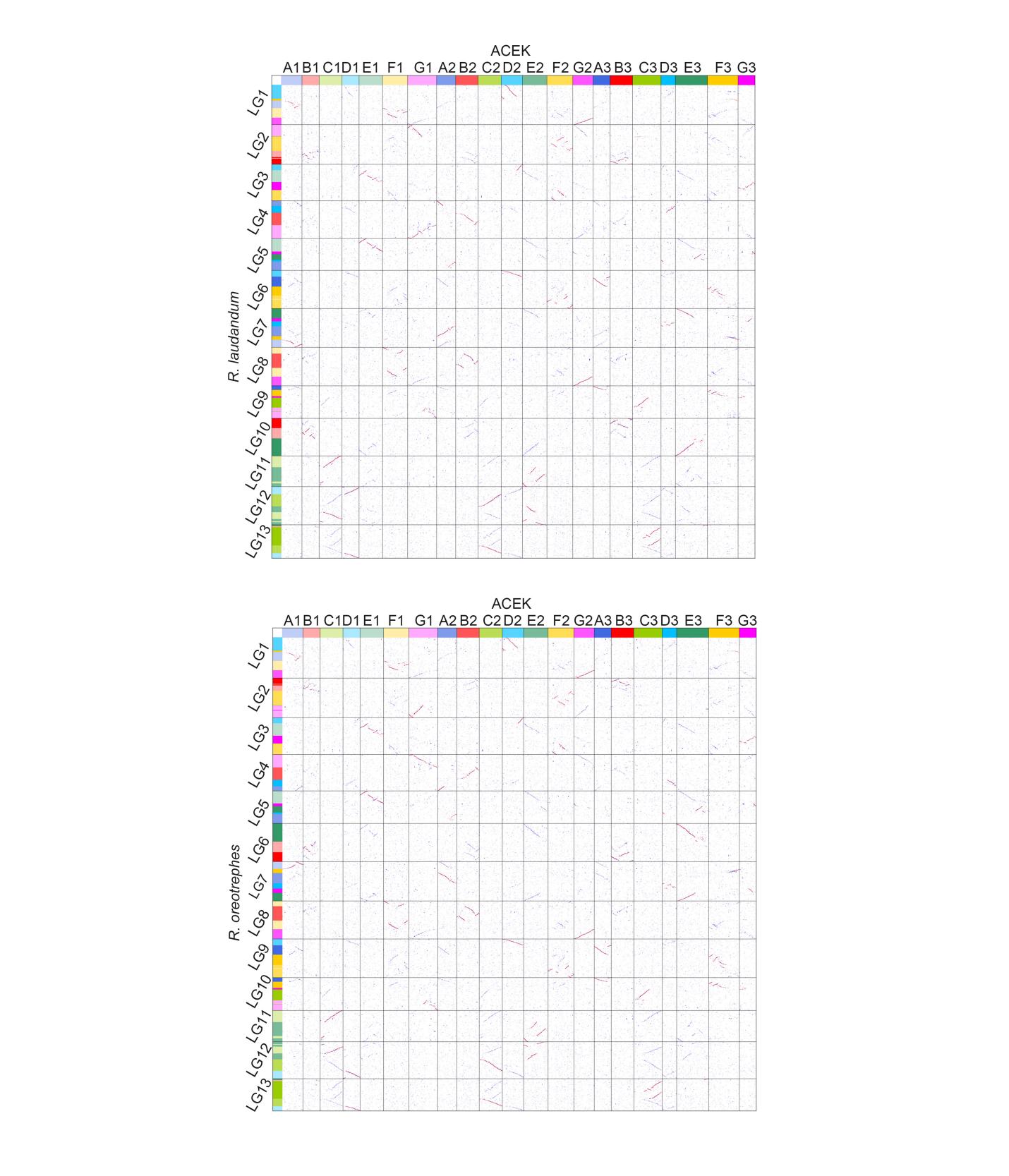


**Figure S13. Genomic comparison between ACEK and *R. laudandum*/ *R. oreotrephes* based on dotplot.**


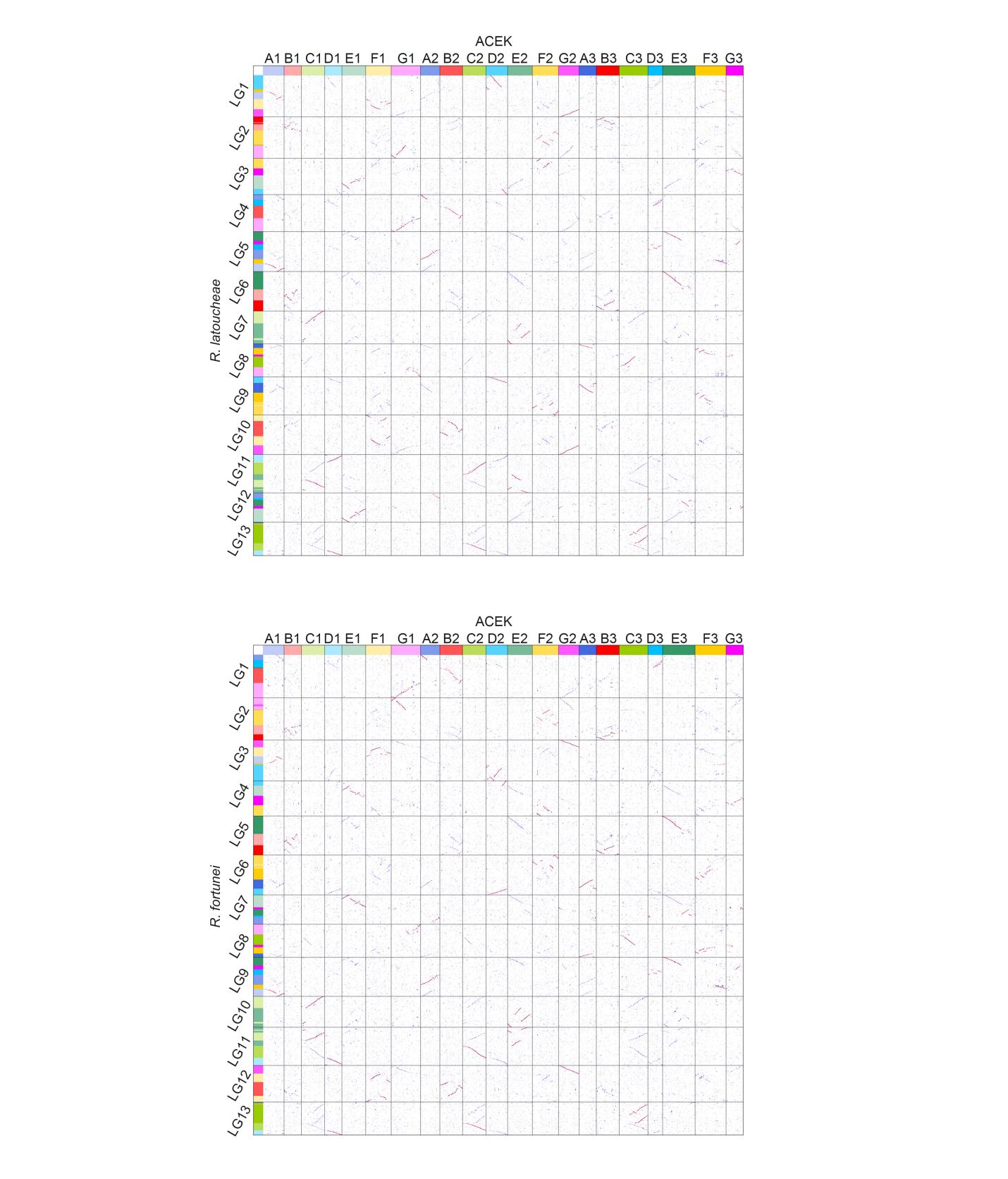


**Figure S14. Genomic comparison between ACEK and *R. latoucheae*/ *R. fortunei* based on dotplot.**


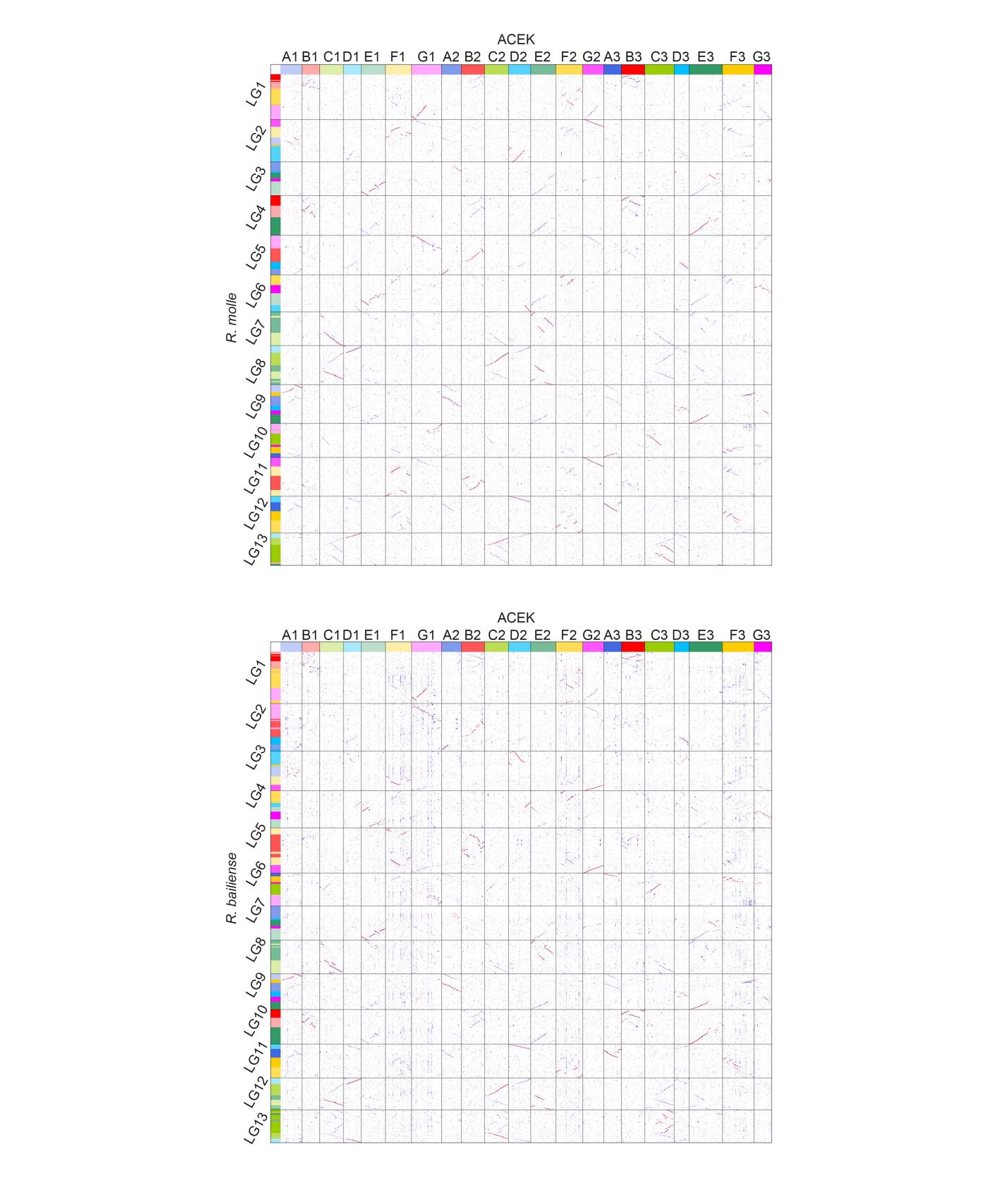


**Figure S15. Genomic comparison between ACEK and *R. molle*/ *R. bailiense* based on dotplot.**


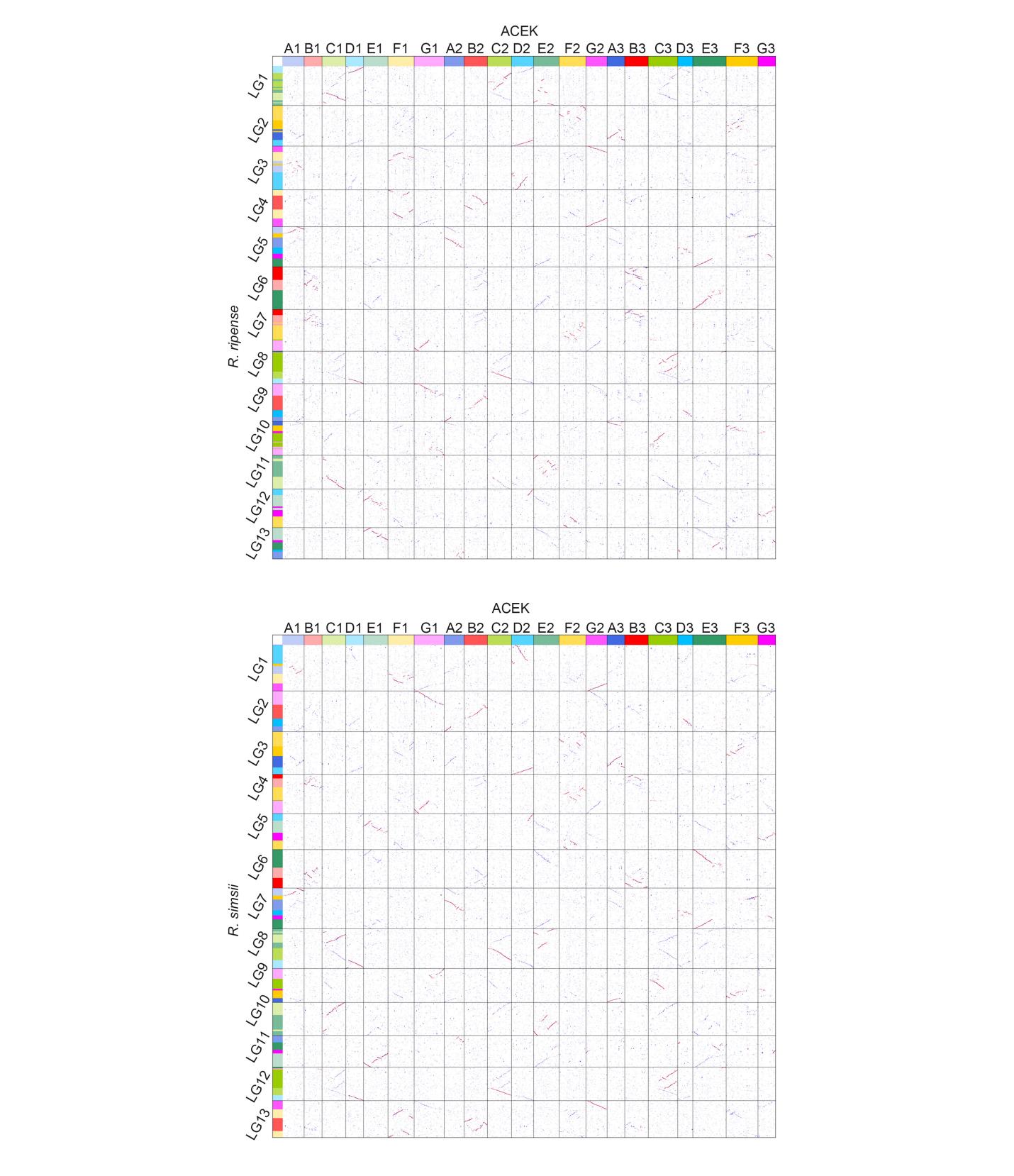


**Figure S16. Genomic comparison between ACEK and *R. ripense*/ *R. simsii* based on dotplot.**


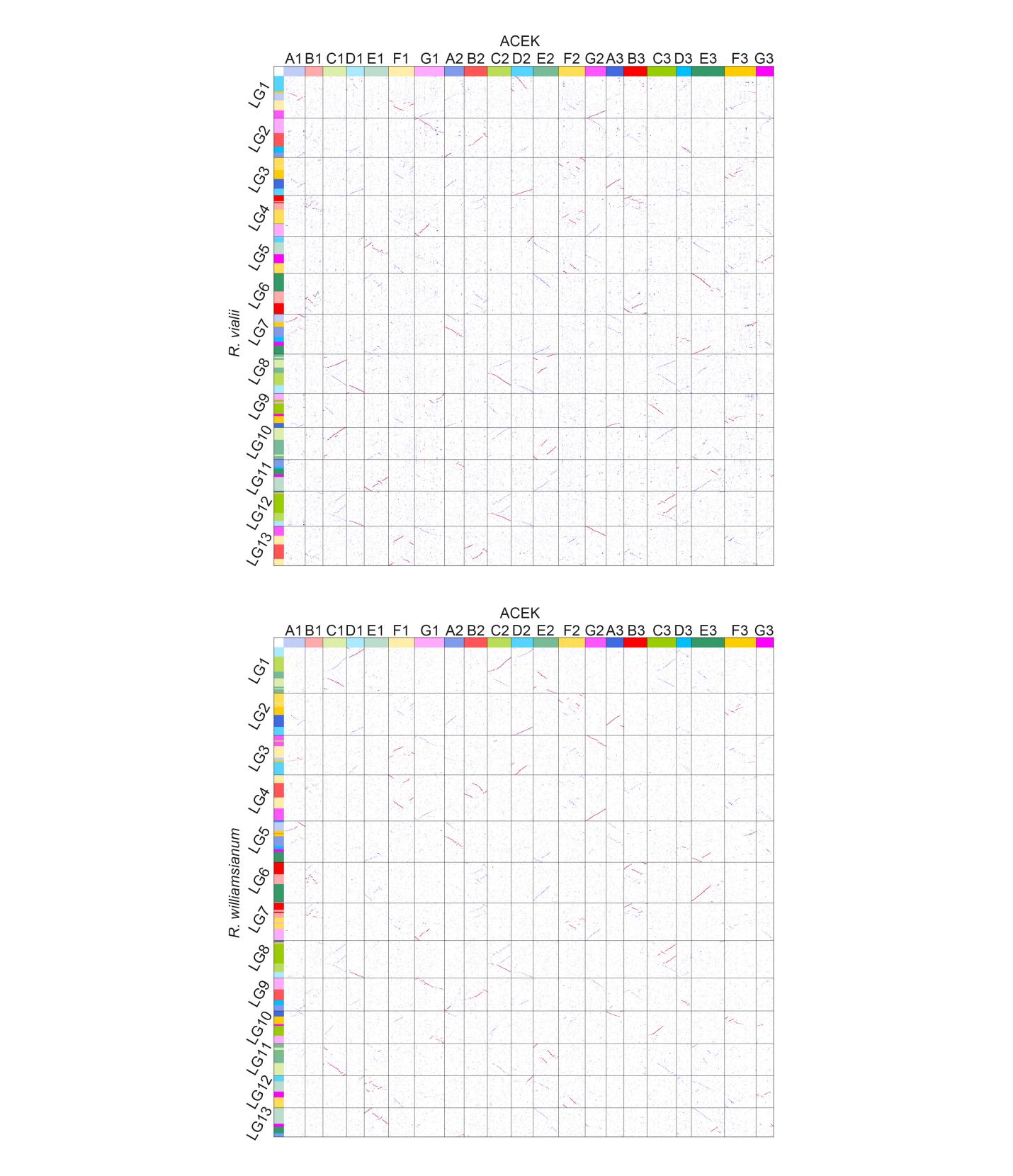


**Figure S17. Genomic comparison between ACEK and *R. vialii*/ *R. williamsianum* based on dotplot.**


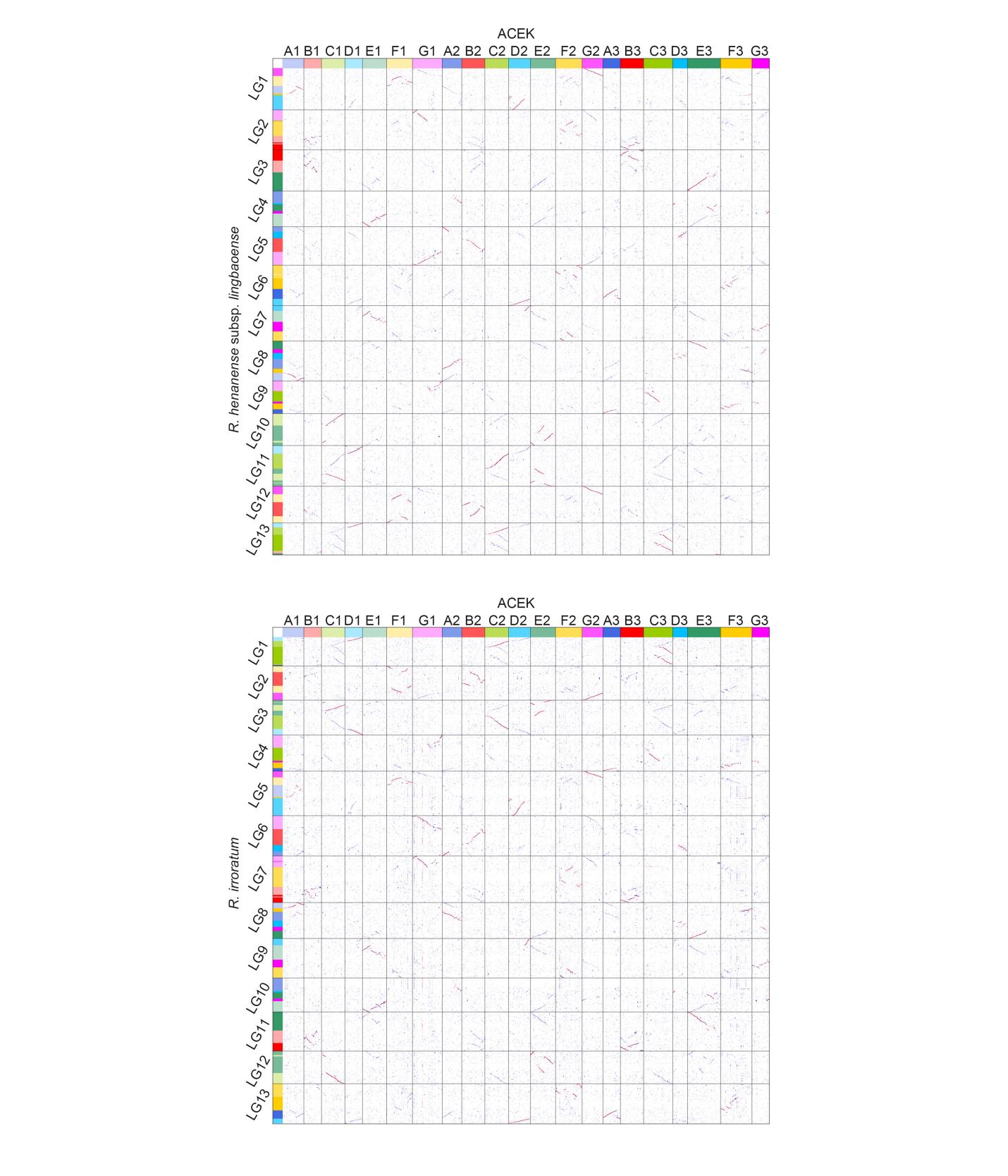


**Figure S18. Genomic comparison between ACEK and *R. henanense* subsp*. lingbaoense*/ *R. irroratum* based on dotplot.**


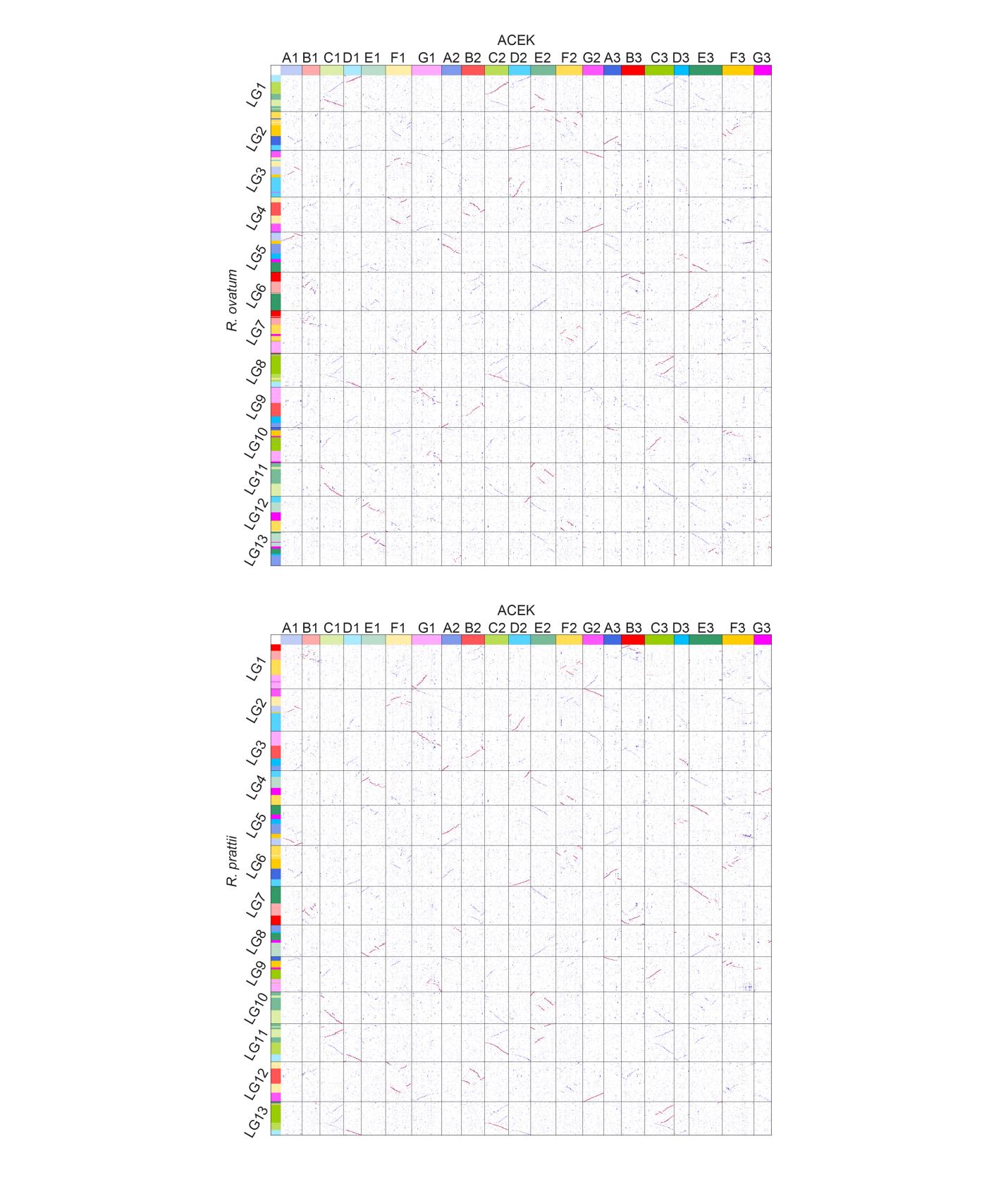


**Figure S19. Genomic comparison between ACEK and *R. ovatum*/ *R. prattii* based on dotplot.**

**
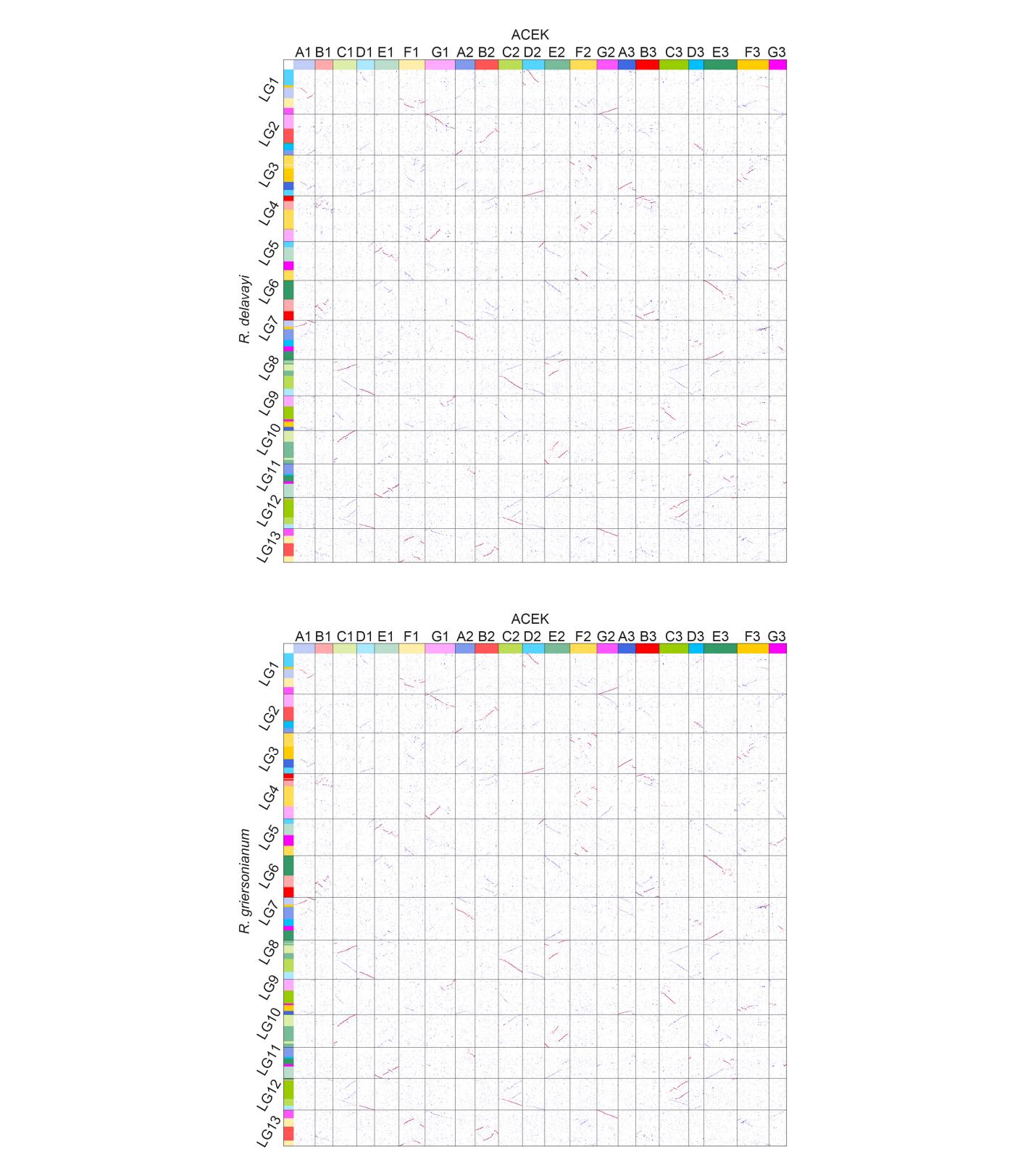
**

**Figure S20. Genomic comparison between ACEK and *R. delavayi*/ *R. griersonianum* based on dotplot.**


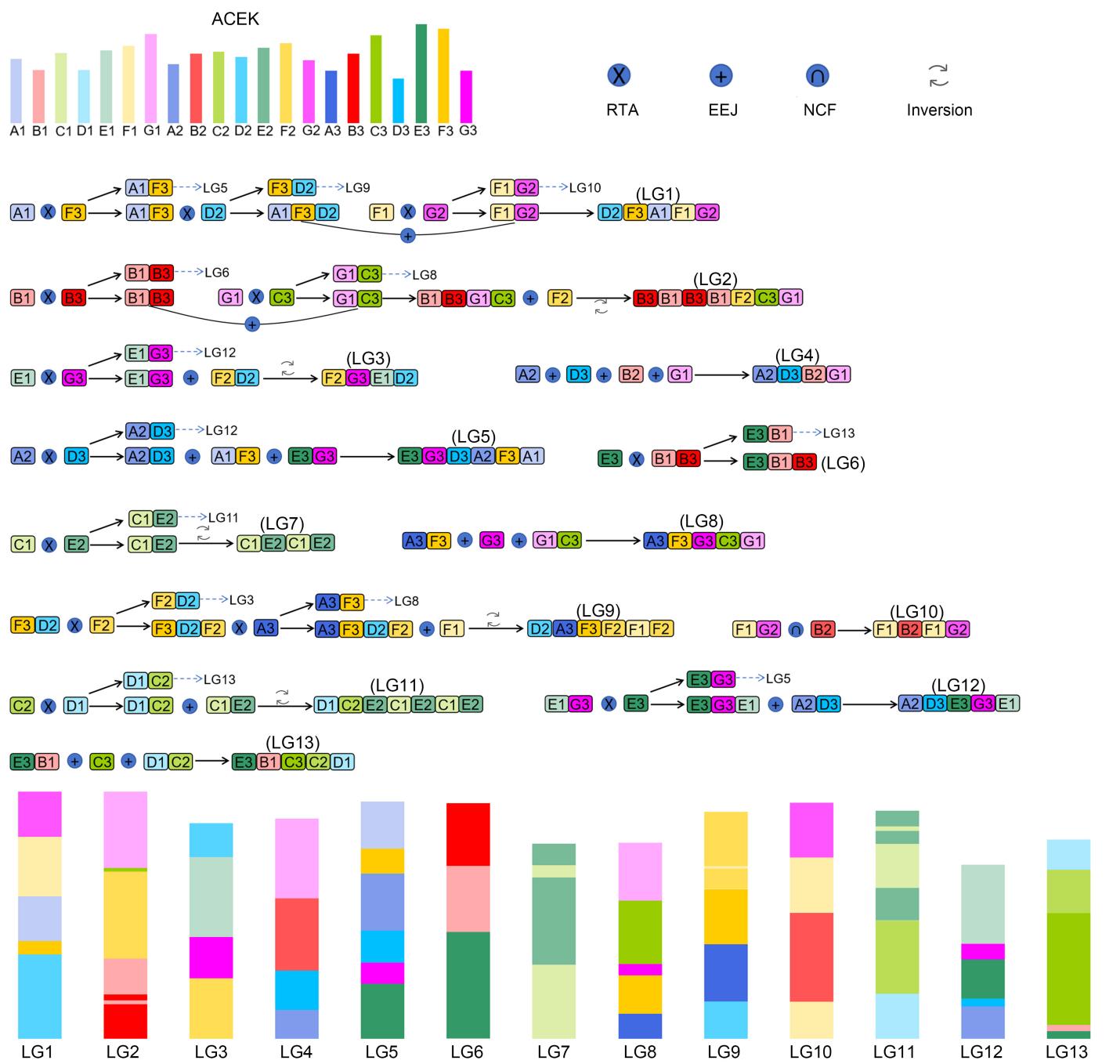


**Figure S21. Derivation of karyotype evolution of *R. latoucheae*.**

The current 13 chromosomes were formed by the rupture and fusion of 21 ancestral chromosomes.


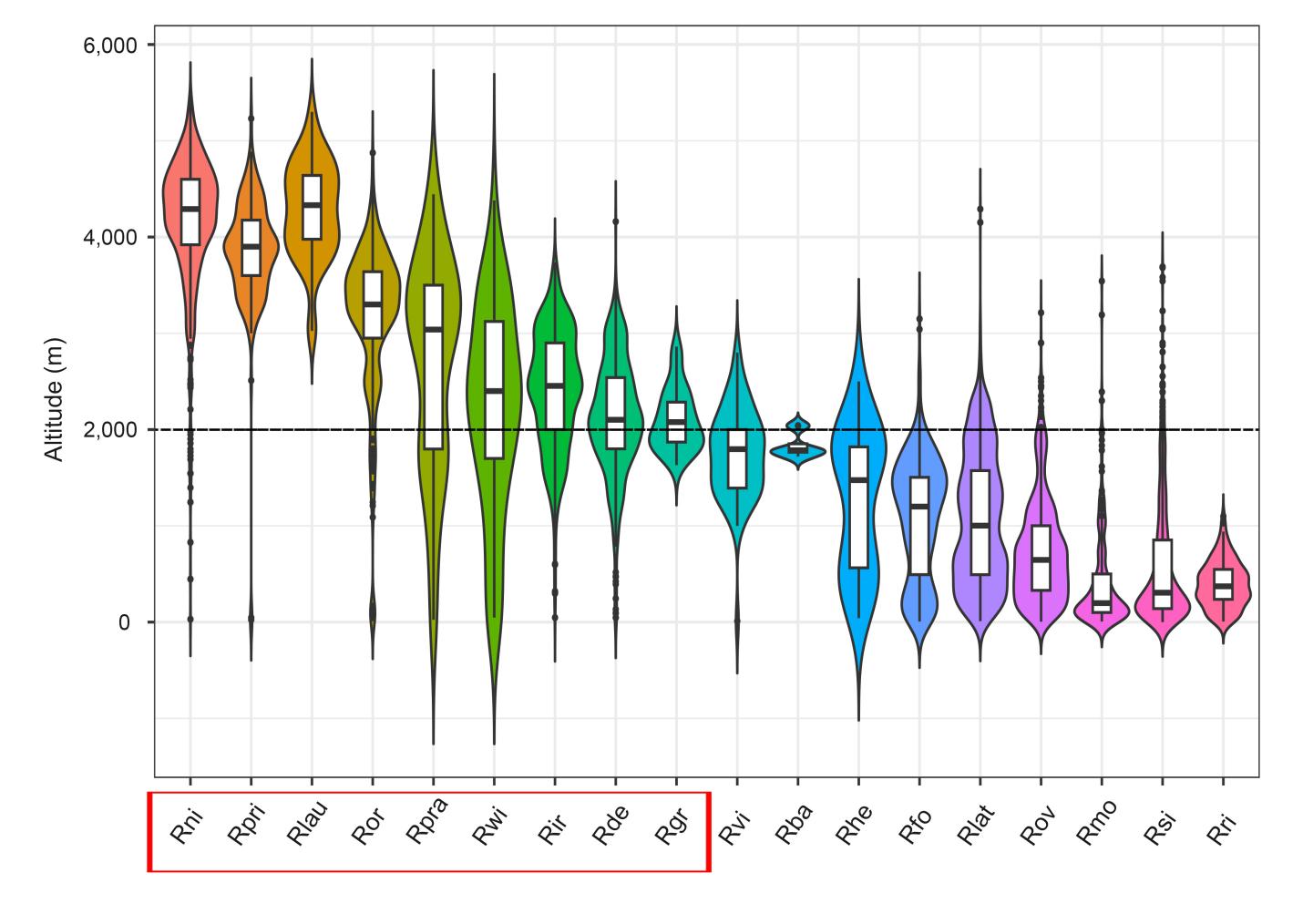


**Figure S22. Global altitude distribution statistics of 18 *Rhododendron* species, including records of cultivated introductions.**

Nine species of rhododendron are mainly distributed above an altitude of 2,000 meters.


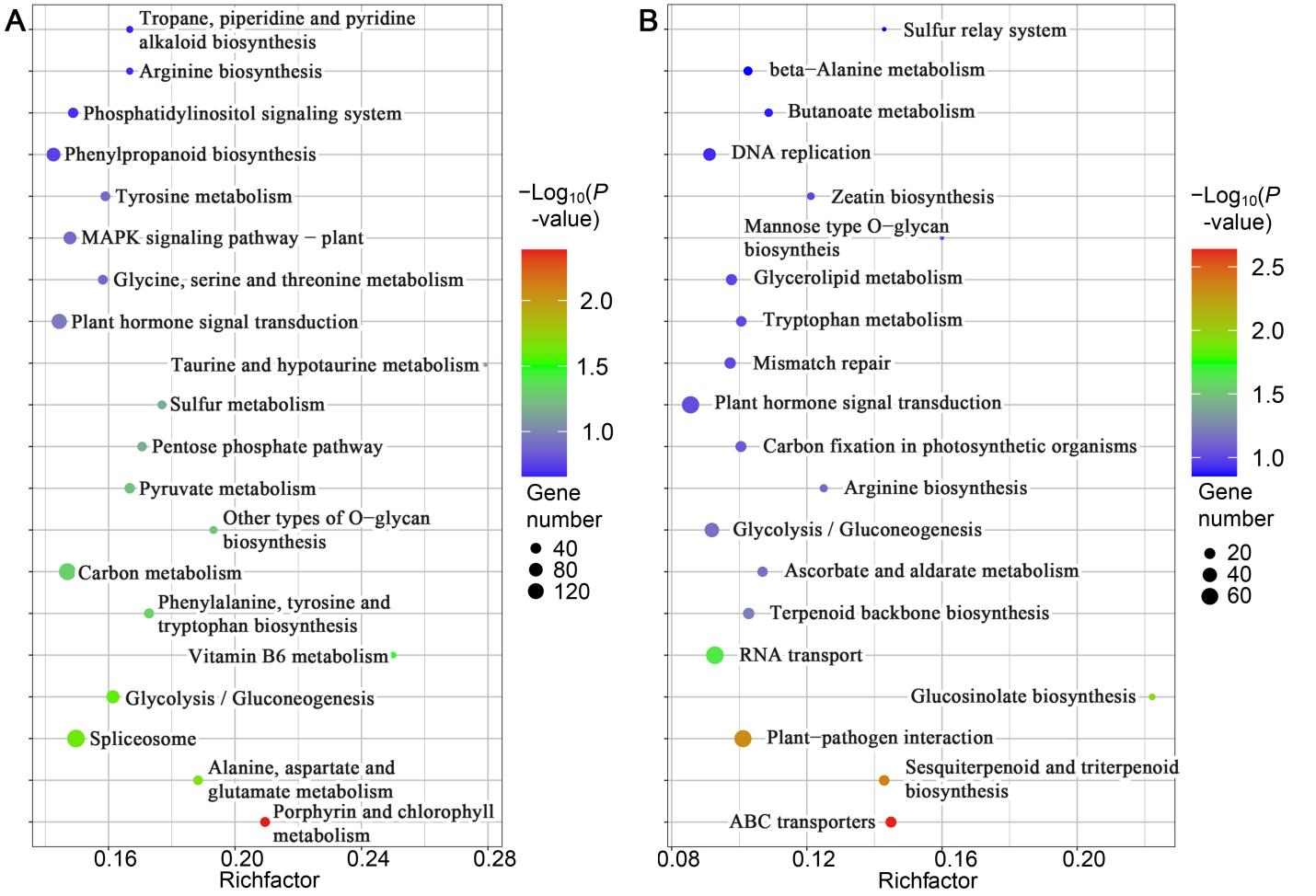


**Figure S23. KEGG pathway enrichment distribution of the unique-gene families of rhododendrons at different altitudes.**

**(A)** Unique-gene families in high-altitude rhododendrons. **(B)** Unique-gene families in low-altitude rhododendrons.


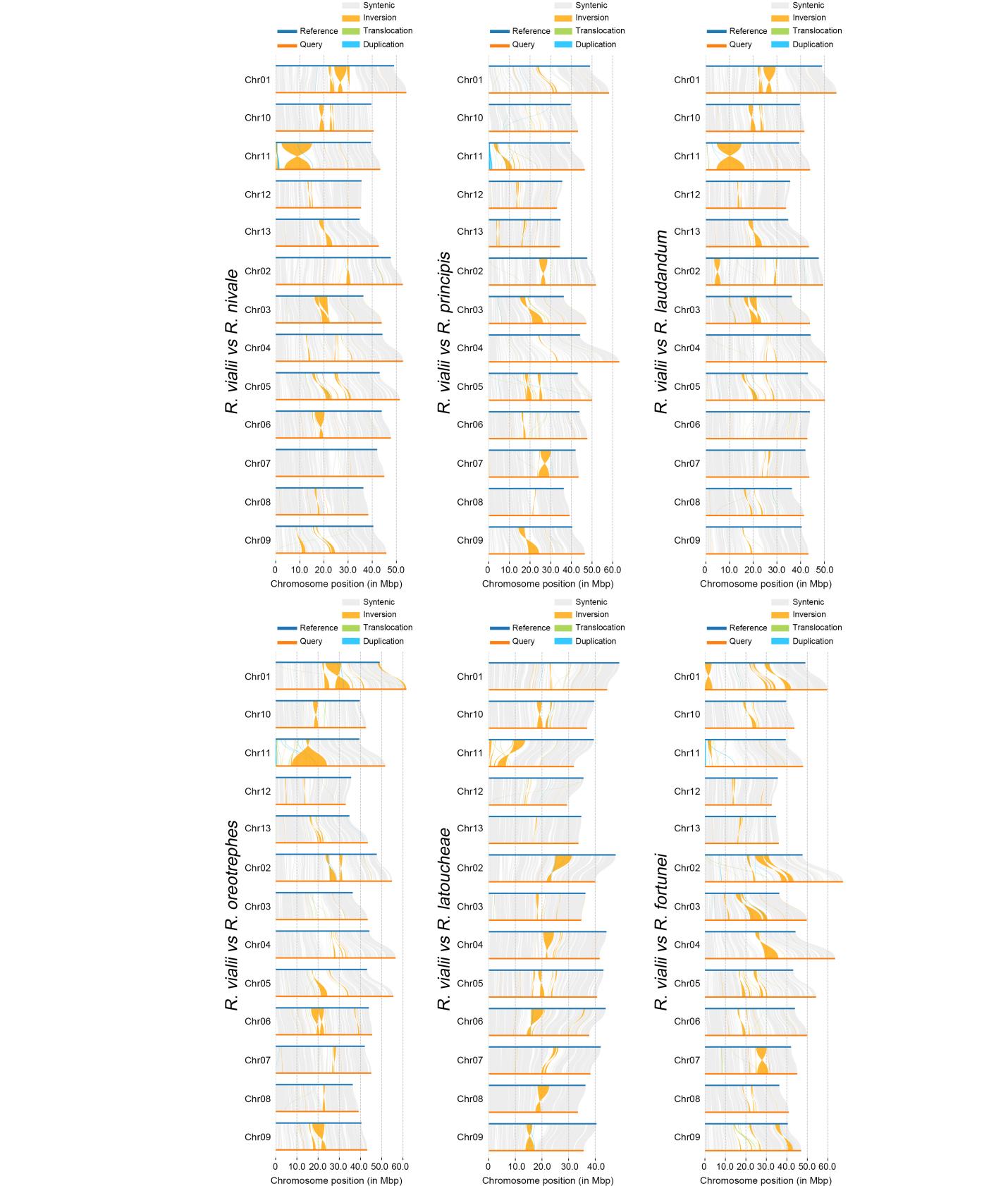


**Figure S24. Homology and rearrangement of *R. nivale*, *R. principis*, *R. laudandum*, *R. oreotrephes*, *R. latoucheae* and *R. fortunei* genome using *R. vialii* as reference.**


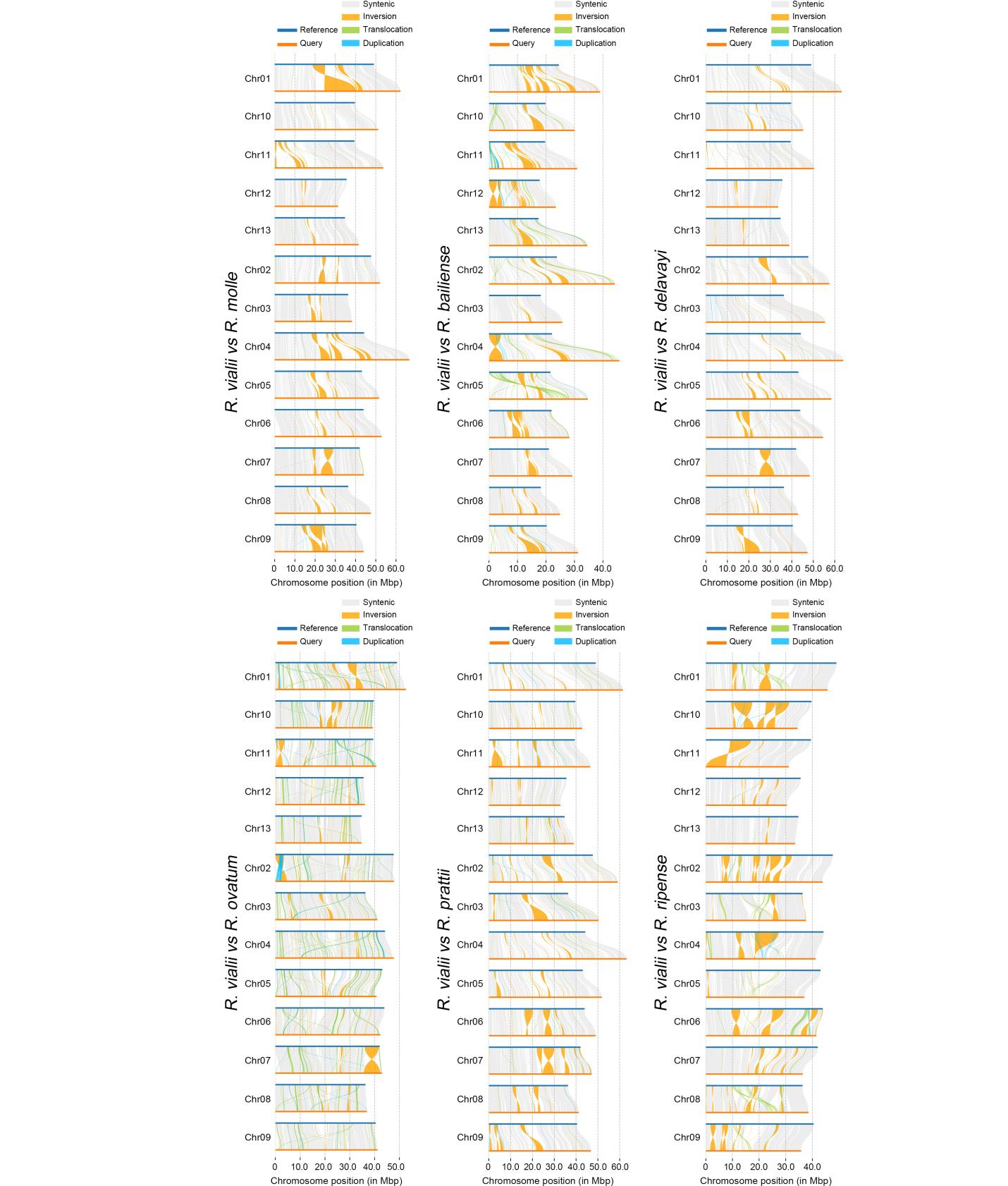


**Figure S25. Homology and rearrangement of *R. molle*, *R. bailiense*, *R. delavayi*, *R. ovatum*, *R. prattii* and *R. ripense* genome using *R. vialii* as reference.**


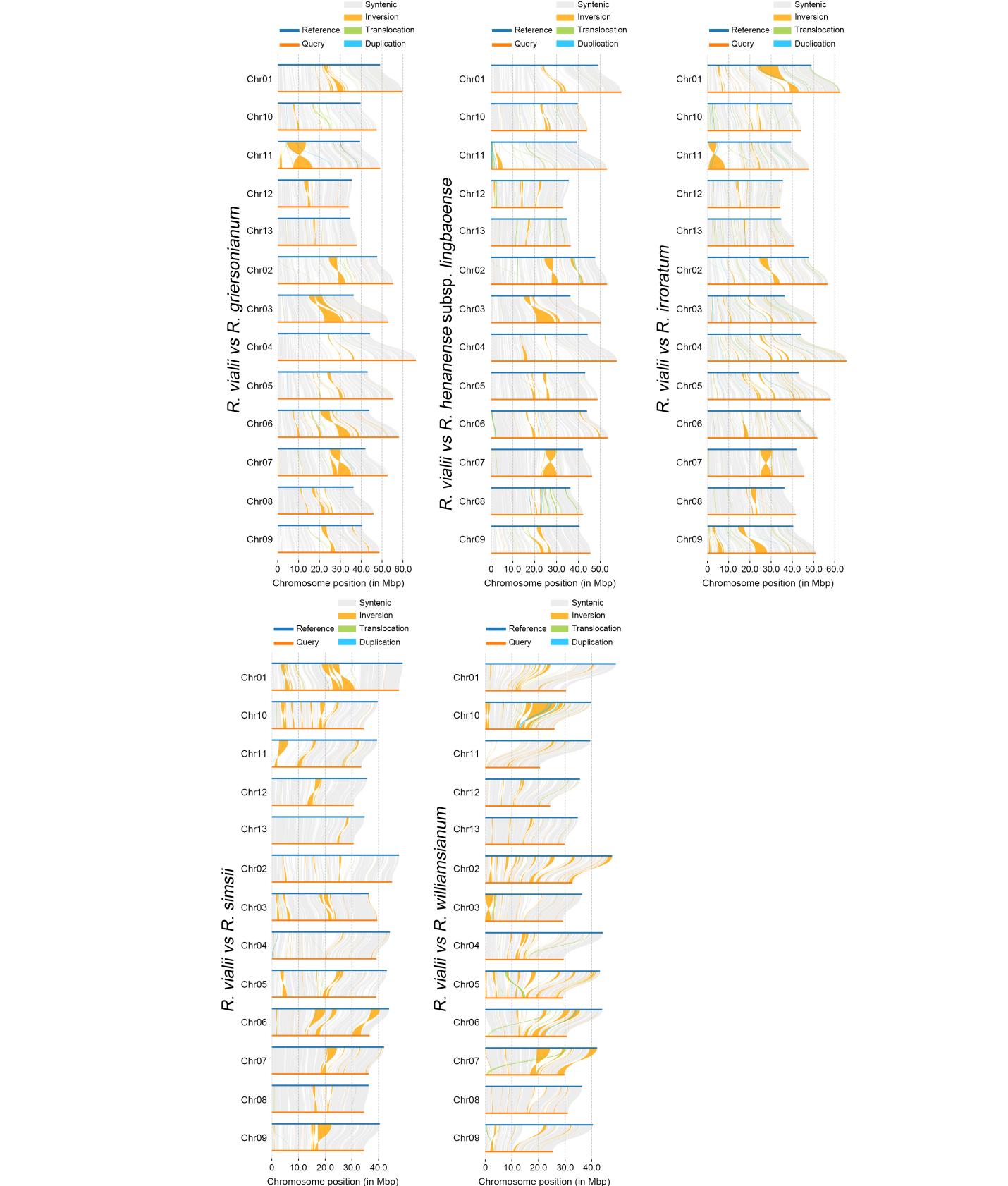


**Figure S26. Homology and rearrangement of *R. griersonianum*, *R. henanense* subsp. *lingbaoense*, *R. irroratum*, *R. simsii* and *R. williamsianum* genome using *R. vialii* as reference.**


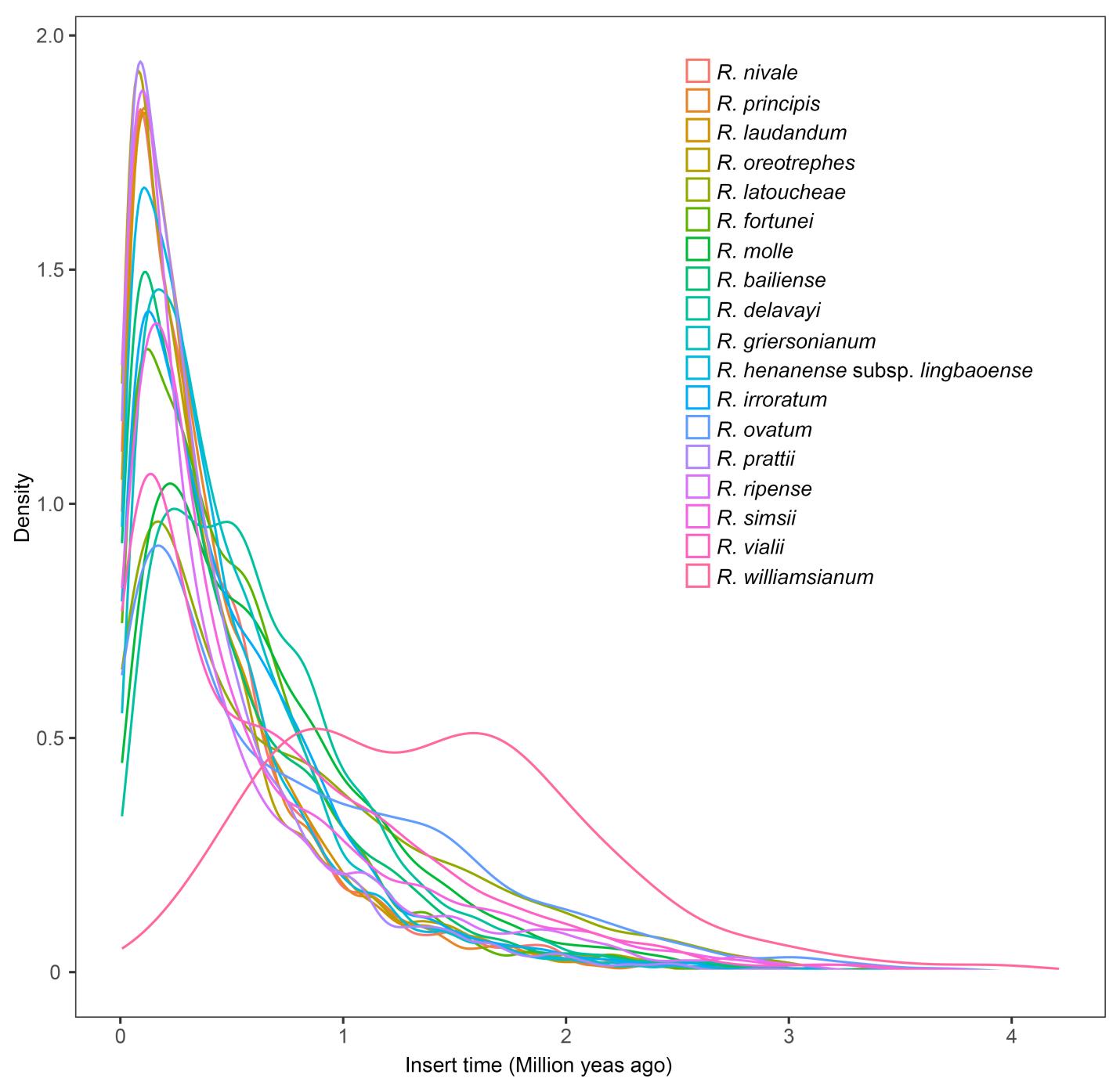


**Figure S27. Insertion time of LTRs in 18 *Rhododendron* species.**


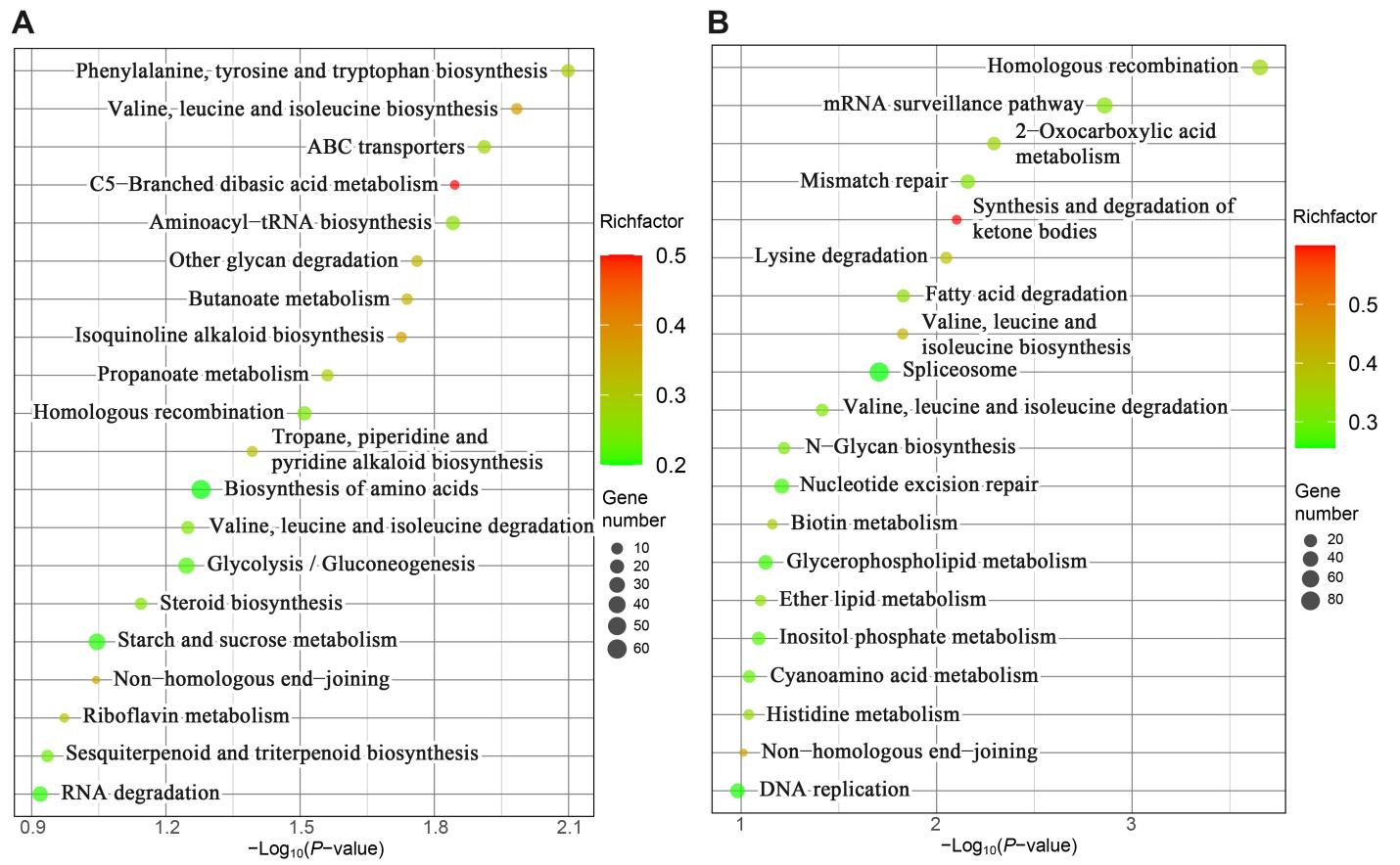


**Figure S28. KEGG pathway enrichment distribution of genes in the 1k downstream region of the unique LTRs of two rhododendrons.**

**(A)** *R. simsii*. **(B)** *R. molle*.


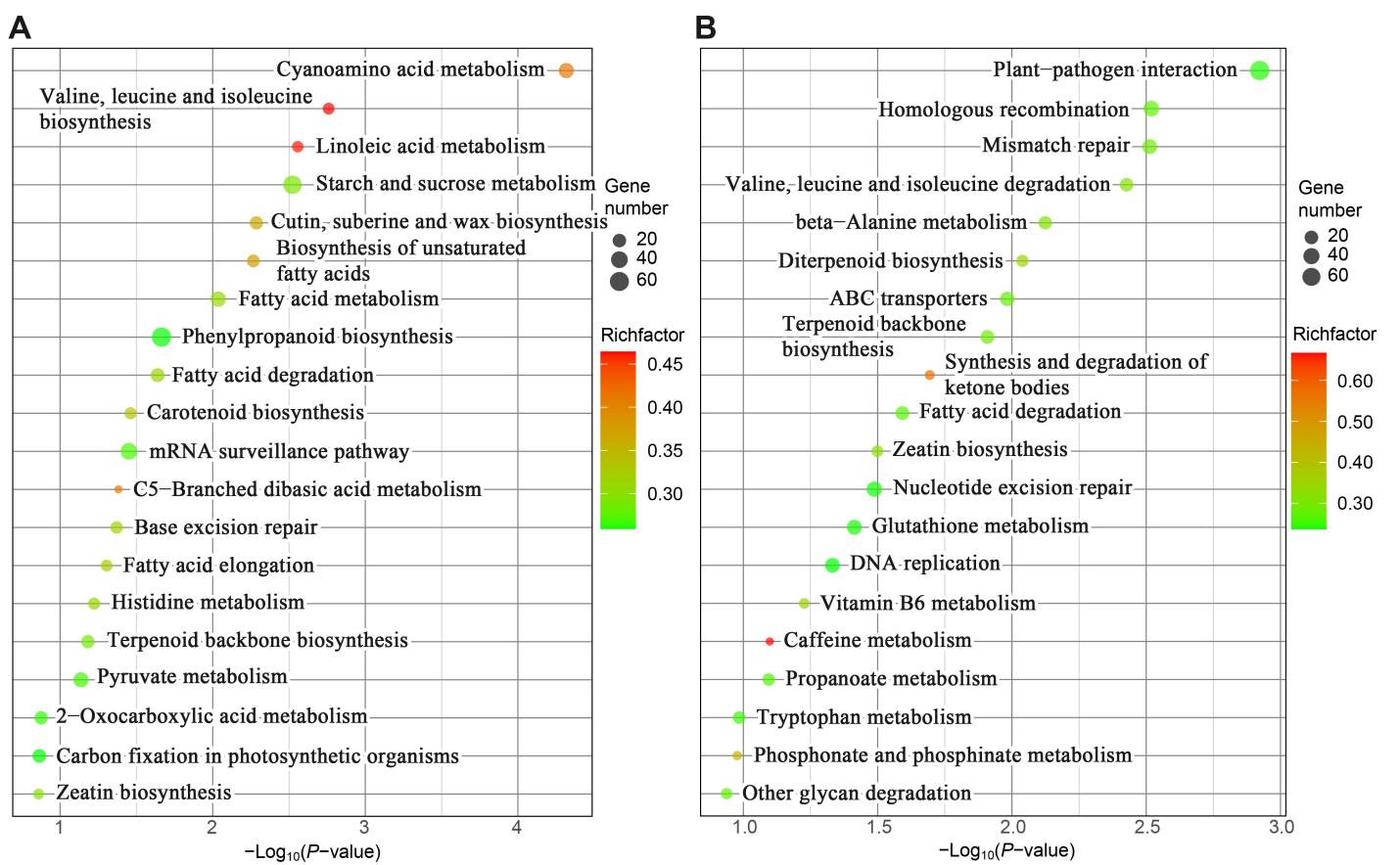


**Figure S29. KEGG pathway enrichment distribution of genes in the 1k downstream region of the unique LTRs of two rhododendrons.**

**(A)** *R. principis*. **(B)** *R. fortunei*.


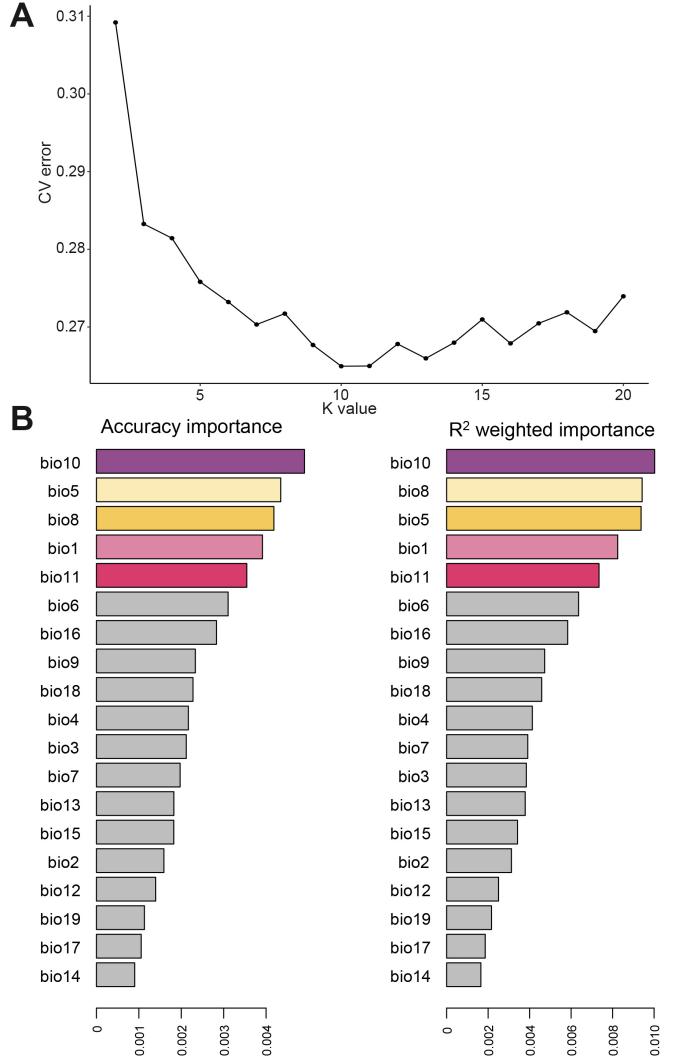


**Figure S30. Cross-validation (CV) error curves (A) and the order of importance of climatic factors (B).**


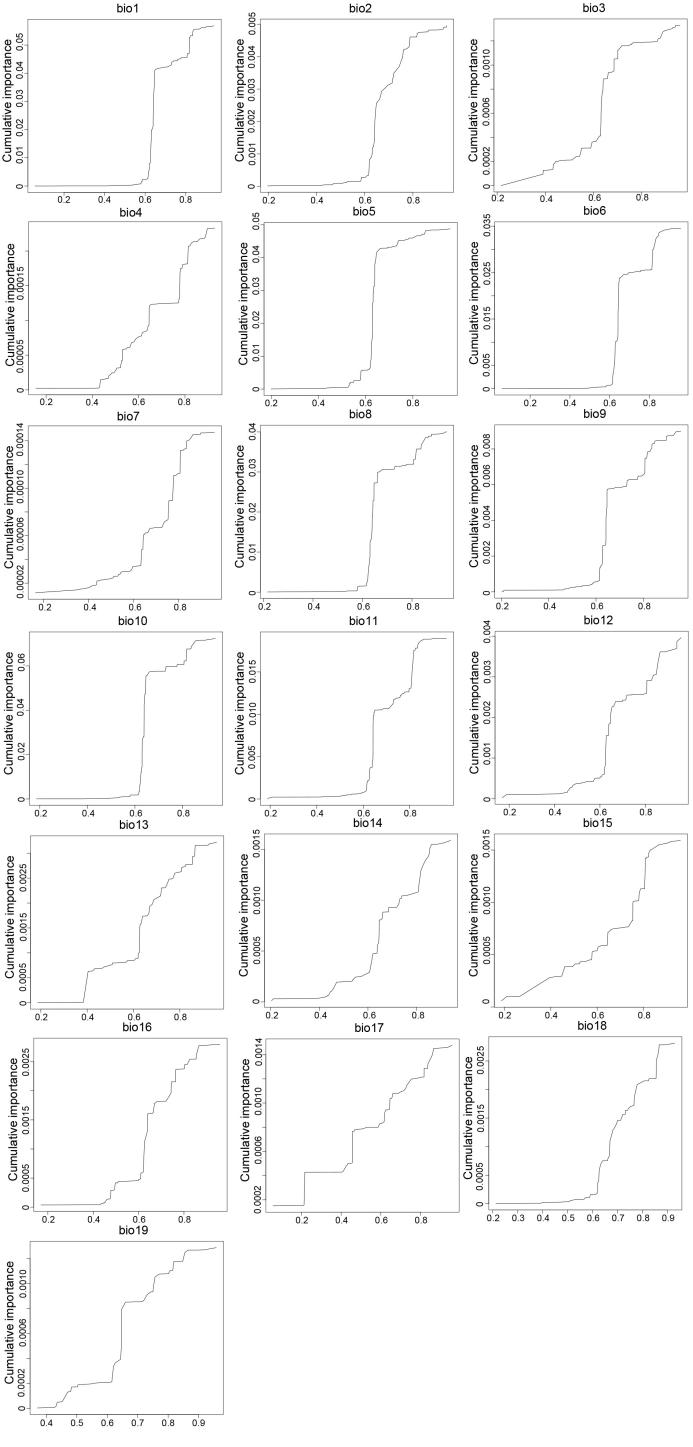


**Figure S31. Cumulative importance of allele changes on 19 environmental gradients.**


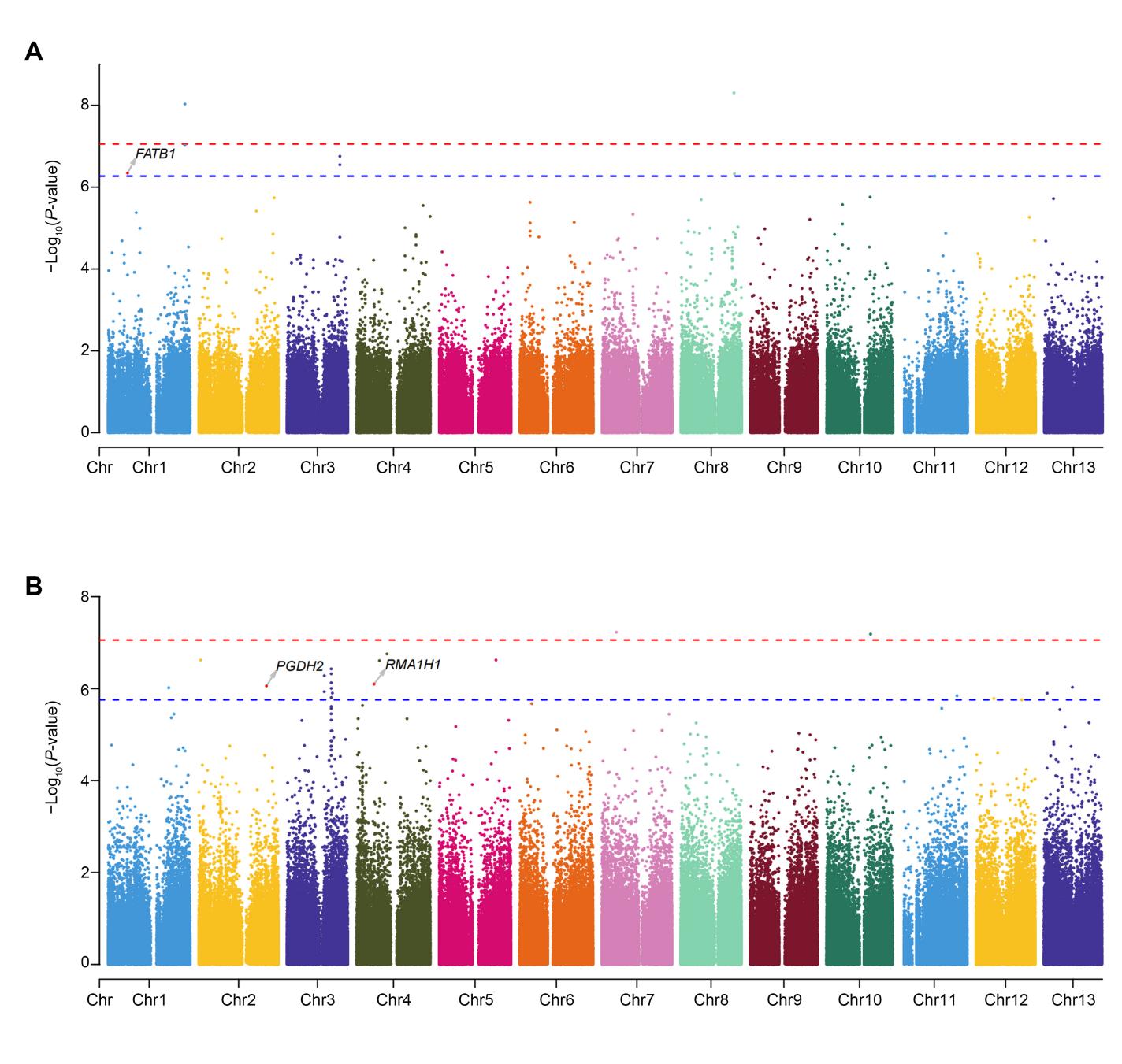


**Figure S32. Manhattan plot of LFMM for variants associated with BIO5 (A) and BIO11 (B).**

Dashed horizontal lines represent significance thresholds (blue dashed line represents the FDR correction, adjusted *P* = 0.05; red dashed line represents the Bonferroni correction model (0.05/N). Selected candidate genes are labeled in the plot at their respective genomic positions.


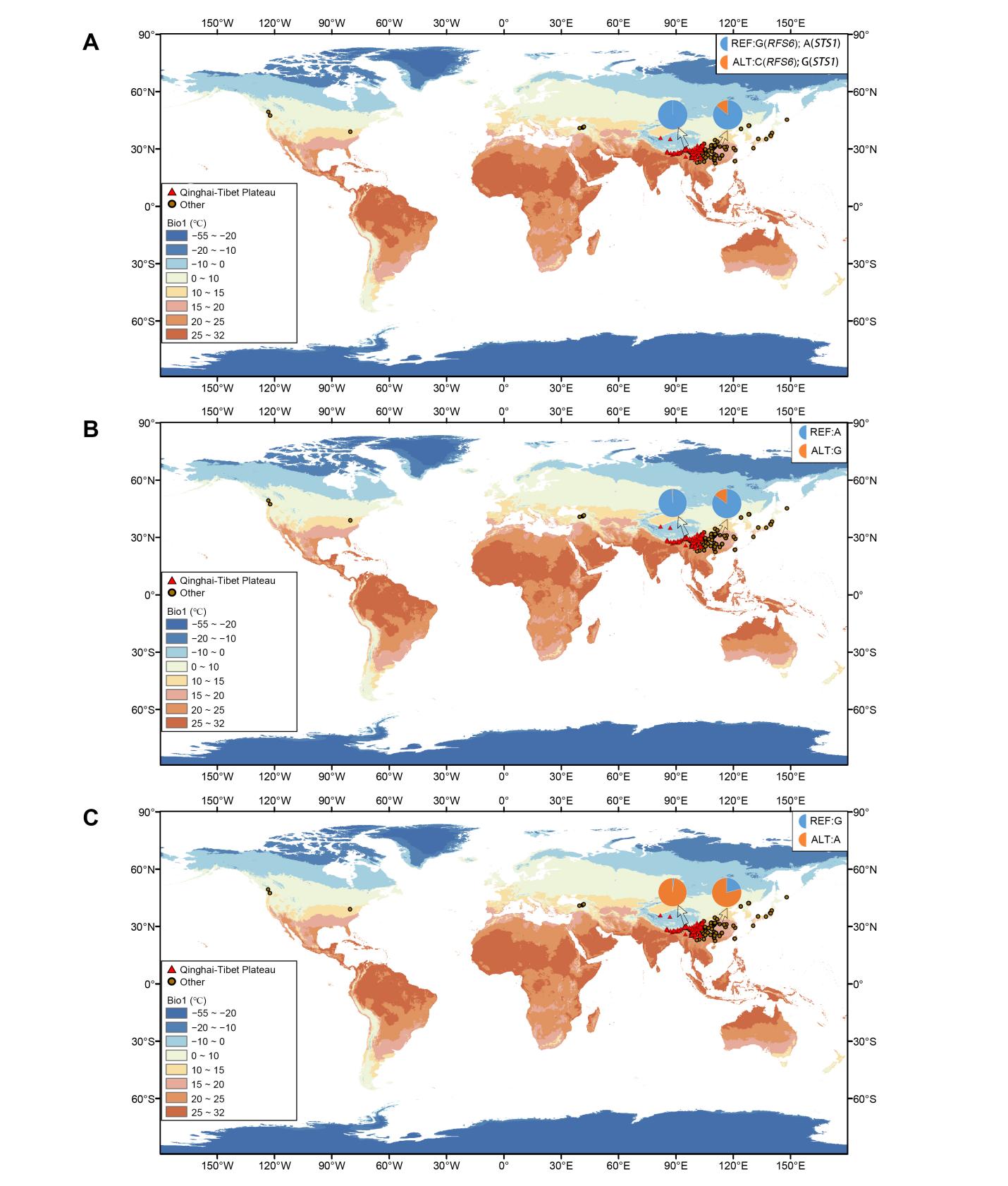


**Figure S33. Allele frequencies of candidate adaptive SNPs associated with BIO1 across the Qinghai-Tibet Plateau and other populations.**

Colors on the map are based on variations of the relevant climate variables across the distribution range. **(A)** *RFS6*, LG01:6852772; *STS1*, LG13:550414. **(B)** *PAO5*, LG03:32395546. **(C)** *LACS4*, LG07:629358. REF, reference allele type, consistent with the reference genome (no SNP variation on this site). ALT, alternate allele type (mutant type).


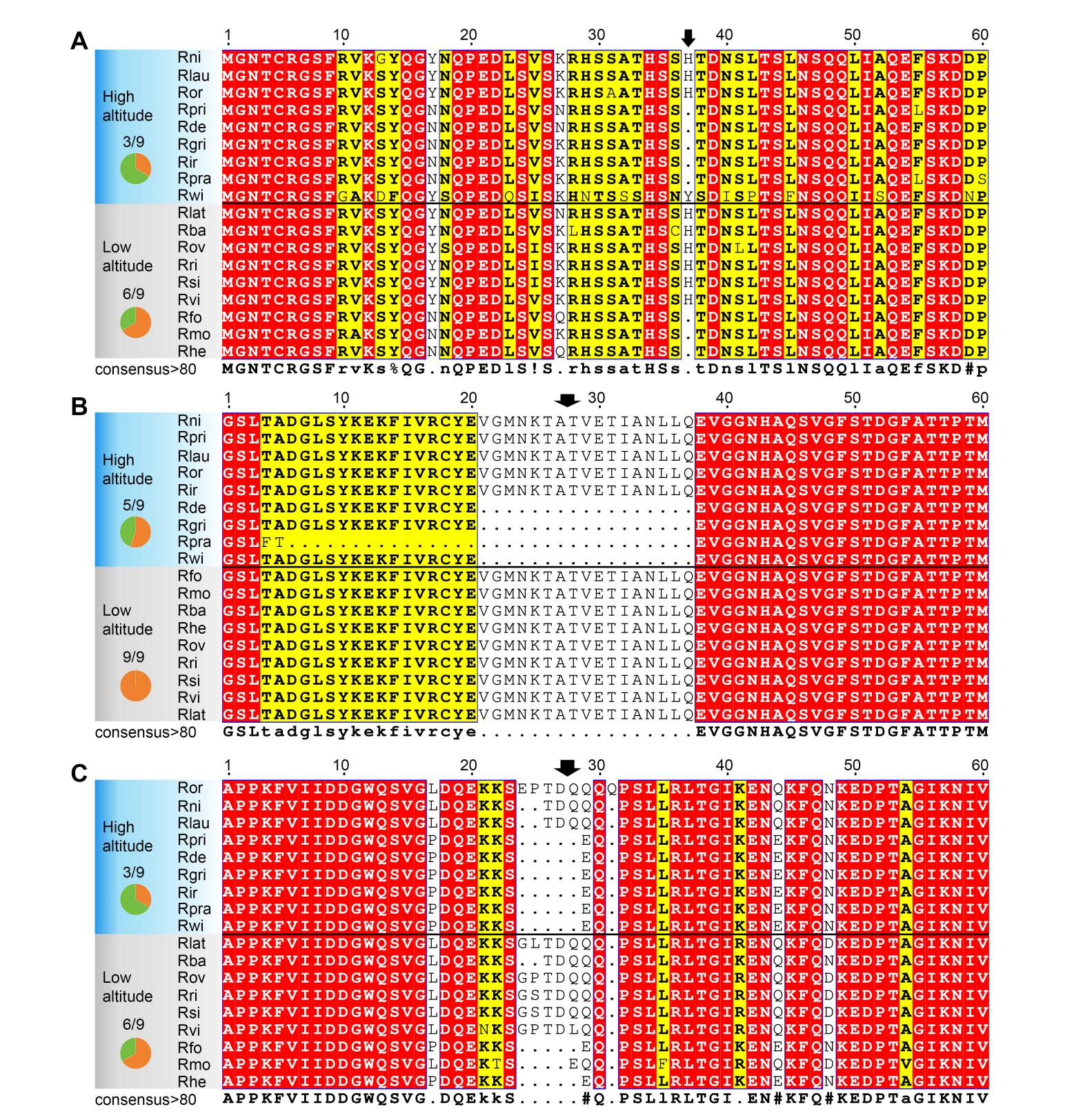


**Figure S34. Mutation status of candidate gene insertions or deletions.**

Indel differences of high-altitude adaptation related genes in different *Rhododendron* species. **(A)** *Solanum tuberosum* *CPK5* homologous gene. **(B)** *Coriandrum sativum* *FATA* homologous gene. **(C)** *RFS6* homologous gene of *Arabidopsis*. All displayed genes are CDS regions.


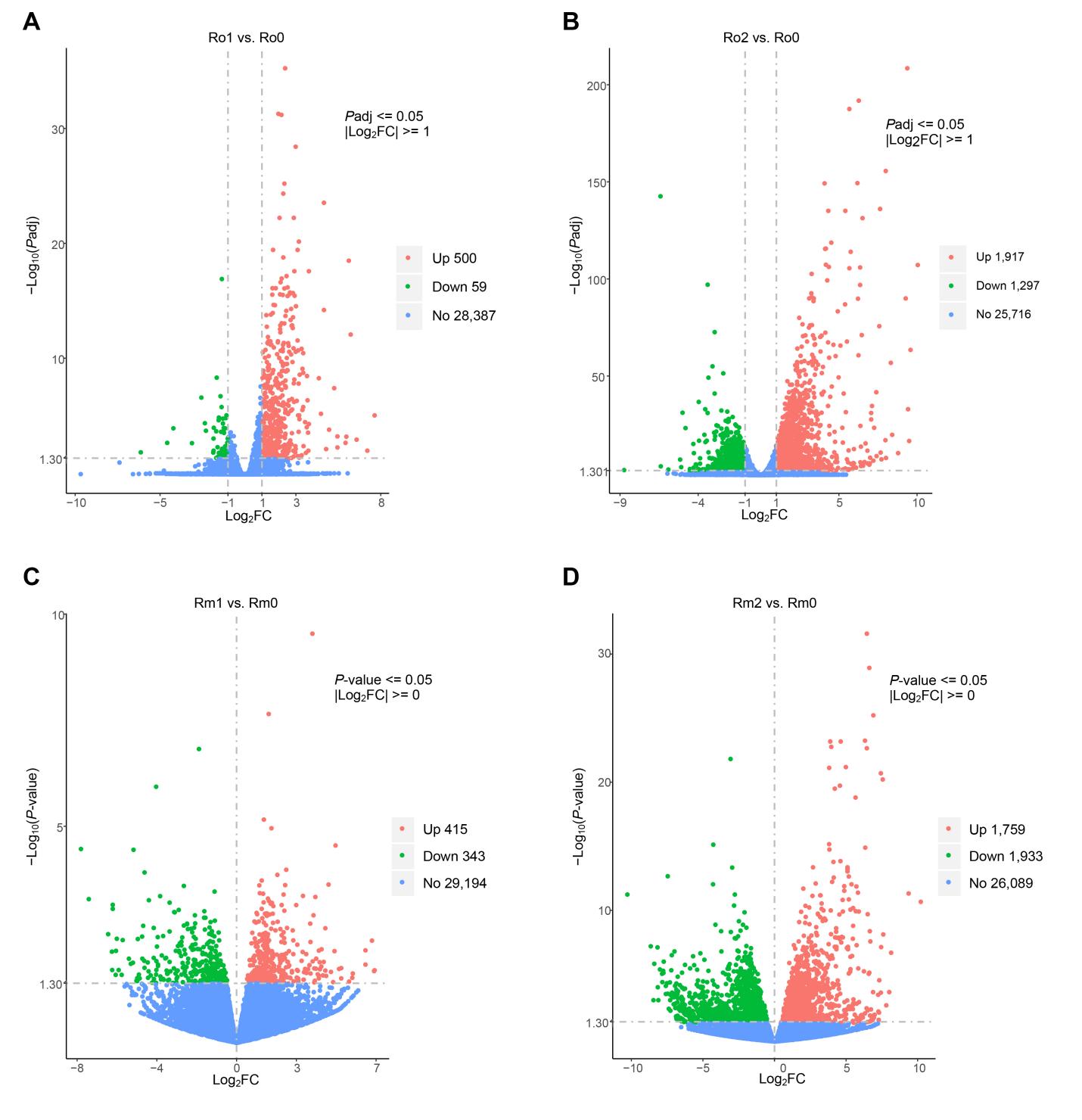


**Figure S35. Volcano plot of differentially expressed genes in *R. oreotrephes* and *R. molle* after 0.5 and 24 h of low-temperature stress.**

**(A**, **C)** Differentially expressed genes in *R. oreotrephes* (Ro)/ *R. molle* (Rm) at 0 and 0.5 hours of low temperature. **(B**, **D)** Differentially expressed genes in *R. oreotrephes* (Ro)/ *R. molle* (Rm) at 0 and 24 hours of low temperature.


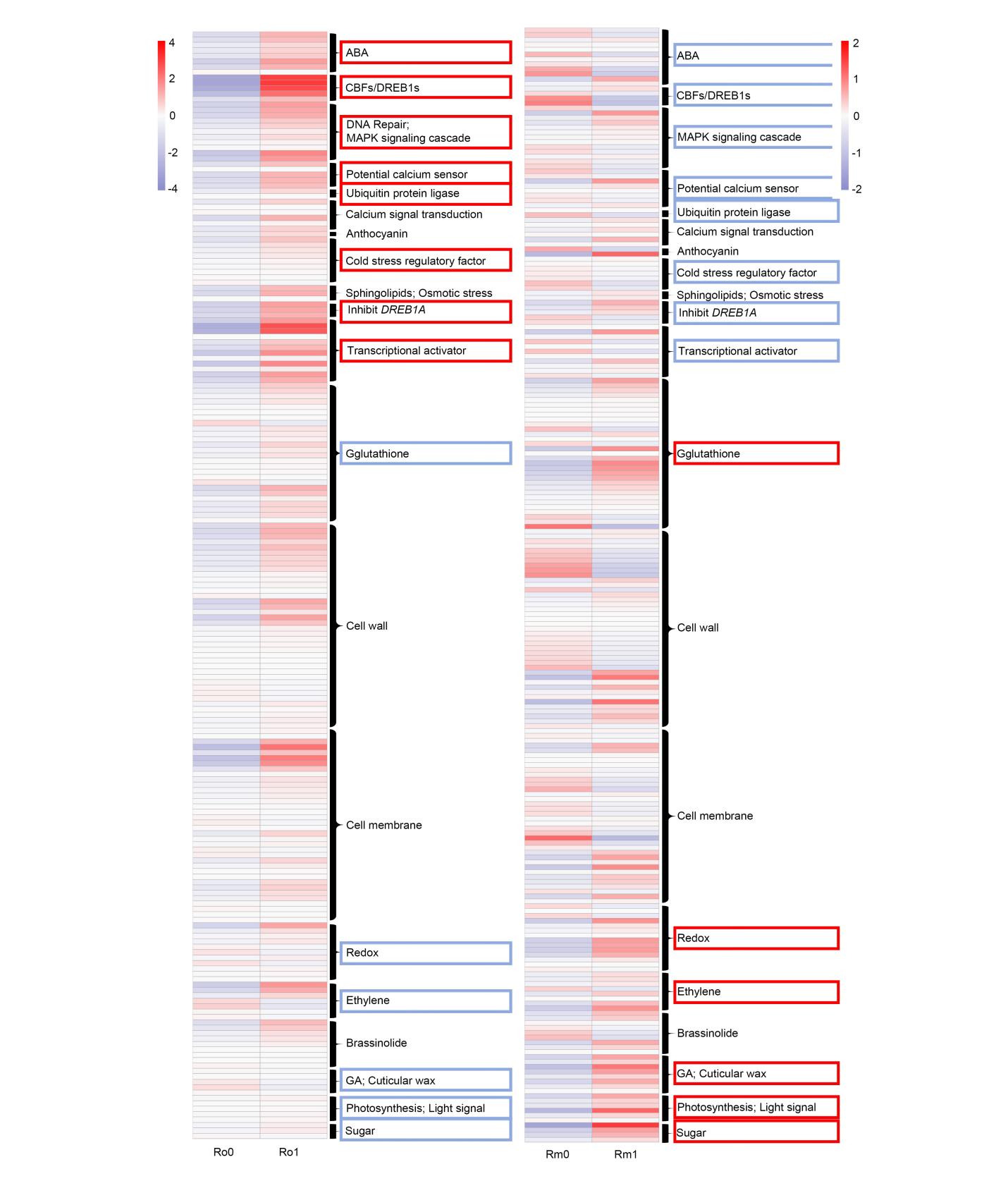


**Figure S36. Changes in the expression levels of cold resistance-related genes in *R. oreotrephes* (high-altitude) and *R. molle* (low-altitude) under 4℃ low-temperature treatment for 30 min.**

Ro0/ Rmo: *R. oreotrephes*/ *R. molle* at time 0 (before treatment); Ro1/ Rm1: *R. oreotrephes*/ *R. molle* at 30 min post-treatment. Red boxes indicate a significant up regulation of gene expression in this rhododendron compared to the other rhododendron for the pathway; blue boxes indicate no significant change or down regulation in expression.


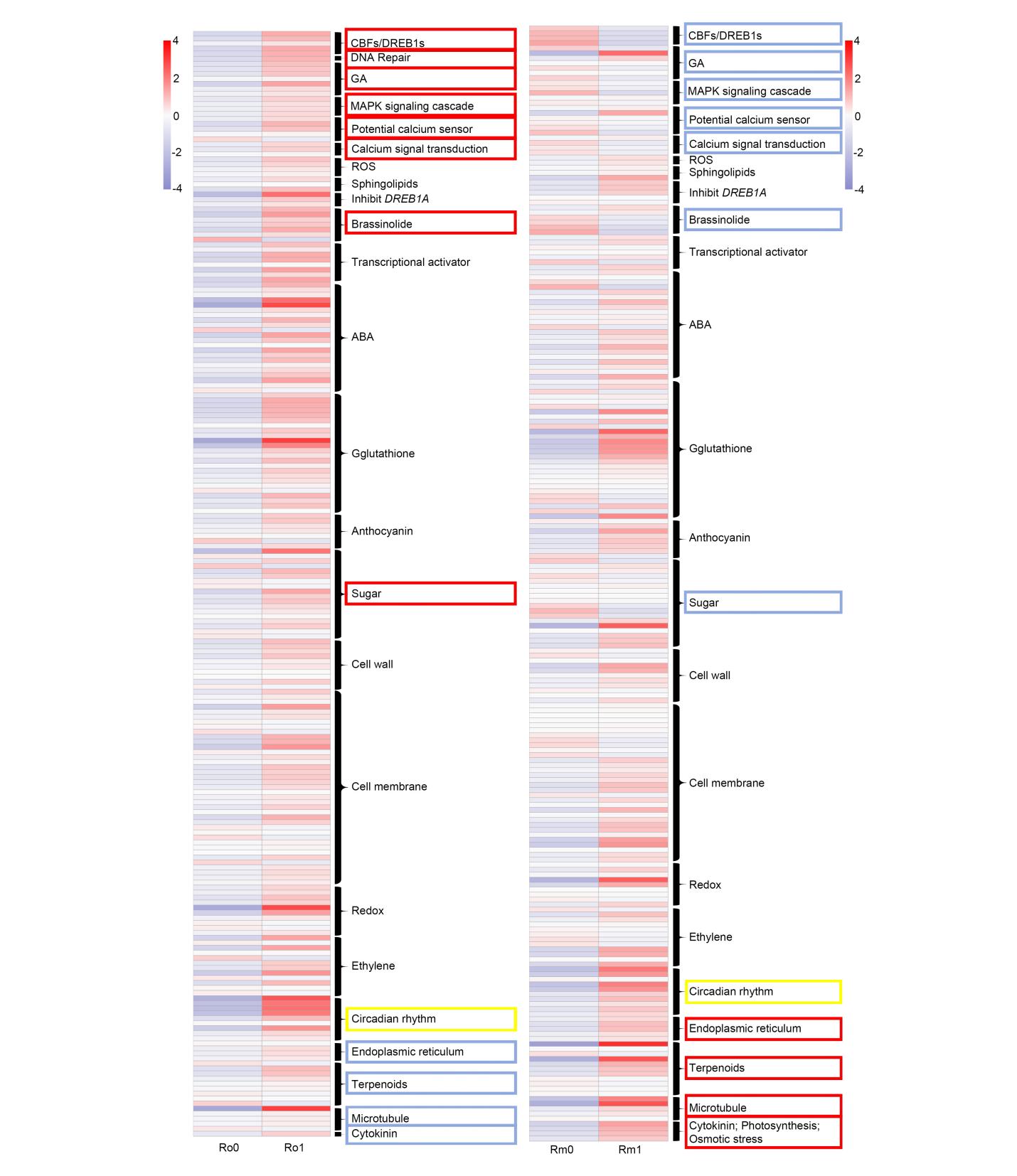


**Figure S37. Changes in the expression levels of cold resistance-related genes in *R. oreotrephes* (high-altitude) and *R. molle* (low-altitude) under 4℃ low-temperature treatment for 24 h.**

Ro0/ Rmo: *R. oreotrephes*/ *R. molle* at time 0 (before treatment); Ro1/ Rm1: *R. oreotrephes*/ *R. molle* at 24 hours post-treatment. Red boxes indicate a significant up regulation of gene expression in this rhododendron compared to the other rhododendron for the pathway; blue boxes indicate no significant change or down regulation in expression. Yellow boxes indicate newly added pathway genes that are significantly up regulated in both.


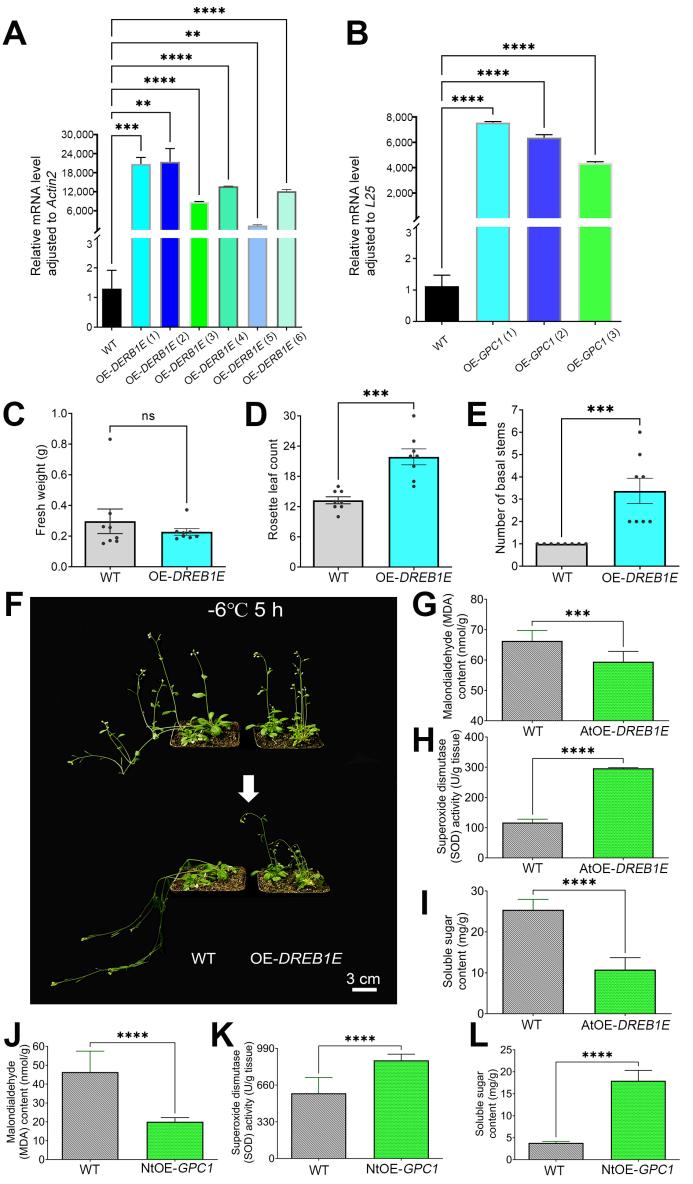


**Figure S38. Functional validation of cold resistance candidate genes.**

**(A)** Gene expression in *DREB1E* over expression T2 *Arabidopsis* seedlings. **(B)** Gene expression in *GPC1* over expression T1 flue-cured tobacco plants. **(C**–**E)** Differences in fresh weight, rosette leaf number, and basal stem number between wild-type and over expression T2 *Arabidopsis*. **(F)** Phenotypic changes of wild-type and *DREB1E* over expression T2 *Arabidopsis* plants after 5 h of −6°C freezing stress. **(G**–**I)** Differences in MDA content, SOD activity, and soluble sugar content between wild-type and *DREB1E* over expression T2 *Arabidopsis* 5 h of −6°C. **(J**–**L)** Differences in MDA content, SOD activity, and soluble sugar content between wild-type and *GPC1* over expression T1 flue-cured tobacco after 5 h of 4°C treatment.


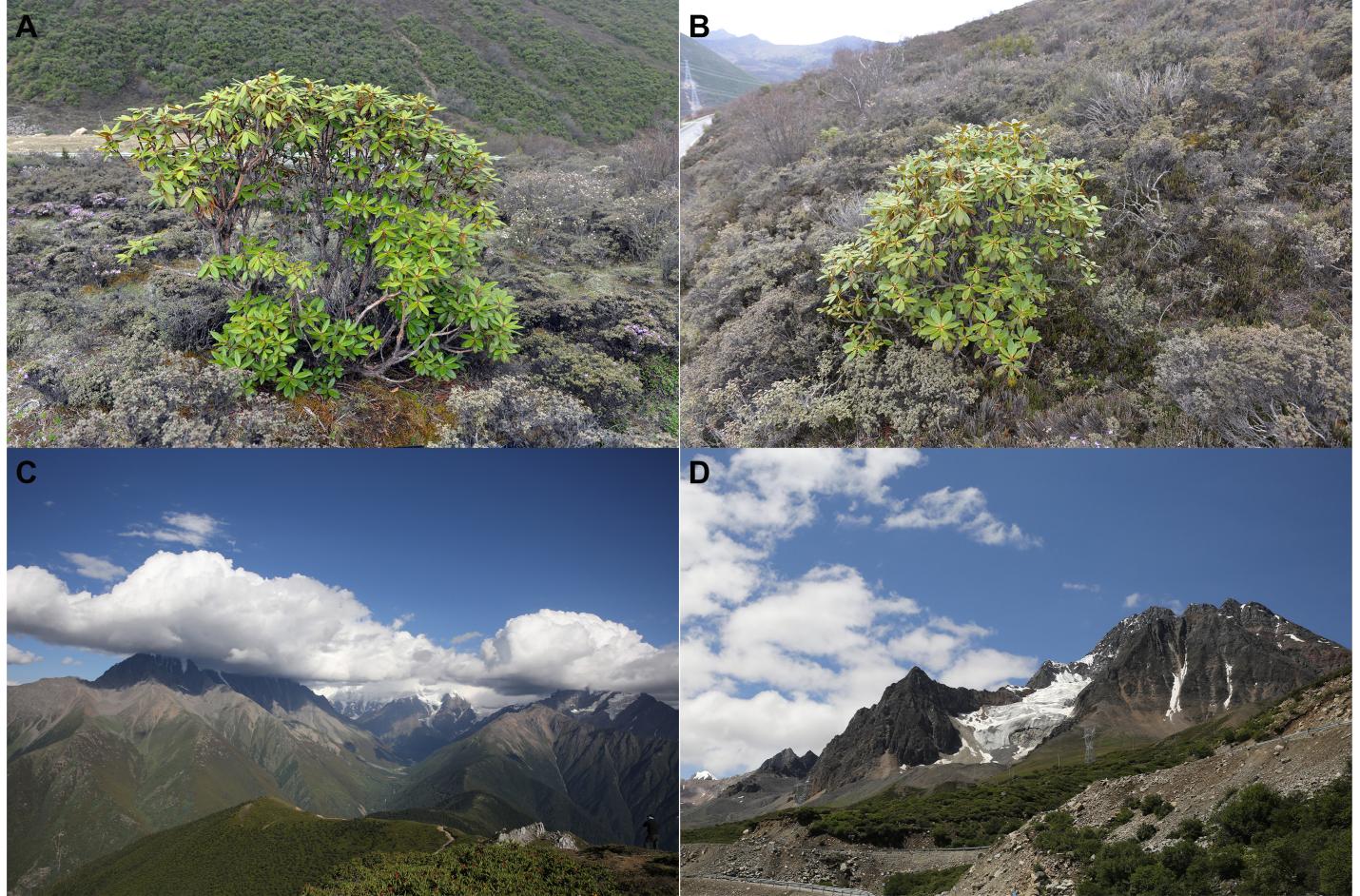


**Figure S39. Ecological environments of the high-altitude rhododendron community in Tibet.**

**(A**, **B)** Community of *R. nivale* (a few *R. principis* coexist with them). **(C**, **D)** Community of *R. laudandum* and *R. aganniphum* var*. flavorufum*.


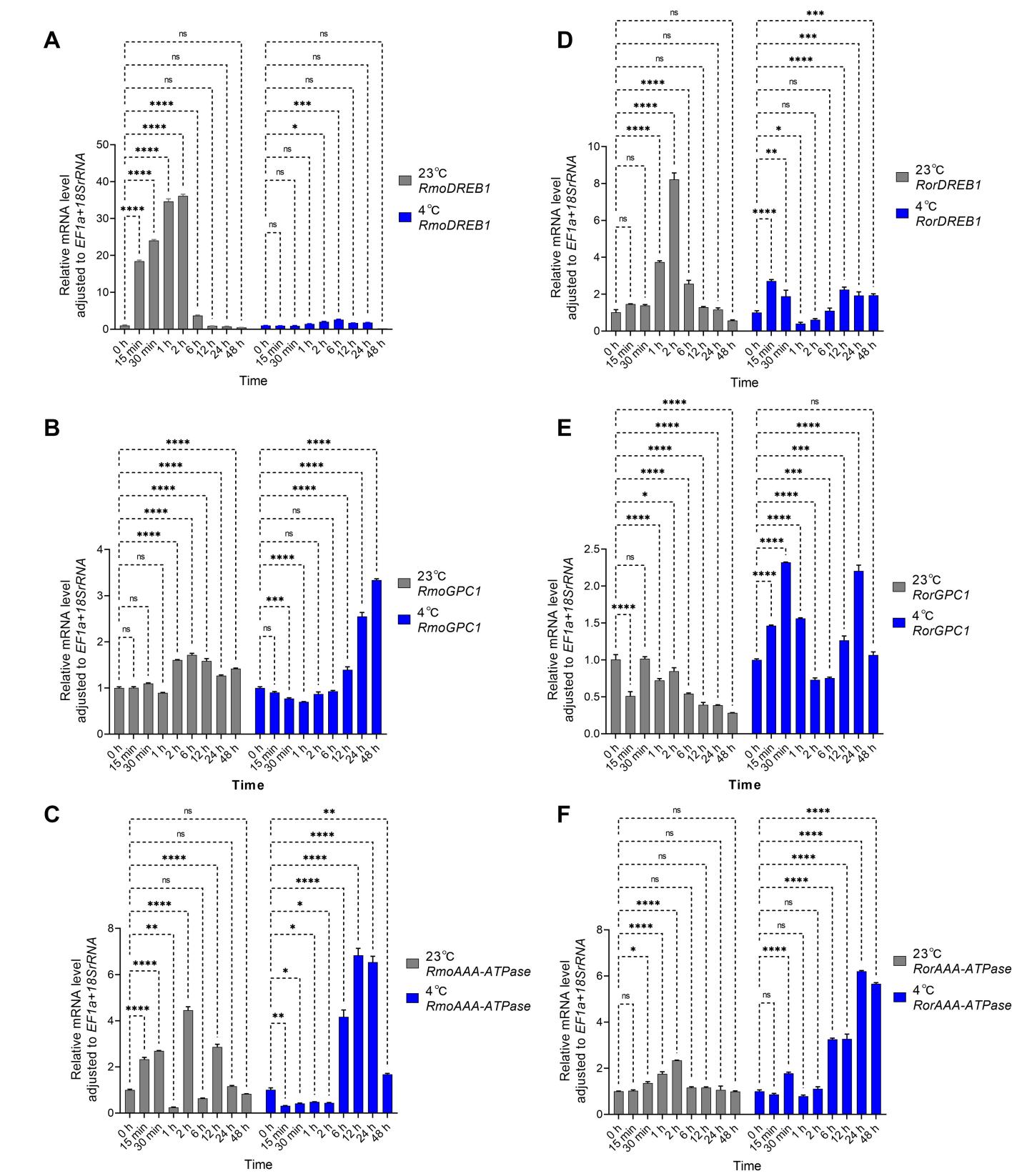


**Figure S40. qPCR analysis of *Rhododendron* species at different altitudes.**

**(A**–**C)** The trend of relative expression levels of three cold resistant genes in *R. molle* over time. **(D**–**F)** Relative expression level of three genes in *R. oreotrephes*. All data are represented as mean ± SEM.

**SUPPORTING TABLES**

**Table S1. Information of genome MGI sequencing**

| **Accession** | **Total reads** | **Total bases** | **Q20 rate(%)** | **Q30 rate(%)** | **GC(%)** | **remapped rate(%)** | **Coverage depth(X)** |
| --- | --- | --- | --- | --- | --- | --- | --- |
| DJ2 | 280,178,428 | 42,026,764,200 | 96.81 | 90.64 | 41.81 | 99.44 | 50.12 |
| DJ3 | 271,751,474 | 40,762,721,100 | 96.30 | 89.16 | 41.11 | 99.53 | 49.59 |
| DJ5 | 282,384,744 | 42,357,711,600 | 96.56 | 89.83 | 41.58 | 99.37 | 42.45 |
| LIU3693 | 280,157,270 | 42,023,590,500 | 97.21 | 91.37 | 41.78 | 98.81 | 46.21 |
| LIU3696 | 251,670,236 | 37,750,535,400 | 94.39 | 86.77 | 40.31 | 97.14 | 65.17 |
| LIU3697 | 328,370,976 | 49,255,646,400 | 97.39 | 91.89 | 41.81 | 99.43 | 65.47 |
| LIU3698 | 342,204,026 | 51,330,603,900 | 97.48 | 92.15 | 41.15 | 99.70 | 69.98 |

**Table S2. Information of genome PacBio sequencing**

| **Accession** | **Total pass reads bases** | **Total pass reads number** | **Pass reads mean length (bp)** | **Pass reads max length (bp)** | **Pass reads N50 length (bp)** | **Coverage depth(X)** |
| --- | --- | --- | --- | --- | --- | --- |
| DJ2 | 56,965,075,731 | 3,641,336 | 15,644 | 45,439 | 15,804 | 72.59 |
| DJ3 | 57,108,799,826 | 3,878,569 | 14,724 | 43,657 | 14,850 | 75.83 |
| DJ5 | 47,728,453,540 | 3,102,203 | 15,385 | 43,993 | 15,677 | 51.25 |
| LIU3693 | 30,088,828,103 | 2,176,437 | 13,824 | 46,550 | 13,664 | 35.67 |
| LIU3696 | 21,646,430,658 | 1,493,259 | 14,496 | 44,917 | 14,580 | 40.98 |
| LIU3697 | 32,111,104,988 | 2,049,754 | 15,665 | 49,837 | 16,016 | 45.86 |
| LIU3698 | 27,018,516,509 | 1,865,199 | 14,485 | 47,010 | 14,502 | 39.57 |

**Table S3. Information of Hi-C auxiliary assembly**

| **Accession** | Hi-C assembly genome size（bp） | **contig N50 (bp)** | **scaffold N50 (bp)** | **Estimated genome size (bp)** | **Proportion of actual assembly in estimated** | **Loading Rate** |
| --- | --- | --- | --- | --- | --- | --- |
| DJ2 | 622,921,387 | 26,000,000 | 45,013,818 | 614,088,494 | 101.44% | 95.36% |
| DJ3 | 617,959,644 | 24,300,000 | 47,248,519 | 597,738,488 | 103.38% | 97.88% |
| DJ5 | 602,446,664 | 30,626,833 | 43,713,038 | 602,971,223 | 99.91% | 96.99% |
| LIU3693 | 631,339,525 | 16,514,407 | 45,528,743 | 657,753,481 | 95.98% | 97.51% |
| LIU3696 | 489,173,567 | 32,399,153 | 36,947,515 | 549,182,038 | 89.07% | 97.90% |
| LIU3697 | 664,605,583 | 27,664,718 | 49,723,545 | 642,855,297 | 103.38% | 96.00% |
| LIU3698 | 650,252,595 | 10,561,241 | 51,653,293 | 633,614,951 | 102.63% | 97.93% |

**Table S4. Benchmarking universal single-copy orthologs of genome HiC assembly and annotation**

|  | **DJ2** | **DJ3** | **DJ5** | **LIU3693** | **LIU3696** | **LIU3697** | **LIU3698** |
| --- | --- | --- | --- | --- | --- | --- | --- |
| Assemble complete BUSCOs number/percent | 1,581/97.96% | 1,585/98.20% | 1,590/98.51% | 1,588/98.39% | 1,591/98.57% | 1,590/98.51% | 1,590/98.51% |
| Assemble complete and single-copy BUSCOs number/percent | 1,511/93.62% | 1,503/93.13% | 1,523/94.36% | 1,500/92.94% | 1,527/94.61% | 1,516/93.93% | 1,513/93.74% |
| Assemble complete and duplicated BUSCOs number/percent | 70/4.34% | 82/5.08% | 67/4.15% | 88/5.45% | 64/3.97% | 74/4.58% | 77/4.77% |
| Assemble fragmented BUSCOs number/percent | 12/0.74% | 13/0.81% | 9/0.56% | 12/0.74% | 7/0.43% | 12/0.74% | 10/0.62% |
| Assemble missing BUSCOs number/percent | 21/1.30% | 16/0.99% | 15/0.93% | 14/0.87% | 16/0.99% | 12/0.74% | 14/0.87% |
| Annotation complete BUSCOs number/percent | 1,546/95.79% | 1,561/96.72% | 1,535/95.11% | 1,532/94.92% | 1,572/97.40% | 1,559/96.59% | 1,547/95.85% |
| Annotation complete and single-copy BUSCOs number/percent | 1,482/91.82% | 1,494/92.57% | 1,470/91.08% | 1,462/90.58% | 1,514/93.80% | 1,495/92.63% | 1,484/91.95% |
| Annotation complete and duplicated BUSCOs number/percent | 64/3.97% | 67/4.15% | 65/4.03% | 70/4.34% | 58/3.59% | 64/3.97% | 63/3.90% |
| Annotation fragmented BUSCOs number/percent | 11/0.68% | 13/0.81% | 17/1.05% | 17/1.05% | 9/0.56% | 11/0.68% | 12/0.74% |
| Annotation missing BUSCOs number/percent | 57/3.53% | 40/2.48% | 62/3.84% | 65/4.03% | 33/2.04% | 44/2.73% | 55/3.41% |

**Table S5. The predicted coding protein gene information of in genome**

| **Species** | **Genome total length（bp）** | **Total number of coding genes** | **Average intron length(bp)** | **Average exon length(bp)** | **Average CDS length(bp)** | **Average gene length(bp)** |
| --- | --- | --- | --- | --- | --- | --- |
| *R. nivale* | 622,921,387 | 33,932 | 1,924.08 | 217.36 | 1,107.42 | 8,986.16 |
| *R. principis* | 617,959,644 | 35,451 | 1,865.63 | 221.11 | 1,135.11 | 8,847.14 |
| *R. laudandum* | 602,446,664 | 34,352 | 1,862.79 | 218.07 | 1,115.70 | 8,783.61 |
| *R. oreotrephes* | 631,339,525 | 34,236 | 1,937.44 | 217.58 | 1,119.46 | 9,150.1 |
| *R. latoucheae* | 489,173,567 | 33,438 | 1,557.26 | 223.1 | 1,148.01 | 7,603.99 |
| *R. fortunei* | 664,605,583 | 37,399 | 1,783.34 | 220.63 | 1,113.64 | 8,331.93 |
| *R. molle* | 650,252,595 | 36,855 | 1,803.87 | 216.28 | 1,085.05 | 8,331.18 |

**Table S6. Information of genomic transposable elements**

| **Species** | **LTR (bp/%)** | **LINE (bp/%)** | **SINE (bp/%)** | **DNA (bp/%)** | **Total TEs (bp/%)** | **Total repeats (bp/%)** |
| --- | --- | --- | --- | --- | --- | --- |
| *R. nivale* | 195,743,234/31.42 | 32,526,152/5.22 | 7,356,946/1.18 | 44,230,955/7.10 | 279,857,287/44.93 | 376,338,239/60.42 |
| *R. principis* | 176,695,880/28.59 | 21,407,238/3.46 | 9,282,935/1.50 | 42,642,386/6.90 | 250,028,439/40.46 | 351,903,320/56.95 |
| *R. laudandum* | 185,401,864/30.77 | 27,209,265/4.52 | 8,025,470/1.33 | 40,213,374/6.68 | 260,849,973/43.30 | 354,303,120/58.81 |
| *R. oreotrephes* | 202,228,488/32.03 | 20,371,544/3.23 | 12,275,135/1.94 | 44,048,001/6.98 | 278,923,168/44.18 | 381,004,915/60.35 |
| *R. latoucheae* | 121,448,263/24.83 | 12,655,622/2.59 | 8,320,199/1.70 | 35,518,412/7.26 | 177,942,496/36.38 | 252,826,788/51.68 |
| *R. fortunei* | 206,608,066/31.09 | 21,685,084/3.26 | 11,710,781/1.76 | 41,126,854/6.19 | 281,130,785/42.30 | 391,665,728/58.93 |
| *R. molle* | 229,130,024/35.24 | 14,542,352/2.24 | 10,142,808/1.56 | 47,589,515/7.32 | 301,404,699/46.35 | 394,191,434/60.62 |
| *R. delavayi* | 211,101,983/31.79 | 25,620,464/3.86 | 10,527,664/1.59 | 47,052,634/7.09 | 294,302,745/44.32 | 392,225,230/59.07 |
| *R. griersonianum* | 241,514,254/35.78 | 25,276,460/3.74 | 15,000,877/2.22 | 46,676,192/6.92 | 328,467,783/48.66 | 407,516,586/60.37 |
| R. henanense subsp. lingbaoense | 196,481,852/30.98 | 21,705,783/3.42 | 9,596,044/1.51 | 42,491,883/6.70 | 270,275,562/42.61 | 366,743,647/57.82 |
| *R. ovatum* | 127,421,648/23.18 | 13,570,312/2.47 | 10,751,073/1.96 | 41,056,697/7.47 | 192,799,730/35.07 | 284,723,585/51.80 |
| *R. ripense* | 108,415,912/21.40 | 15,083,779/2.98 | 12,257,474/2.42 | 35,953,993/7.10 | 171,711,158/33.89 | 259,337,005/51.18 |
| *R. simsii* | 111,850,838/21.16 | 15,175,765/2.87 | 10,341,338/1.96 | 41,565,722/7.86 | 178,933,663/33.85 | 271,917,030/51.44 |
| *R. williamsianum* | 34,335,196/6.45 | 9,734,863/1.83 | 5,241,724/0.98 | 20,437,328/3.84 | 69,749,111/13.10 | 153,088,334/28.76 |
| *R. bailiense* | 397,320,273/42.99 | 25,677,965/2.78 | 13,371,710/1.45 | 80,586,293/8.72 | 516,956,241/55.94 | 613,011,169/66.33 |
| *R. irroratum* | 190,032,470/27.09 | 24,360,538/3.47 | 12,659,172/1.80 | 51,228,379/7.30 | 278,280,559/39.66 | 397,439,149/56.65 |
| *R. prattii* | 18,3740,317/27.30 | 25,417,964/3.78 | 15,643,482/2.32 | 49,506,541/7.36 | 274,308,304/40.75 | 382,520,341/56.83 |
| *R. vialii* | 133,376,612/25.04 | 13,265,668/2.49 | 11,795,938/2.21 | 68,074,142/12.78 | 226,512,360/42.52 | 298,463,582/56.02 |

**Table S7. Information of genomic non-coding RNAs**

| **Accession** | **rRNA number** | **Small RNA number** | **Regulatory number** | **tRNA number** |
| --- | --- | --- | --- | --- |
| DJ2 | 4,454 | 1,382 | 11 | 507 |
| DJ3 | 2,970 | 1,512 | 15 | 512 |
| DJ5 | 2,959 | 1,427 | 5 | 723 |
| LIU3693 | 1,883 | 1,475 | 8 | 518 |
| LIU3696 | 1,723 | 1,316 | 13 | 481 |
| LIU3697 | 3,633 | 1,493 | 14 | 492 |
| LIU3698 | 2,479 | 1,713 | 12 | 503 |

**Table S8. Information of pan-genome gene family clustering**

| **Frequency** | **Group** | **Family number** |
| --- | --- | --- |
| 1 | Private | 4,899 |
| 2 | Dispensable | 11,310 |
| 3 | Dispensable | 4,535 |
| 4 | Dispensable | 2,662 |
| 5 | Dispensable | 1,687 |
| 6 | Dispensable | 1,181 |
| 7 | Dispensable | 965 |
| 8 | Dispensable | 730 |
| 9 | Dispensable | 647 |
| 10 | Dispensable | 602 |
| 11 | Dispensable | 572 |
| 12 | Dispensable | 623 |
| 13 | Dispensable | 718 |
| 14 | Dispensable | 780 |
| 15 | Dispensable | 1,144 |
| 16 | Softcore | 1,902 |
| 17 | Softcore | 3,596 |
| 18 | Core | 7,113 |

**Table S9. Summary of identified insertions, presence and absence variation**

| **Species** | **Presence number** | **Presence length (bp)** | **Absence number** | **Absence length (bp)** | **Inversion number** | **Inversion length (bp)** |
| --- | --- | --- | --- | --- | --- | --- |
| *R. nivale* | 6,922 | 40,078,603 | 6,609 | 35,063,338 | 518 | 1,989,811 |
| *R. principis* | 7,025 | 38,999,918 | 7,489 | 39,835,214 | 462 | 1,945,228 |
| *R. laudandum* | 7,000 | 40,097,005 | 6,650 | 35,110,921 | 1,171 | 4,693,881 |
| *R. oreotrephes* | 7,253 | 42,128,556 | 6,772 | 35,031,938 | 116 | 495,292 |
| *R. latoucheae* | 7,097 | 39,141,642 | 7,964 | 44,104,683 | 502 | 2,308,560 |
| 1. *fortunei* | 7,116 | 40,209,533 | 7,375 | 39,058,067 | 133 | 560,274 |
| *R. molle* | 6,459 | 38,506,628 | 5,746 | 31,390,036 | 298 | 1,174,283 |
| *R. delavayi* | 7,523 | 42,402,561 | 7,042 | 37,137,140 | 233 | 783,247 |
| *R. griersonianum* | 7,445 | 42,595,279 | 6,888 | 35,410,730 | 211 | 694,083 |
| R. henanense subsp. lingbaoense | 6,787 | 37,298,512 | 7,306 | 39,570,907 | 649 | 2,828,856 |
| *R. ovatum* | 12,813 | 47,742,885 | 17,390 | 75,735,420 | 883 | 5,890,494 |
| *R. ripense* | 7,087 | 39,543,440 | 6,685 | 37,099,454 | 743 | 3,106,519 |
| *R. simsii* | 7,059 | 38,838,176 | 6,828 | 38,142,057 | 539 | 1,942,136 |
| *R. williamsianum* | 5,902 | 30,985,210 | 7,250 | 39,545,654 | 1,924 | 7,774,773 |
| *R. irroratum* | 6,756 | 37,976,265 | 7,026 | 37,542,457 | 187 | 848,232 |
| *R. bailiense* | 7,009 | 41,838,693 | 5,355 | 26,910,844 | 356 | 1,397,713 |
| *R. prattii* | 6,641 | 36,590,152 | 7,160 | 38,370,194 | 75 | 290,009 |

**Table S10. Summary of identified intra-chromosomal and inter-chromosomal translocations**

| **Species** | **Intra-chromosome number** | **Intra-chromosome length (bp)** | **Inter-chromosome number** | **Inter-chromosome length (bp)** |
| --- | --- | --- | --- | --- |
| *R. nivale* | 30 | 290,100 | 81 | 540,334 |
| *R. principis* | 37 | 344,029 | 91 | 607,653 |
| *R. laudandum* | 35 | 334,019 | 87 | 645,199 |
| *R. oreotrephes* | 36 | 283,843 | 91 | 659,044 |
| *R. latoucheae* | 30 | 265,082 | 88 | 640,143 |
| *R. fortunei* | 37 | 247,593 | 86 | 531,747 |
| *R. molle* | 15 | 70,778 | 100 | 729,011 |
| *R. delavayi* | 20 | 101,663 | 95 | 617,080 |
| *R. griersonianum* | 25 | 109,926 | 90 | 581,058 |
| R. henanense subsp. lingbaoense | 39 | 305,281 | 101 | 718,368 |
| *R. ovatum* | 47 | 398,558 | 229 | 2,324,056 |
| *R. ripense* | 30 | 211,990 | 89 | 628,949 |
| *R. simsii* | 23 | 118,583 | 87 | 568,552 |
| *R. williamsianum* | 10 | 50,787 | 83 | 603,653 |
| *R. irroratum* | 41 | 212,043 | 111 | 748,484 |
| *R. bailiense* | 37 | 257,386 | 125 | 910,349 |
| *R. prattii* | 35 | 197,717 | 118 | 742,367 |

**Table S11. Summary of identified copy number variants**

| **Species** | **CNV number** | **CNV length (bp)** |
| --- | --- | --- |
| *R. nivale* | 165 | 321,787 |
| *R. principis* | 276 | 640,165 |
| *R. laudandum* | 208 | 440,322 |
| *R. oreotrephes* | 256 | 653,076 |
| *R. latoucheae* | 202 | 465,818 |
| *R. fortunei* | 340 | 859,564 |
| *R. molle* | 161 | 378,456 |
| *R. delavayi* | 453 | 1,367,878 |
| *R. griersonianum* | 420 | 1,308,839 |
| R. henanense subsp. lingbaoense | 391 | 1,194,418 |
| *R. ovatum* | 1,700 | 9,545,145 |
| *R. ripense* | 274 | 822,323 |
| *R. simsii* | 350 | 1,073,369 |
| *R. williamsianum* | 85 | 65,440 |
| *R. irroratum* | 649 | 2,520,761 |
| *R. bailiense* | 356 | 1,162,800 |
| *R. prattii* | 498 | 1,745,055 |

**Table S12. Information of SVs as the number of samples changes**

| **Number of samples added** | **Core SVs** | **Pan SVs** |
| --- | --- | --- |
| 1 | 14,822 | 0 |
| 2 | 6,540 | 16,771 |
| 3 | 4,798 | 22,400 |
| 4 | 3,213 | 29,182 |
| 5 | 2,687 | 32,113 |
| 6 | 2,241 | 34,232 |
| 7 | 1,824 | 37,429 |
| 8 | 1,315 | 40,294 |
| 9 | 694 | 60,361 |
| 10 | 644 | 61,334 |
| 11 | 569 | 62,664 |
| 12 | 447 | 65,263 |
| 13 | 383 | 66,853 |
| 14 | 353 | 67,716 |
| 15 | 326 | 68,788 |
| 16 | 263 | 70,066 |
| 17 | 240 | 71,849 |

**Table S13. Information on SVs classification of different rhododendrons**

| **Species** | **Core/percent** | **Softcore/percent** | **Dispensable/percent** | **Private/percent** | **Summary** |
| --- | --- | --- | --- | --- | --- |
| *R. nivale* | 266/0.89% | 1,804/6.00% | 13,661/45.45% | 14,325/47.66% | 30,056/100% |
| *R. principis* | 281/0.87% | 1,866/5.77% | 14,794/45.77% | 15,380/47.59% | 32,321/100% |
| *R. laudandum* | 268/0.86% | 1,808/5.77% | 14,097/45.00% | 15,151/48.37% | 31,324/100% |
| *R. oreotrephes* | 272/0.91% | 1,776/5.92% | 13,453/44.81% | 14,524/48.37% | 30,025/100% |
| *R. latoucheae* | 267/0.84% | 1,729/5.42% | 14,000/43.92% | 15,883/49.82% | 31,879/100% |
| *R. fortunei* | 278/0.87% | 1,864/5.86% | 14,566/45.81% | 15,087/47.45% | 31,795/100% |
| *R. molle* | 259/0.99% | 1,639/6.28% | 11,427/43.77% | 12,779/48.95% | 26,104/100% |
| *R. delavayi* | 280/0.88% | 1,821/5.71% | 14,409/45.20% | 15,366/48.21% | 31,876/100% |
| *R. griersonianum* | 283/0.90% | 1,850/5.87% | 14,291/45.36% | 15,079/47.87% | 31,503/100% |
| *R. henanense* subsp*. lingbaoense* | 285/0.89% | 1,866/5.85% | 14,458/45.35% | 15,273/47.90% | 31,882/100% |
| *R. ovatum* | 276/0.55% | 1,322/2.65% | 15,241/30.54% | 33,062/66.26% | 49,901/100% |
| *R. ripense* | 240/0.79% | 1,623/5.35% | 13,587/44.76% | 14,908/49.11% | 30,358/100% |
| *R. simsii* | 264/0.88% | 1,696/5.64% | 13,214/43.96% | 14,886/49.52% | 30,060/100% |
| *R. williamsianum* | 276/0.90% | 1,796/5.83% | 13,494/43.78% | 15,254/49.49% | 30,820/100% |
| *R. bailiense* | 267/0.98% | 1,679/6.17% | 12,037/44.22% | 13,238/48.63% | 27,221/100% |
| *R. irroratum* | 282/0.92% | 1,790/5.84% | 13,832/45.09% | 14,770/48.15% | 30,674/100% |
| *R. prattii* | 277/0.91% | 1,800/5.92% | 13,777/45.35% | 14,527/47.82% | 30,381/100% |

**Table S14. Significant gene information screened by different analysis**

| **Gene ID** | **Coding protein name** | **Screening method** |
| --- | --- | --- |
| XP_058225642.1 | *TPPB* | LFMM, *F*st |
| XP_058225634.1 | *LACS4* | LFMM, *F*st |
| XP_058222812.1/  evm_TU_LG06_358 | *KCS4* | Transcriptome, LFMM |
| XP_058193791.1/  evm_TU_LG11_188 | *RFS* | Transcriptome, LFMM |
| XP_058206621.1/  evm_TU_LG09_378 | *PAO5* | Transcriptome, LFMM |
| XP_058198632.1/  evm_TU_LG08_2654 | *STS1* | Transcriptome, LFMM |
| XP_058215615.1/  evm_TU_LG03_2328 | *NCER1* | Transcriptome, LFMM |
| XP_058212669.1/  evm_TU_LG02_1124 | *RFS2* | Transcriptome, LFMM |
| XP_058209039.1/  evm_TU_LG02_770 | *RMA1H1* | Transcriptome, LFMM |
| XP_058183036.1/  evm_TU_LG10_959 | *CML16* | Transcriptome, LFMM |
| XP_058210270.1/  evm_TU_LG02_489 | *MPK19* | Transcriptome, LFMM |
| XP_058198087.1/  evm_TU_LG08_1385 | *GST3* | Transcriptome, *F*st |
| XP_058207998.1/  evm_TU_LG09_1863 | *CPK1* | Transcriptome, *F*st |
| XP_058227053.1/  evm_TU_LG11_810 | *CML18* | Transcriptome, *F*st |
| XP_058225729.1/  evm_TU_LG07_105 | *sld1* | Transcriptome, *F*st |
| XP_058223947.1 | *LPAT2* | LFMM |
| XP_058199597.1 | *SUS6* | LFMM |
| XP_058181785.1 | *CYT1* | LFMM |
| XP_058205018.1 | *INV*DC4* | LFMM |
| XP_058224893.1 | *ACC1* | LFMM |
| XP_058205964.1 | *YDA* | LFMM |
| XP_058218967.1 | *RFS1* | LFMM |
| XP_058182010.1 | *RFS6* | LFMM |
| XP_058196475.1 | *FATA* | LFMM |
| XP_058220714.1 | *DREB1F* | LFMM |
| XP_058180602.1 | *CPK17* | LFMM |
| XP_058213804.1 | *CPK5* | LFMM |
| XP_058227645.1 | *CPK22* | LFMM |
| XP_058184056.1 | *FATB1* | LFMM |
| XP_058203313.1 | *PGDH2* | LFMM |
| XP_058182211.1 | *MPK14* | LFMM |
| XP_058181579.1 | *CPK20* | *F*st |
| XP_058187910.1 | *UVR8* | *F*st |
| XP_058179311.1 | *LIG6* | *F*st |
| XP_058202147.1 | *TSC10A* | *F*st |
| XP_058203022.1 | *PER64* | *F*st |
| XP_058221852.1 | *SHM3* | *F*st |
| XP_058198111.1 | *AERO1* | *F*st |
| XP_058192344.1 | *At5g08350* | *F*st |
| XP_058223406.1 | *KCS1* | *F*st |
| XP_058192067.1 | *FATB* | *F*st |
| XP_058209416.1 | *GSTU1* | *F*st |
| evm_TU_LG04_776 | *CML3* | Transcriptome |
| evm_TU_LG02_1189 | *ERD15* | Transcriptome |
| evm_TU_LG10_492 | *PP2CA* | Transcriptome |
| evm_TU_LG09_896 | *DREB1A* | Transcriptome |
| evm_TU_LG09_897 | *DREB1B* | Transcriptome |
| evm_TU_LG10_366 | *DREB2C* | Transcriptome |
| evm_TU_LG12_1974 | *HVA22E* | Transcriptome |
| evm_TU_LG05_2012 | *BAM3* | Transcriptome |
| evm_TU_LG10_1876 | *BAM1* | Transcriptome |
| evm_TU_LG09_2377 | *DPE2* | Transcriptome |
| evm_TU_LG07_2803 | *MSSP2* | Transcriptome |

**Table S14. Significant gene information screened by different analysis** (continued)

| **Gene ID** | **Coding protein name** | **Screening method** |
| --- | --- | --- |
| evm_TU_LG05_146 | *ADC2* | Transcriptome |
| evm_TU_LG11_1355 | *DGK2* | Transcriptome |
| evm_TU_LG09_401 | *GPC1* | Transcriptome |
| evm_TU_LG09_2177 | *SYP121* | Transcriptome |
| evm_TU_LG02_1078 | *FAD2* | Transcriptome |
| evm_TU_LG01_2484 | *CHIP* | Transcriptome |
| evm_TU_LG11_1332 | *NAC048* | Transcriptome |
| evm_TU_LG01_2676 | *MYB14* | Transcriptome |
| evm_TU_LG06_1688 | *ERF053* | Transcriptome |
| evm_TU_LG13_1650 | *APRR1* | Transcriptome |
| evm_TU_LG09_2607 | *CPK7* | Transcriptome |
| evm_TU_LG11_2204 | *CPK28* | Transcriptome |
| evm_TU_LG01_2195 | *MPK3* | Transcriptome |
| evm_TU_LG12_2024 | *ICE1* | Transcriptome |
| evm_TU_LG06_695 | *ZAT10* | Transcriptome |
| evm_TU_LG09_838 | *LTI65* | Transcriptome |
| evm_TU_LG08_2462 | *EDL3* | Transcriptome |
| evm_TU_LG10_208 | *ERF025* | Transcriptome |
| evm_TU_LG05_354 | *ERF109* | Transcriptome |
| evm_TU_LG08_335 | *NHL13* | Transcriptome |

**Table S15. The primers used in the PCR**

| **Gene symbol** | **gene ID** | **Primer sequence (5' →3'; forward/reverse)** |
| --- | --- | --- |
| *RmoEF1α* | evm.model.LG05.689 | GCTGCAAATTTCACATCCCAG/GCTCCTTCTCAATCTCCTTACC |
| *RorEF1α* | evm.model.LG04.607 | AGAATGTGGCAGTGAAGGATC/CCTTCTCAATCTCCTTACCCG |
| *Rmo18SrRNA* | evm.model.LG13.794 | GCTATGCGTGCTGGATTTG/TTTCCTTGGTCTATGCCTGTC |
| *Ror18SrRNA* | evm.model.LG13.1554 | CGAGGCCAGAGGTACAAGC/CCTGAACCGCAACCAATGT |
| *RmoAAA-ATPase* | evm.model.LG04.2701 | GTGGAGACAGAAGGGAAGATTG/TGGAAGGTGCAGTAAGACATG |
| *RorAAA-ATPase* | evm.model.LG06.319 | GATGACAAGATTACTCTGTCCGG/AATGTGAAGGTCCATGCGA |
| *RmoDREB1E* | evm.model.LG04.2702 | GGCACCCGGTTTACAGAG/TTCCTTATGTGTTCCGCCTC |
| *RorDREB1E* | evm.model.LG06.318 | CGGTTTCGAAGGATGCGGAA/CGAACCATCATCAGGGGAGAC |
| *RmoGPC1* | evm.model.LG06.2652 | GAGTGACGAAGAGATAATGGGTG/ATGTGTAGGGAATATCTTGCGG |
| *RorGPC1* | evm.model.LG09.401 | GCTGATTGTTTGGCGTTGTAG/AGTTCCGTCAGGATGCATG |
| *RmoCPK1* | evm.model.LG12.1809 | GTTCTTAGCTGCCACATTGC/AACACCTGCATTTCCCATTTG |
| *RorCPK1* | evm.model.LG09.1863 | CTTCTCGTCTGATCCTTGGC/GGTGCTGGGCTATAACTCTG |
| *RmoCPK7* | evm.model.LG11.2700 | TGATCCAGACCCAATTAAGCG/CCTCCACTGATAAATGCTCTGC |
| *RorCPK7* | evm.model.LG09.2607 | AGGATGCGGCTGGATTAAAG/CTCCCCGTAGTTCAAATGTCC |
| *RmoCPK28* | evm.model.LG07.74 | TTGAGGATGTTAAGCGAGAGG/CACTCGGCTGCAACTTTAAG |
| *RorCPK28* | evm.model.LG11.2204 | GGTTCAGAGTAGCAGTAAGACG/TCTCGCTTAACATCCTCAACAG |
| *RmoMPK3* | evm.model.LG02.826 | CGACCAACTTCAGAGAACGAG/TCCTAACAAACCCAAGATCCG |
| *RorMPK3* | evm.model.LG01.2195 | TGGAATTGTCTGCTCGGTG/TGAAGATCAGTGTCCATGAGC |
| *RorDREB1A* | evm.model.LG09.896 | GTTTTCGGGATGCCAAGATTG/TTCCATGTCATCTCCATCACC |
| *RmoDREB1B* | evm.model.LG12.893 | TCGTGGAAACCGGTCAAATG/GCTTCTTCGGGTGGCTGGA |
| *RorDREB1B* | evm.model.LG09.897 | GTTTTCGGGATGCCAAGATTG/AAGTTCCATGTCATCTCCACC |
| *RorDREB1B* | evm.model.LG09.899 | TTTTCGTCATCGTTCTGGTC/CTCACCTCGCACACCCAC |
| *RmoDREB2C* | evm.model.LG10.2165 | CGAAAGGATTGCATTGATTGG/ACAGCATCGGCTCCACCAC |
| *RorDREB2C* | evm.model.LG10.366 | TGTCGTCTGGAATCGAAAGG/CTGGCTTGGAATCTCCGTTAG |
| *RmoEDL3* | evm.model.LG11.276 | GGGATTTATGAAGTCGAGGACG/ACACGAAATACTCCAAACCCC |
| *RorEDL3* | evm.model.LG08.2462 | ACCAGGGCGTGTTTGATC/CTCCTTATCCGATCCAGCTTC |
| *RmoLTI65* | evm.model.LG12.831 | ATCTCGTATGCCACTTCTGC/TTTTCCAATCTCTTCCCCTCC |
| *RorLTI65* | evm.model.LG09.838 | AAAGGCGAGGAAGGTGAAG/CCGTGTATTTGGAACCGTATTG |
| *RmoSYP121* | evm.model.LG12.2146 | CAGGTGTTTCTCGACATGGCG/CAGACCACTTGCGGGTGTTCT |
| *RorSYP121* | evm.model.LG09.2177 | GCAAGGTGAACAAATCGACG/GAACAGAACCACAAAGAGGATG |
| *Actin2* | − | TGCCAATCTACGAGGGTTTC/TTCTCGATGGAAGAGCTGGT |
| *L25* | − | AGTTACATTCCACCGACC/TCCTCAATCTTCTTCATTGCAG |
| Rni-p1p2 | evm.model.LG10.146 | AGTATTGTTGTTGTTTTCC/AGTTGTGTACATGGCTGT |
| Rpri-p1p2 | evm.model.LG12.2493 | AGTATTGTTGTTGTTTTCC/AGCTGTGTACATGGCTGT |
| Rlau-p1p2 | evm.model.LG08.149 | AGTATTGTTGTTGTTTTCC/AGTTGTGTACATGGCTGTCA |
| Ror-p1p2 | evm.model.LG08.160 | AGTATTGTTGTTGTTTTCC/AGTTGTGTACATGGCTGT |
| Rov-p1p2 | Ro09556.1 | AGCATTGTTGTGTTTTCC/AGCTGTGTACAGGGCTGT |
| p3p4 | − | ATGGCGATCGCAGCAGCG/TCATTGAAGCAGATTAGCTTTCAAAGG |
| Rov-p3p4 | − | ATGGCGATCGCTGCAGCG/TCATTGAAGCAGATTAGCTTTCAAAGG |

**Other Supporting Information for this manuscript includes the following:**

**Dataset S1. Re-sequencing accessions information used in this study.**

**Dataset S2. GO enrichment information of core and dispensable gene families.**

**Dataset S3. KEGG enrichment information of core and private gene families.**

**Dataset S4. Global distribution information of 18 species of rhododendron.**

**Dataset S5. GO enrichment information of unique genes of *Rhododendron* species at high and low altitudes.**

**Dataset S6. KEGG enrichment information of unique genes of *Rhododendron* species at high and low altitudes.**

**Dataset S7. KEGG enrichment information of genes affected by SVs.**

**Dataset S8. KEGG enrichment analysis of genes influenced by unique LTRs from *R. simsii*, *R. molle*, *R. principis*, and *R. fortunei*.**

**Dataset S9. Download link for the public resequencing data used in this study.**
